# Preliminary Investigation on Vacancy Filling by Small Molecules on the Performance of Dye-Sensitized Solar Cells: The Case of a Type-II Absorber

**DOI:** 10.3389/fchem.2021.701781

**Published:** 2021-07-08

**Authors:** Francis Kwaku Asiam, Nguyen Huy Hao, Ashok Kumar Kaliamurthy, Hyeong Cheol Kang, Kicheon Yoo, Jae-Joon Lee

**Affiliations:** Department of Energy and Materials Engineering, Research Center for Photoenergy Harvesting & Conversion Technology (phct), Dongguk University, Seoul, South Korea

**Keywords:** catechol, Z907, vacancy, co-sensitization, dye-sensitized solar cells and thermodynamics

## Abstract

The steric shielding offered by sensitizers on semiconducting surfaces as a result of branching in the dyes used offers the less utilization of semiconducting substrate sites during device fabrication in dye-sensitized solar cells (DSSCs). This work proposes a strategy to increase the coverage through the utilization of small molecules which have the ability to penetrate into the sites. The small molecules play the dual role of vacancy filling and sensitization, which can be viewed as an alternative to co-sensitization also. Hence, we show for the first time ever that the co-adsorption of catechol with Z907 as a sensitizer enhances the electron density in the photo-anode by adsorbing on the vacant sites. Catechol was subsequently adsorbed on TiO_2_ after Z907 as it has a stronger interaction with TiO_2_ owing to its favorable thermodynamics. The reduced number of vacant sites, suppressed charge recombination, and enhanced spectral response are responsible for the improvement in the PCEs. Quantitatively, both organic and aqueous electrolytes were used and the co-sensitized DSSCs had PCE enhancements of 7.2 and 60%, respectively, compared to the control devices.

## Introduction

Increase in fossil fuels, such as oil and coal consumption, and consequently the gradual decline of their natural deposits have emitted into the atmosphere various greenhouse gases (CO_2_, NO_x_, SO_2_, *etc.*) that contribute directly to global warming. Therefore, many countries are focused on harvesting renewable energy sources including solar, wind, and biomass to reduce the dependency on fossil fuel. Silicon solar cells proved to be alternatives, but researchers developed interest in even cheaper alternatives known as dye-sensitized solar cells (DSSCs) when these photovoltaic systems realized a breakthrough efficiency of 7% (O’Regan and Grätzel, 1991; Grätzel, 2001). Currently, dye-sensitized solar cells (DSSCs) which convert sunlight directly into electricity have received significant scientific attention due to their simple fabrication process, low production cost, and low toxicity (O’Regan and Grätzel, 1991).

**GRAPHICAL ABSTRACT:**
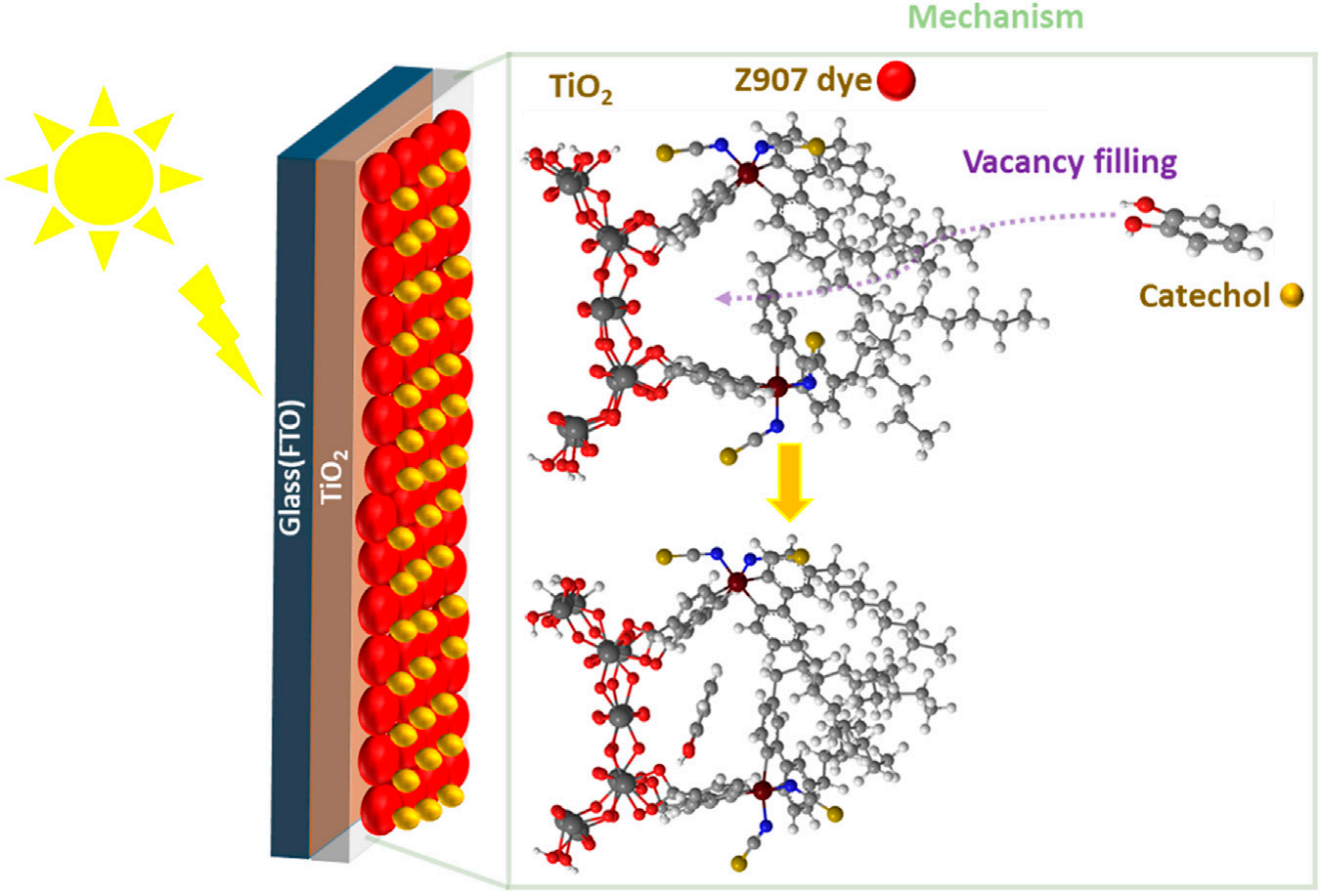


Generally, a titanium (IV) oxide or TiO_2_-based DSSC is composed of three main parts: anode, cathode, and redox electrolyte (Fujisawa and Hanaya, 2018). The fundamental component of an anode is a mesoporous TiO_2_ semiconducting film coated on a fluorine-doped tin oxide (FTO) glass (Boschloo and Hagfeldt, 2009; Lee et al., 2011; Sun et al., 2017). The TiO_2_ layer serves as an anchor for the monolayer of photosensitizer or dye molecules. The cathode is often composed of an FTO substrate with a thin layer of platinum on its surface. (Ghann et al., 2017; Nguyen et al., 2018). The redox electrolyte, typically iodide/triiodide (I^−^/I_3_^−^) couple, fills the gap between the anode and the cathode. When DSSCs are exposed to visible light, electrons are excited from the occupied molecular orbitals (HOMO, HOMO-1, *etc.*) into the unoccupied molecular orbitals (LUMO, LUMO+1, *etc.*; Chen and Lin, 2017). After that, these excited photoelectrons travel to the conduction band of TiO_2_ due to thermodynamic matching of band levels, then migrate to the anode, and transfer to the cathode through an external circuit. At the cathode, electrons are collected by the redox electrolyte and brought to the oxidized dye species to regenerate dye molecules. (Boschloo and Hagfeldt, 2009; Sánchez-de-Armas et al., 2011; Chen and Lin, 2017; Rahman et al., 2018; Deb Nath and Lee, 2019). Among the main components of DSSCs, the photosensitizer plays an important role in the harvesting and conversion of solar energy to electricity (Ooyama and Harima, 2012; Ooyama et al., 2014). Therefore, many studies on dyes have been reported for DSSC applications in the last few decades. For example, many research groups have introduced natural dyes which are extracted from plant components to cut down the high cost and chemical synthesis instead of using ruthenium-based photosensitizers (Elangovan and Venkatachalam, 2015).

Co-sensitization has been useful to increase PCE (%) of DSSCs. Co-sensitization of N719 dye with organic dyes has enhanced the spectral range from the visible to near-infrared (NIR) region in order to increase the absorption on TiO_2_ (Zhang et al., 2011; Elangovan and Venkatachalam, 2015; Luo et al., 2016; Mazloum-Ardakani and Arazi, 2019; Wu et al., 2020). It is also useful to co-sensitize two metal-complex dyes, but they usually have bulky groups, which makes the utilization of active sites on the semiconductor less effective. (Dehghani, 2013; Kumar et al., 2019). Quantum dots have also proven to be suitable for integration into DSSCs for co-sensitization as separate layers on the TiO_2_, and this enhances the utilization of UV and NIR light with high quantum efficiency, as quantum dots have tunable bandgaps during synthesis (Li et al., 2013; Elangovan and Venkatachalam, 2015; Sun et al., 2017). Other works have also focused on monolayer adsorption by suppressing dye aggregation on TiO_2_ surface through co-adsorption with deoxycholic acid (DCA), stearic acid (SA), or chenodeoxycholic acid (CDCA) (Ren et al., 2010; Lim et al., 2011). Despite the use of different molecules for co-sensitization, there is also the exploitation of multiple anchoring of single sensitizers so as to achieve considerable interaction and electron injection for high-efficiency devices (Yeh-Yung Lin et al., 2014). These strategies have made a great contribution to improve the efficiency of DSSCs.

Among the components of DSSCs, the electrolyte system is also one which plays a critical role in the transport of charges between the cathode and anode. As the interest is toward obtaining very cheap photovoltaics for DSSCs, it is necessary to achieve a device very close to the natural photosystem. Liquid electrolyte systems with organic solvents pose the long-term operational problem of organic solvent volatility and contamination with water in ambient environmental conditions (Bella et al., 2015). It is therefore important to consider the use of water as a solvent for the electrolyte system, which can withstand humidity and thermal fluctuations on the scale of commercialization. Unfortunately, purely water-based DSSCs have poor performances compared to organic solvent–based ones. The limiting factors of those include dye-desorption, water reaction with semiconducting (TiO_2_) surface leading to high recombination, low current collection, and slower kinetics of charge transport (Bella et al., 2015). Notwithstanding, there have been significant progress in tackling these problems. One of which includes the use of hydrophobic dyes like Z907, so as to avoid desorption of dyes from the TiO_2_ surface and the recombination of photo-anode electrons with the electrolyte (Law et al., 2012). It is therefore of interest to explore other sensitizing strategies in aqueous DSSCs.

Recently, catechol-based sensitizers such as dopamine and fluorone have received much attention because of their application in DSSCs (Mosurkal et al., 2004). Catechol-based DSSCs are classified as Type-II DSSCs, as the photoelectrons from the occupied molecular orbitals of the sensitizer jump directly into the conduction band of TiO_2_ electrode (Xu et al., 2007; Li et al., 2009; Sánchez-de-Armas et al., 2011). According to Ooyama et al., the advantage of catechol-based DSSCs as compared to traditional DSSCs is the efficient light-harvesting feature over a wide range of the solar spectrum (Ooyama et al., 2015a). This is because the direct photoelectron injection pathway in Type-II DSSCs can happen in the long wavelength area (near infrared) (Persson et al., 2000; Mowbray and Migani, 2016). However, the main challenge with using catechol as a photosensitizer in DSSCs is overcoming the fast charge recombination process, taking place in the sub-picosecond timescale as compared to μs−ms in conventional or Type-I DSSCs (Ooyama et al., 2016). Therefore, the sunlight to electricity conversion efficiency of catechol-based DSSCs is currently relatively low, *ca.* 1.3% maximum (Tae et al., 2005). Several metal-complex dyes have a bulky structure, and this induces steric shielding during sensitization. In that, the bulky structures make it impossible for more of the molecules to adsorb completely on TiO_2_ very close to each other, which then creates empty sites on the TiO_2_ surface that serve as electron traps during charge transport. A novel strategy will be to utilize certain small molecules which can penetrate into these sites without replacing the already adsorbed dyes.

**FIGURE 1:**
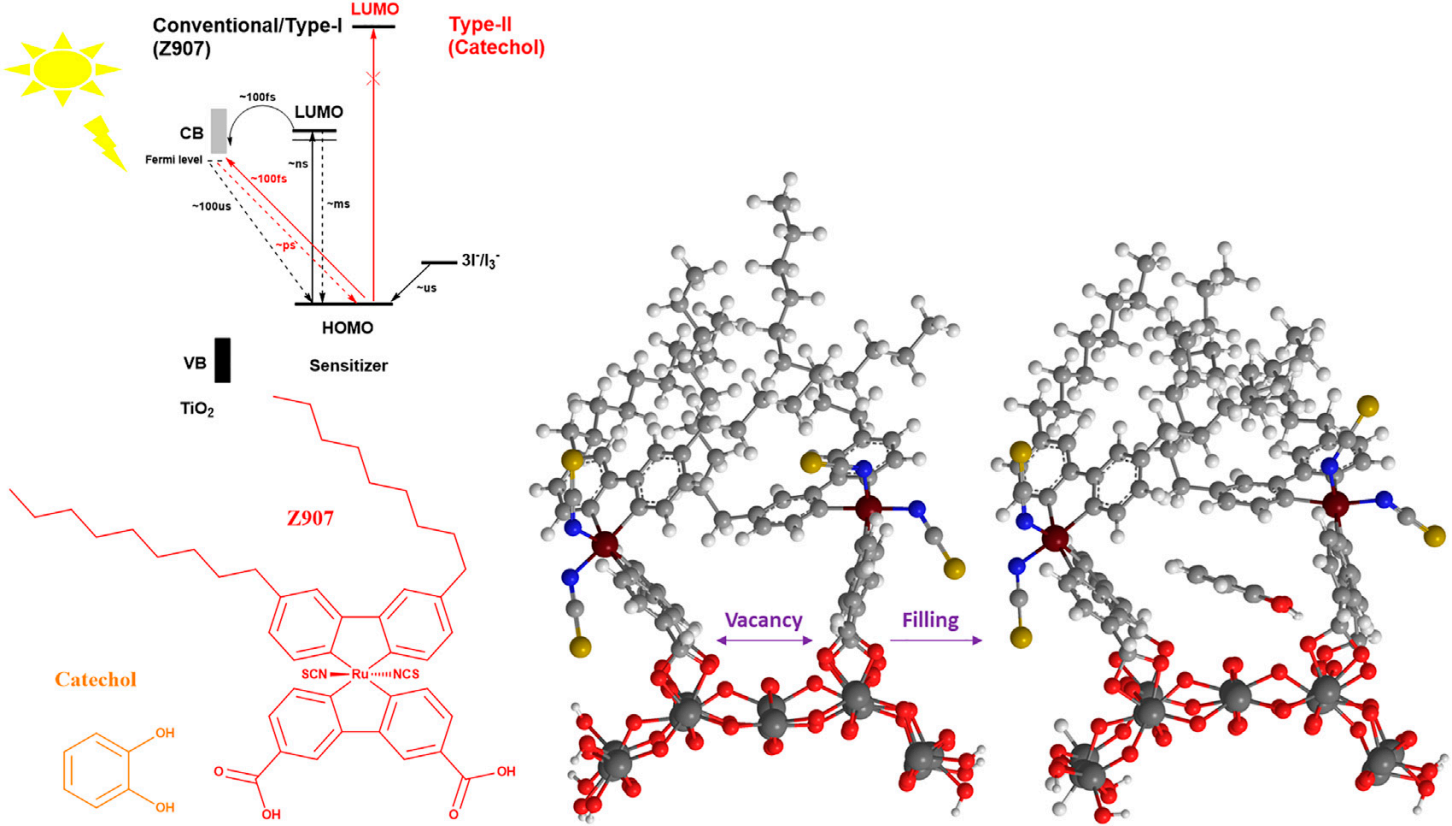
Schematic illustration of the steric induced vacancy as a result of bulky side chains on Z907 when adsorbed on the TiO_2_ sites and the filling of those sites by the small catechol sensitizer. Insert describes the kinetics and thermodynamics between the two sensitizers.

In this work, catechol, a small molecule, is co-adsorbed with Z907 dye (bulky) as it has the potential to induce light harvesting over a wide region (400–600 nm). Also, catechol can penetrate into the vacant sites without replacing much of the Z907 molecules so as to improve the electron density in the photo-anode, thus reducing the interfacial charge transport resistance and enhancing the efficiency of the DSSCs. This is due to the ability of this small molecule to occupy vacant sites of the semiconductor material, which would otherwise have served as active sites for either solvent molecules or electrolyte molecules to recombine with electrons injected into the photo-anode upon irradiation. The use of a deep ultraviolet light absorber with the bulky dye molecule introduces two injection phenomena in the devices, termed as Type-II and Type-I DSSC kinetics. The former representing a direct injection mechanism and the latter involving a dye-excited state (Figure 1). The comparative sizes of the two sensitizing molecules make the realization of the filling effect feasible, compared to what is expected of two bulky molecules. The present work demonstrates a novel approach to co-adsorption and co-sensitization from a structural perspective compared to the well-known conventional competitive co-adsorption methods and realized the improved power conversion efficiency of 5.44%.

## Experimental Section

### Materials and Chemicals

Reagent grade 1,2-dihydroxybenzene (catechol, 99+%), iodine (I_2_) >99.8 purity, guanidinium thiocyanate (GuSCN >97%), 1-methylimidazole (>97%), polyethylene glycol (PEG-300), lithium iodide (LiI, 99.9%), 4-tert-butylpyridine (tBP, 96%), acetonitrile (ACN, 99.8%), valeronitrile (VLN, 99.5%), and hydrogen hexachloroplatinate (IV) hexahydrate (H_2_PtCl_6_∙H_2_ O,≥37.5%) were all purchased from Sigma-Aldrich. Fluorine-doped tin oxide (F-SnO_2_,14 Ω/sq.) glass, 1-iodopropane (>98%, TCI), cis-Bis(isothiocyanato) (2,2′-bipyridyl-4,4′-dicarboxylato) (4,4′-di-nonyl-2′-bipyridyl) ruthenium (II) (Z907, Organica), potassium iodide (KI, 99.5%, D.S.P), toluene (>99.5%, Daejung), and titanium (IV) oxide paste (20 nm, Head Solar, Korea) were purchased from different suppliers. All chemicals were used as purchased.

### Fabrication of Dye-Sensitized Solar Cells

First, the FTO substrates were washed by sonicating in a solution (ethanol, isopropanol, and acetone in the ratio of 1:1:1) for 30 min, air-dried, and UV–ozone–cleaned for 30 min. A TiO_2_ blocking layer solution which was prepared from 0.15 M titanium isopropoxide in 2-propanol was spin-coated on FTO at 1,500 rpm for 15 s, and sintered at 450°C for 15 min. After that, the TiO_2_ paste (20 nm) was coated by screen printing to about 4 μm thickness. The TiO_2_ paste-coated FTO was then kept in a clean box with ethanol vapor environment to reduce the surface irregularity. Next, the electrodes were sintered at 500°C using optimized ramping and soaking process for 30 min to remove the organic binder from the TiO_2_ paste and achieve good electrical contact between FTO substrate and TiO_2_, as well as improve the mechanical strength of the TiO_2_ layer.

In the next step, the TiO_2_ electrodes were trimmed to an active area of 0.3 cm^2^ and immersed in different sensitizing solutions of 0.1 mM catechol (30 min in the dark) and 0.3 mM Z907 (5 h with automated shaking at 40°C), separately. A sequential approach was used for co-sensitization, which involved dipping the TiO_2_ films first into 0.3 mM Z907 solution (5 h), followed by 0.05 mM catechol solution (30 min). After that, a Pt-counter electrode was prepared by spin-coating 10 mM solution of hexachloroplatinate (IV) hexahydrate (in ethanol) on FTO with an rpm of 2000 for 30 s and annealed at 500°C for 15 min. Finally, the TiO_2_ electrodes were assembled with the Pt-counter electrodes by sandwiching with 25 μm surlyn and thermally sealed with an upper temperature of 100°C and bottom temperature of 80°C for 7 s, then filled with an aqueous electrolyte solution (KI 1 M, I_2_ 0.008 M, GuSCN 0.05 M, PEG 300-0.5%, H_2_O) or organic electrolyte solution (0.6 M PMII, 0.03 M I_2_, 0.1 M LiI, 0.5 M tBP, ACN: VLN 85: 15) to form DSSCs.

### Photovoltaic, Optical, and Electrochemical Measurements

The UV-visible absorption spectra of the samples were measured with a Scinco, S-3100 spectrophotometer. Photovoltaic measurements were performed under simulated light of one Sun (1000 Wm^−2^) using McScience Polaronix K201. The TiO_2_ film thickness was measured using a KLA Tencor Alpha-step profiler, D-300 Stylus. IPCE measurements were performed with the McScience K3100 spectral IPCE measurement system. Electrochemical impedance measurements were performed with a Princeton Applied Research VersaSTAT 3 system.

### Computational Studies

The atomistic-scale molecular mechanics (MM2) level of theory minimizations were performed for the structures in the gaseous phase. The MM2 code used (Allinger, 1977) was as integrated in the CHEM3D 17.0 (PerkinElmer Informatics Inc.) software on a 64-bit operating system desktop computer, with 2.90 GHz Intel (R) Core (TM) i5-9400 CPU in Windows 10 platform. Molecular dynamics (MD) code (Nosé, 1984) also integrated in the software was used to initially relax the structures all at 298.15°K. CHEM3D 17.0 served as the visualization interface to the codes.

## Results and Discussion

### Molecular Mechanics Prediction

**FIGURE 2:**
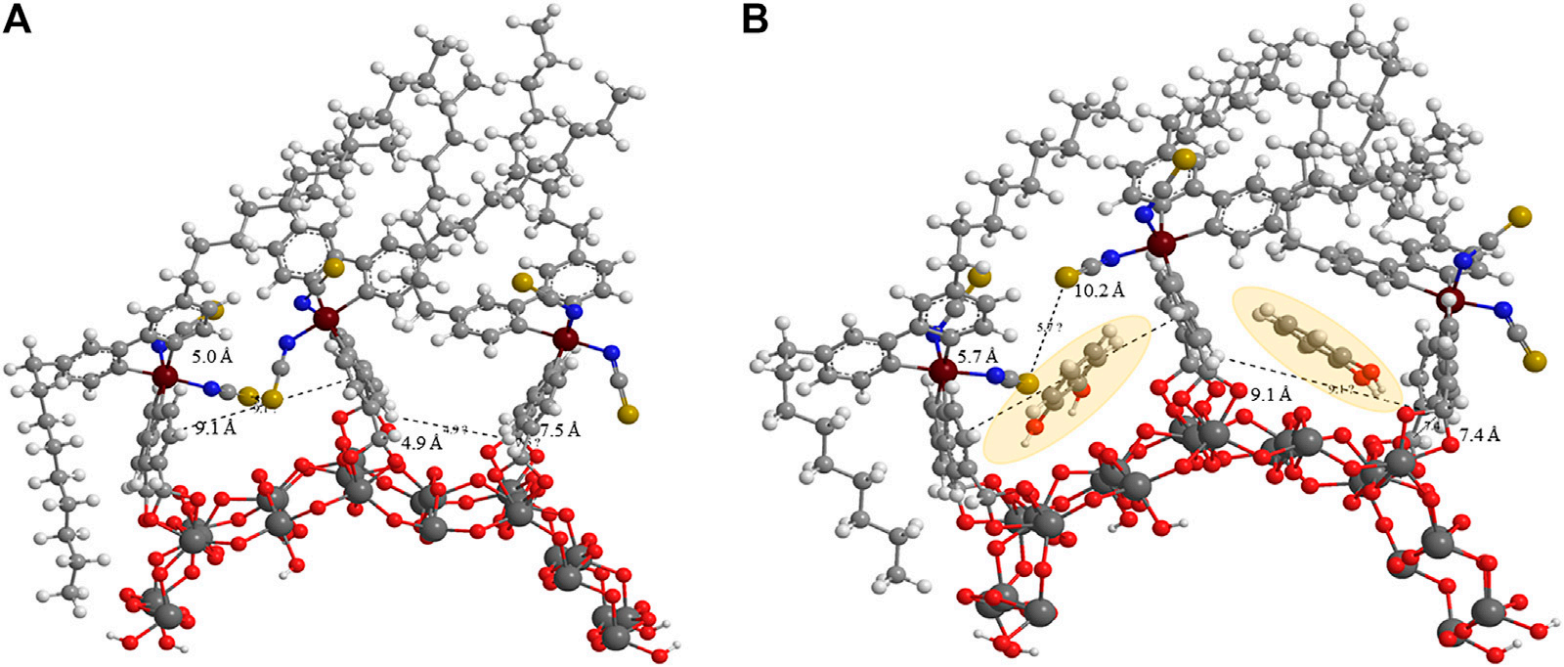
MM2 force field prediction of the **(A)** steric induced vacancy as a result of bulky side chains on Z907 when adsorbed on the TiO_2_ sites and **(B)** the filling of those sites by two small catechol sensitizers as shaded.

MM2 force field energy minimizations for three Z907 molecules anchored together on (TiO_2_)_24_ slab with each pair representing a different anchoring phase were performed. One phase involves the isothiocyanate groups of the two Z907 molecules facing each other, while the other involves them facing away from each other. This was integrated into CHEM3D 17.0 for visualization purposes. The implication of this calculation is that electronic motions were not considered in the potential energy curve, but the results are as a result of the atomistic nuclear positions owing to the very large size of the grid (Allinger, 1977). Two ends of the carboxylic acids on each Z907 molecule were anchored to the (TiO_2_)_24_ slab and minimization performed to RMS move and gradient of 0.0001. From Figure 2A, both optimized phases on the (TiO_2_)_24_ surface showed vacancies which could accommodate the catechol molecule. Catechol molecules were inserted into these vacant sites, and the effects on the grid sizes were monitored, but no significant changes appeared as shown in Figure 2B.

**FIGURE 3:**
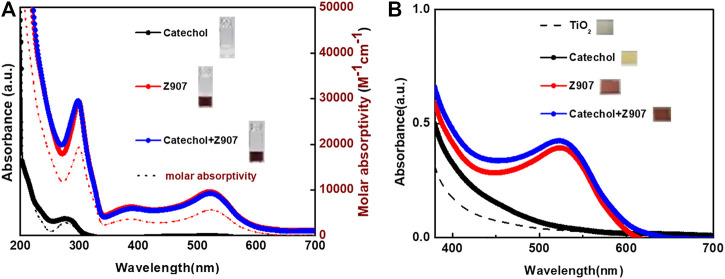
UV-Visible absorption spectra of sensitizers both **(A)** in ethanol (molar absorptivity axis applies to the dotted lines) and **(B)** adsorbed on TiO_2_ films, respectively. Inserts are photographs.

Quantitatively, the distances between selected atoms around the vacant sites before and after the filling were measured and the changes monitored. The S–S (gold) group distance changed from 5.0 Å to 5.7 Å, while the C–C distance between two aromatic groups of the other phase decreased from 7.5 Å to 7.4 Å. A careful look at Figure 2 shows that the space does not increase much with the catechol molecules freely suspended at the interstitial sites, indicating ease of penetration unto TiO_2_ sites. Anchoring of catechol typically introduces stricter orientation, making the possibility of steric instabilities nonexistent as the freely oriented molecule is easily accommodated in the vacancy created. This is supported by the decrease in total energy of the frame from 661.441–346.256 kcal mol^−1^ after inserting two catechol molecules into the grid. This represents a drastic drive for migration of catechol into the sites during sensitization. This is in good agreement with the observed increase in the UV-Visible spectral response, as shown in Figure 3B, and number of molecules was calculated for the increased coverage as given in electronic supplementary information (ESI) (Supplementary Table S2). Since a real DSSC works in the condensed phase, the effect of solvation on the structures was investigated, but no significant changes were observed. The detailed parameters investigated are presented in ESI (Supplementary Figures S4,S5; Supplementary Tables S3,S4).

### UV-Visible Spectral Analysis

The photographs of the sensitizers in ethanol, insert of Figure 3A, together with their UV-Visible spectra, Figure 3A, reveal that catechol is a deep UV-absorber with maximum absorption at 278 nm and Z907 is a visible light absorber with maximum absorption at 524 nm. This confirms the use of a dye (Z907) and small molecule (catechol) in this study. After dipping the TiO_2_ films into the sensitizing solutions, it is confirmed from the photographs, insert of Figure 3B, and UV-Visible spectra, Figure 3B, that all the films are absorbing visible light. Z907 is a dye which is adsorbed on TiO_2_, but catechol shows a different phenomenon which is explained by the previous reports (Xu et al., 2007; Sánchez-de-Armas et al., 2011). Here, the catechol molecule binds to TiO_2_ strongly to form a new absorbing complex in the visible region due to strong electronic coupling between the two. This induces what is known as Type-II DSSC mechanism, involving the absorption of visible light to excite electrons from the occupied molecular orbitals of the catechol directly into the TiO_2_ conduction band without the involvement of a catechol-LUMO state. This helps eliminate the losses of LUMO-HOMO recombination after excitation and gives a 100% charge transfer. This is evident from the enhanced absorption of the Z907 + catechol co-sensitized film from 400 to 550 nm compared to that of the Z907 film.

The absorbance spectra of Z907 + catechol mixture dissolved in ethanol showed similar absorption maximum to those of Z907 in the 350-700 nm region. On the contrary, when comparing the absorption in the region 250-300 nm with that of only Z907, an increase in absorption is observed. This is because the absorption of catechol in pure solvent at 278 nm contributes to the increase. Applying this observation and the idea that catechol induces visible absorption on TiO_2_ explains why such an interaction is responsible for the observation with the sensitized films. Nevertheless, previous attempts to co-sensitize TiO_2_ with Type-I/Type-II phenomenon proved unsuccessful (Ooyama et al., 2015b, Ooyama et al., 2016). This is because catechol bound to the TiO_2_ system is thermodynamically favorable than dyes with carboxyl anchors, so in the process of co-sensitization, strict attention has to be paid to the kinetics and thermodynamics. This and the fact that the catechol-TiO_2_ system has a limitation of very fast back-electron transfer explain those failures till date. That is why this work presents a breakthrough in this kind of co-sensitization approach.

Upon understanding this issue, a different strategy was employed in this co-sensitization research. First, the TiO_2_ films were sensitized with Z907, followed by immersion of the Z907-TiO_2_ films in the catechol solution. The concentrations of the sensitizing catechol solutions were optimized between 0.025 and 0.1 mM to avoid the replacement of the Z907 molecules on the TiO_2_ film. Higher concentrations induced faster replacement of the Z907 within a short period of 30 min. Also, co-sensitization time of 30 min was employed. The combined strategy of few catechol molecules in solution, coupled with very short timescale, was to avoid replacement of Z907 dyes. This will give enough time for the catechol molecules to penetrate into the vacant sites to enhance utilization of the TiO_2_ active sites as indicated by the absorbance increase in Figure 3B and modeled Figure 2. ESI Supplementary Table S2 details the number of Z907 dye molecules detached from the TiO_2_ surface upon co-sensitization with catechol during optimization. As the concentration of catechol was increased in solution, the replacement of Z907 also increased as indicated in ESI Supplementary Table S2.

### Photovoltaic Performance Analysis

Two kinds of DSSCs were fabricated. The photovoltaic performance of the Type-II sensitization mechanism is studied for the first time in aqueous environment due to their stronger binding interaction with TiO_2_ than conventional dyes: one group with an organic electrolyte and the other set with an aqueous electrolyte. The difference between these devices is the electrolyte only; all other components were kept the same, including the co-sensitization conditions. The sensitization process was carefully done with a hydrophobic dye (Z907) as per this study (Jeon et al., 2017). The co-sensitization process optimization was performed in an aqueous electrolyte, and the best combination was employed for sensitization in an organic electrolyte also. The optimization was performed in an aqueous electrolyte because of the need to avoid desorption of Z907 and the optimized condition subsequently used for the organic electrolyte (Bella et al., 2015). Polyethylene glycol was used in the aqueous electrolyte system as a binder (Mozaffari et al., 2014).

The PCE (%) of only catechol-DSSC (0.1 mM) in aqueous electrolyte is 0.05% and that of only Z907-DSSC is 0.52%, as shown in ESI Supplementary Table S2. Since the catechol system has a faster back-electron transfer, replacing the Z907 on the TiO_2_ sites will give lower PCE (%). The highest concentration of catechol, that is, 0.1 mM, replaced 1.90 mg cm^−2^ of Z907 on the TiO_2_ surface, and this is in agreement with the lowest PCE (%) of 0.51% obtained in ESI Supplementary Table S2. On the contrary, the lowest concentration of catechol, that is, 0.025 mM, replaced only 1.42 mg cm^−2^ of Z907 on the TiO_2_ surface, which is why it gave a PCE (%) of 0.54%. This implies that the small concentration enabled few of the catechol molecules to penetrate through the surface into the vacant sites without replacing much Z907 molecules as shown in Figure 2. This is responsible for the slightly higher PCE (%). Interestingly, 0.05 mM of catechol which is double of 0.025 mM gave the best PCE (%) of 0.8% while replacing almost the same number of Z907 molecules as that of 0.025 mM, that is, 1.50 mg cm^−2^. It is evident from these observations that this concentration enabled penetration of a higher number of catechol molecules into the vacant sites while having almost no effect on the replacement of Z907 molecules on the TiO_2_ surface, as shown in ESI Supplementary Table S2. The PCE (%) increment observed here is drastic, 60%. Upon realizing this great achievement, the same concentration was used for co-sensitization in the organic electrolyte which is of particular interest. Notwithstanding this choice, some DSSCs were still fabricated with the other co-sensitization conditions in the organic solvent–based electrolyte.

**FIGURE 4:**
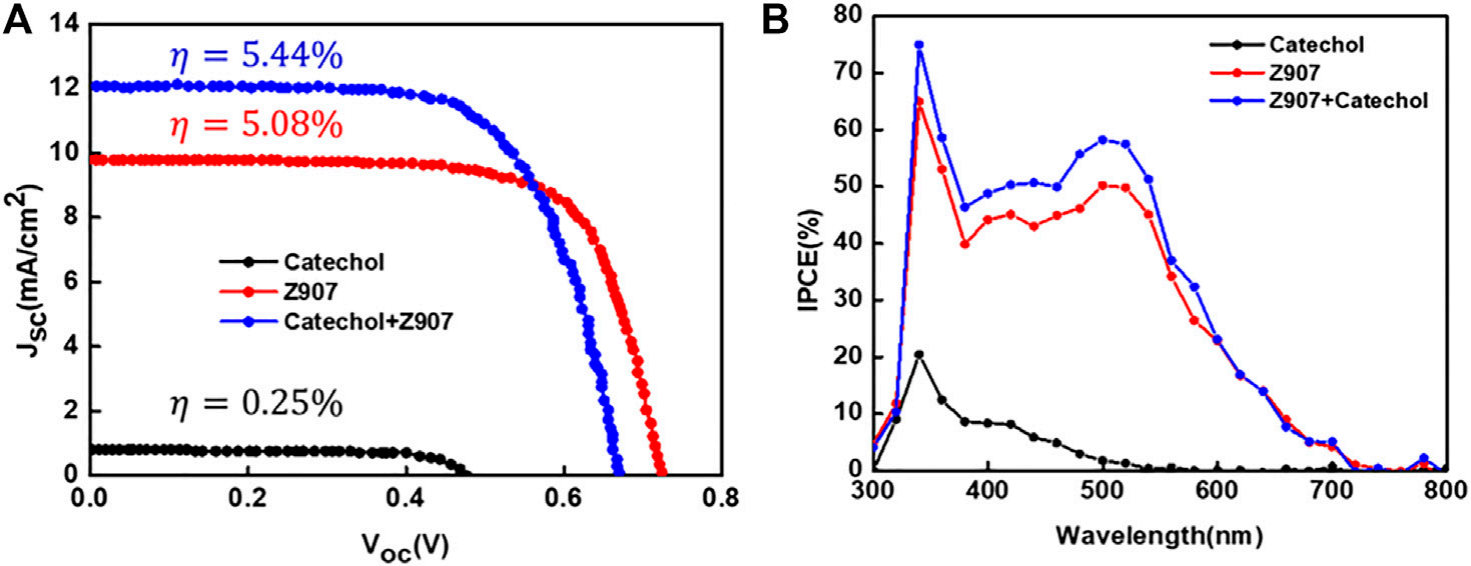
**(A)** Current–voltage plots and **(B)** IPCE spectra of representative devices fabricated with the sensitizers in organic solvent–based electrolyte under AM1.5G illumination (1sun).

From Figure 4A, the current density, J_sc_, for the co-sensitized DSSC is 12.09 mA cm^−2^, which is higher than that of the Z907 DSSC, 9.77 mA cm^−2^. As seen from Table 1, attachment of Z907 to the TiO_2_ surface gives a high V_oc_, and upon co-sensitization, the V_oc_ decrease is little, indicating no significant effect. The increase in electron density is associated with the current density enhancement due to higher surface coverage of TiO_2_ by co-sensitization. Incident photon-to-electron conversion efficiency (IPCE) is an important measurement tool to determine the external quantum efficiency (EQE) of the constructed solar cell devices covering a specific range of wavelengths. In a simple sense, this explains the degree of functioning of the constructed dye-sensitized solar cell devices in the particular wavelength range. Principally, EQE or IPCE is the ratio of the total number of photoelectrons produced in response to the quanta of photons striking the active area of the constructed solar cell (Younas and Harrabi, 2020).

**TABLE 1:** Photovoltaic, optical, and electrochemical responses of representative devices for the sensitizers in organic solvent–based electrolyte.

Sensitizer	$\lambda_{\max}\left(\mathrm{nm}\right)$	Ɛ^a^ (10^3^ M^−1^ cm^−1^)	R_s_ (Ω)	$R_{\mathrm{ct1}}\left(\Omega \right)$	$R_{\mathrm{ct2}}\left(\Omega \right)$	J_sc_ (mA/cm^2^)	V_oc_ (V)	FF (%)	η(%)
Catechol	278	2.8	5.0	240.0	8,473.0	0.76	0.48	67.6	0.25
Z907	524,388, 298	5.6,3.6,19.2	5.1	10.7	532.1	9.77	0.72	72.5	5.08
Catechol + Z907			5.4	3.0	282.3	12.09	0.67	67.7	5.44

a -Molar absorptivity of sensitizers in ethanol, R_s_ series resistance between FTO/TiO_2_, R_ct1_ charge transfer resistance between cathode and electrolyte, R_ct2_ electron transfer resistance among TiO_2_/dye/electrolyte.

From Figure 4B, the IPCE (%) of the catechol-DSSC shows light to current conversion in the region 300–550 nm which is associated with the current observed in the device. A careful look at the IPCE of the Z907 + catechol device shows an enhancement within the region 340–550 nm, which confirms the contribution of the active site current generation from catechol upon coverage. The increase of the IPCE in this region only supports the claim that the catechol molecules penetrate into the vacant sites with very little effect on replacement of the Z907 molecules. This vacancy filling contributed to 24% enhancement in the current density. The detailed photovoltaic performance is presented in Table 1. Also, to support this claim, the electron density calculation as presented in ESI Supplementary Table S1 was performed, where a higher density of 4.70 × 10^21^ is seen compared to 2.50 × 10^21^ for the Z907-only DSSC. Details of photovoltaic performances during optimization for both organic and aqueous solvent–based electrolytes are given as ESI, Supplementary Tables S1,S2; Supplementary Figure S2. The aqueous DSSCs show results which are in excellent agreement with those observed for the organic electrolyte–based DSSCs.

### Electrochemical Impedance Spectroscopy Analysis

**FIGURE 5:**
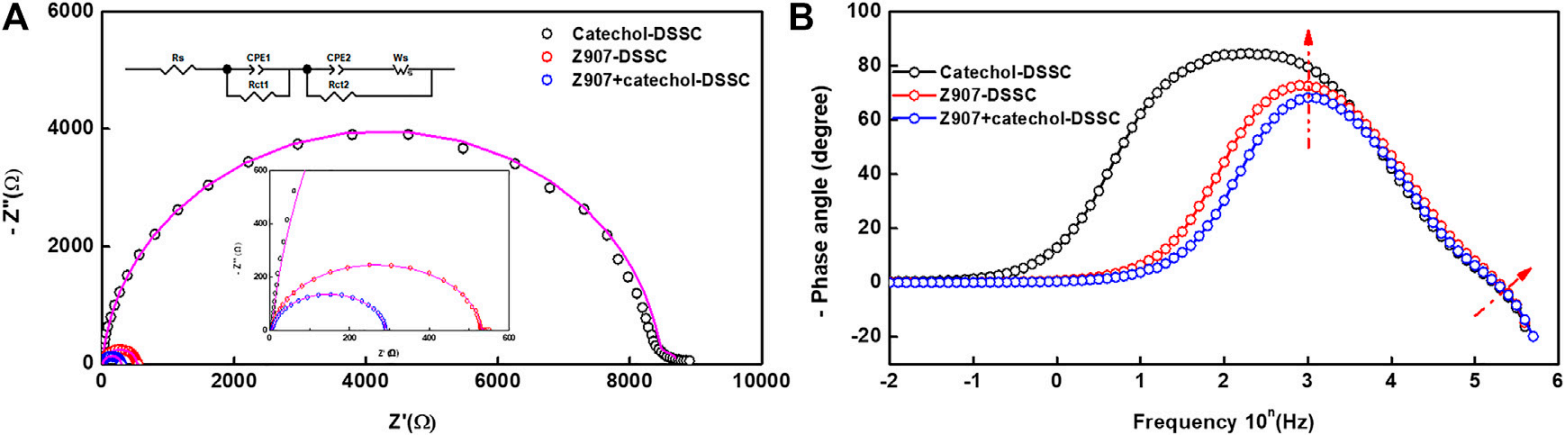
Electrochemical impedance spectroscopy (EIS) data. **(A)** Nyquist plot and **(B)** Bode phase plot of the devices in the dark employing potentials according to the open-circuit voltages. Insert is the Nyquist plot equivalent circuit diagram.

Interfacial charge transport kinetics were investigated at ambient condition for an applied potential (frequency scan of 1 MHz-0.01 Hz) according to V_oc_ of the devices (Mazloum-Ardakani and Arazi, 2019). Figure 5A represents the EIS-Nyquist plot which shows two semicircle behaviors corresponding to high and mid-frequency potentials for all the three different devices. Usually, three semicircles are observed in conventional DSSCs. These represent the resistances of the redox reaction at the platinum counter electrode interface in the high-frequency region (R_ct1_), the electron transfer at the TiO_2_/dye/electrolyte interface in the middle-frequency region (R_ct2_), and series resistance (which comprises the total interface resistance of electrolyte with FTO and sheet resistance of FTO) in the low-frequency region (R_s_) (Li et al., 2013). The obtained data in the present study were simulated with equivalent circuit modeling, and the electrochemical parameters are presented in Table1. In the present study, contribution of the major semicircle in the mid-frequency region (R_ct2_) is dominant, while the other resistances are very low in all the devices. The width of this semicircle represents the electron-transfer resistance at TiO_2_/dye/electrolyte interface (R_ct2_), which defines the recombination kinetics of the device. The electrolyte and FTO used in all the devices are similar, which accounts for nearly equal series interface resistance (R_s_ = 5 Ω) for all the devices. The charge transport resistances R_ct1_ and R_ct2_ are very low in the case of Z907 + catechol co-sensitized DSSC (R_ct1_ = 3 Ω; R_ct2_ = 282 Ω) than its individual counterparts (R_ct1_ = 11 Ω; R_ct2_ = 532 Ω for Z907-DSSC and R_ct1_ = 240 Ω; R_ct2_ = 8,473 Ω for catechol-DSSC). This reveals that efficient charge transfer occurs at Pt/electrolyte and TiO_2_/Z907 + catechol/electrolyte interfaces in the co-sensitized DSSC.

Nonetheless, the catechol-based device shows high interfacial charge transport resistances (R_ct1_ = 240 Ω and R_ct2_ = 8,473 Ω) which reduced the electron transport properties and significantly suppressed the device efficiency. Generally, R_ct2_ is inversely proportional to the electron density, which implies that reduced charge transport resistance increases the electron density of TiO_2_ photo-anode in a DSSC. This agrees with the small electron density available for transport in the device with catechol only, as the interfacial transport of electrons is hindered.

On the other hand, a substantial decrease in the charge transport resistance (R_ct2_) of the Z907 + catechol system (282.3 Ω) compared to that of the Z907 counterpart (532.1 Ω) is observed. Enhanced coverage of the small (catechol) and bulky (Z907) molecules on TiO_2_ film results in the stronger diffusion of charges between the layers, because the intermolecular distances between the sensitizers have been reduced by co-sensitization. Adsorption of only Z907 molecules with longer intermolecular distances creates vacant sites which serve as traps for the electrons after injection. This is responsible for the increased recombination with electrolyte in the Z907-based DSSC. The low Voc of the catechol-related devices is because of the usual downshift in the TiO_2_ conduction band after complexation. Also, Figure 5B represents the Bode-phase plot, which supports the existence of two semicircles owing to the presence of two major phase angle shifts in the plot. This plot gives information on the electron relaxation lifetime ($\tau_{e}$). The $\tau_{e}$ can be expressed using the following relation,

$$\tau_{e}=\frac{1}{2\pi f_{\max}}.$$ (1)

Here, $f_{max}$ represents the peak frequency maximum of the semicircle curve in the Bode phase plots. As seen from the plots, the peak maximum of Z907 and Z907 + catechol devices are nearly same, $\tau_{e}$ of 0.20 and 0.16 ms, respectively. But the catechol-based device shows an appreciable difference in its peak position ($\tau_{e}$ of 1.00 ms). Here, the relaxation lifetime is defined as the lifetime of relaxed electrons in the excited state of the sensitizer. A longer lifetime implies high possibility of electrons recombining with the ground state of the sensitizer. A shorter lifetime of the electrons in this state tends to increase the electrons injected into the TiO_2_ for transfer through the external circuit. We realized that the catechol-DSSC has the longest electron relaxation lifetime ($\tau_{e}$ of 1.00 ms), implying that most of the electrons spend their time in the ground state of the sensitizer and there is scarce promotion of electrons into the TiO_2_ conduction band. This accounts for the very low electron density obtained in that device. On the other hand, the co-sensitized DSSC has a little shorter timescale, $\tau_{e}$ of 0.16 ms, than the Z907-DSSC (0.20 ms) because the film has more coverage, which ensures that excited electrons find it easier to be injected into the TiO_2_ conduction band as the dye regeneration becomes much preferred. The Z907-DSSC has a comparably longer timescale for electron relaxation, because there are vacancies around the limited number of monolayers which served as traps, implying that the preference for efficient dye regeneration is lower and less driving force for injection of excited electrons into the TiO_2_ conduction band. Nonetheless, both the Z907-DSSC and co-sensitized DSSC have very high electron density which is a consequence of the vast difference between their timescale, $\tau_{e}$ of 0.20 and 0.16 ms, compared to that of the catechol-DSSC, $\tau_{e}$ of 1.00 ms. Details of other electrochemical data are given as ESI (Supplementary Table S1).

## Conclusion

In summary, two sensitizers, catechol and Z907, were employed for co-sensitization of DSSCs. Catechol acts as a small molecule with the ability to penetrate into vacant sites on a sensitized TiO_2_ film, and Z907 acts as the bulky molecule which creates spaces on adsorbing onto the TiO_2_ film. Z907 demonstrated that it did not give full coverage on the TiO_2_ film, but addition of catechol improved the coverage of active sites on the TiO_2_ surface which is evidenced by the IPCE spectra. The co-sensitized films, both in organic and aqueous electrolytes, show improved PCEs (%) of 5.4 and 0.80% compared to the Z907 sensitized films with only 5.08 and 0.50%, respectively. These represent 7.2 and 60% improvement in overall PCEs (%) of the devices with respect to their control devices. This work demonstrates for the first time ever the successful co-sensitization of DSSCs using both the Type-I and Type-II mechanisms and also gives a new direction for co-sensitization by employing bulky and small molecules.

## Data Availability

The original contributions presented in the study are included in the article/Supplementary Material; further inquiries can be directed to the corresponding author.
