# Supplementary material for: Preliminary Investigation on Vacancy Filling by Small Molecules on the Performance of Dye-Sensitized Solar Cells: The Case of a Type-II Absorber
Source: Front Chem. 2021 Jul 8;9:701781. doi: 10.3389/fchem.2021.701781 (PMC8297438; doi:10.3389/fchem.2021.701781)
Supplement: Supplementary file 1 [file DataSheet1.docx]

**Electronic Supplementary Information**

**Preliminary Investigation On Vacancy Filling By Small Molecules On The Performance Of Dye-Sensitized Solar Cells: The Case Of A Type-II Absorber**

*Francis Kwaku Asiam, Nguyen Huy Hao, Ashok Kumar Kaliamurthy, Hyeong Cheol Kang, Kicheon Yoo and Jae-Joon Lee**

*Research Center for Photoenergy harvesting & Conversion Technology (phct),*

*Department of Energy and Materials Engineering, Dongguk University, 04620, Seoul, Republic of Korea*

** Corresponding author:* [*jjlee@dongguk.edu*](mailto:jjlee@dongguk.edu)

**Table S1.** Kinetic, electrochemical and photovoltaic data summary of five devices each with organic solvent-based electrolyte.

| Sensitizer | Cell Area(cm^2^) | n_s_ x10^21^  ^b^(electrons/m^2^) | ^c^τ (ms) | Cµ (µF ) | χ (x 10^-3^) | J_sc_(mA/cm^-2^) | V_oc_(V) | FF(%) | η(%) |
| --- | --- | --- | --- | --- | --- | --- | --- | --- | --- |
| Catechol(0.1)^a^ | 0.31±0.02 | 0.79 | 1.00 | 4.95 | 3.84 | 0.77±0.04 | 0.47±0.01 | 68.95±1.07 | 0.25±0.01 |
| Z907(0.3)^a^+ Catechol(0.1)^a^ | 0.28±0.02 |  |  |  |  | 7.70±1.04 | 0.62±0.03 | 48.35±5.67 | 2.29±0.07 |
| Z907(0.3)^a^+ Catechol(0.075)^a^ | 0.29±0.02 |  |  |  |  | 8.00±0.29 | 0.64±0.01 | 61.56±2.02 | 3.14±0.20 |
| Z907(0.3)^a^+ Catechol(0.05)^a^ | 0.29±0.01 | 4.70 | 0.16 | 9.25 | 4.17 | 10.95±1.28 | 0.69±0.03 | 68.50±3.24 | 5.21±0.61 |
| Z907(0.3)^a^+ Catechol(0.025)^a^ | 0.30±0.01 |  |  |  |  | 8.72±0.91 | 0.64±0.01 | 61.82±4.05 | 3.45±0.19 |
| Z907(0.3)^a^ | 0.29±0.03 | 2.50 | 0.20 | 8.74 | 3.47 | 9.90±0.89 | 0.71±0.01 | 69.93±4.19 | 4.86±0.77 |

**^a^** numbers in parenthesis are concentrations (mM) of the sensitizers in ethanol solvent, **^b^** electron density in the TiO_2_ photoanode during device operation, **^c^** lifetime of electrons in the photoanode

Electron density was calculated from the relation;


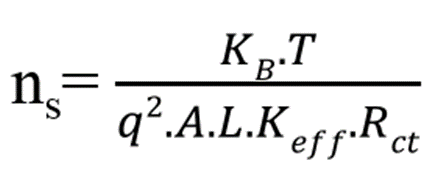


Where K_B_=8.617×10^−5^ (eV.K^−1^) Boltzmann constant

T= 298.15 (K) absolute temperature

q = electron charge 1.602×10^−19^ C

A= electrode area

L=TiO_2_ layer thickness

K_eff_= peak frequency related with R_ct_


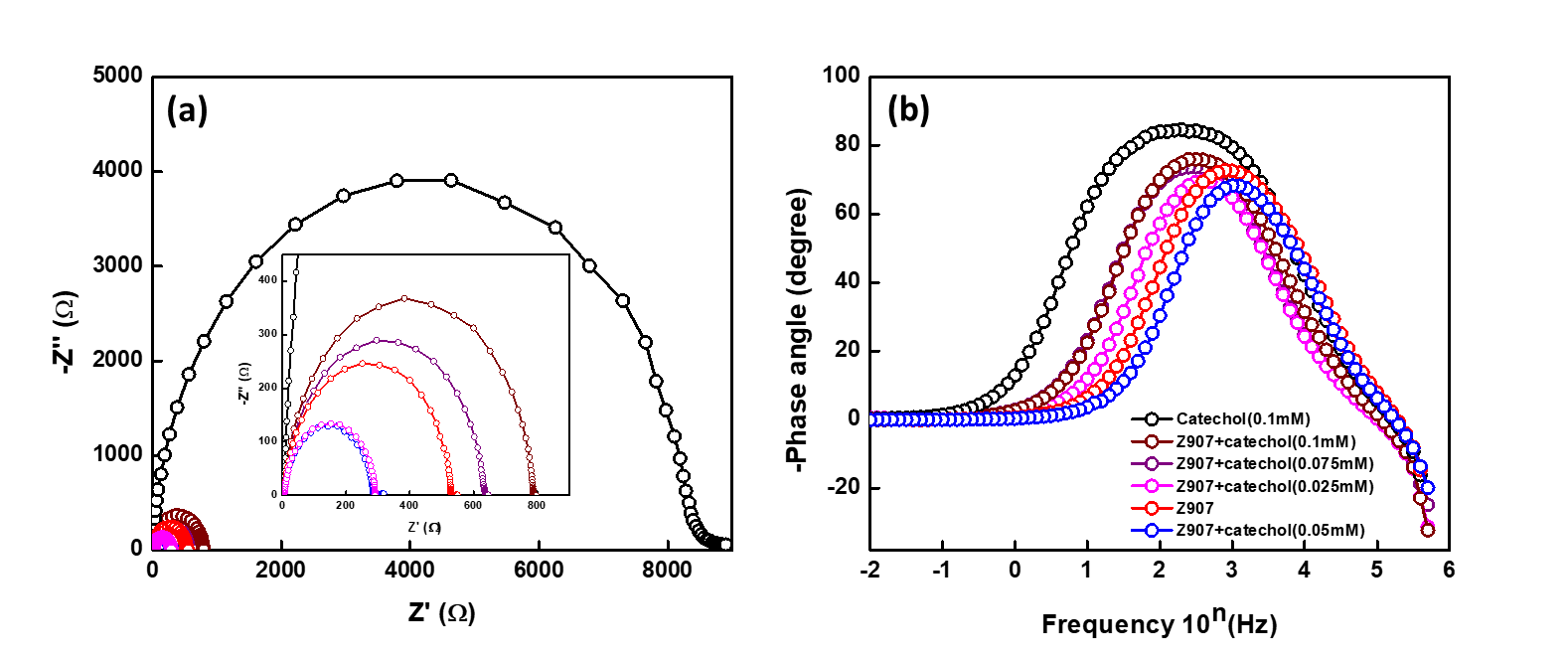


**Figure S1.** Electrochemical impedance plots (a) Nyquist and (b) Bode-Phase, of devices tested for various optimization conditions with organic solvent-based electrolyte.


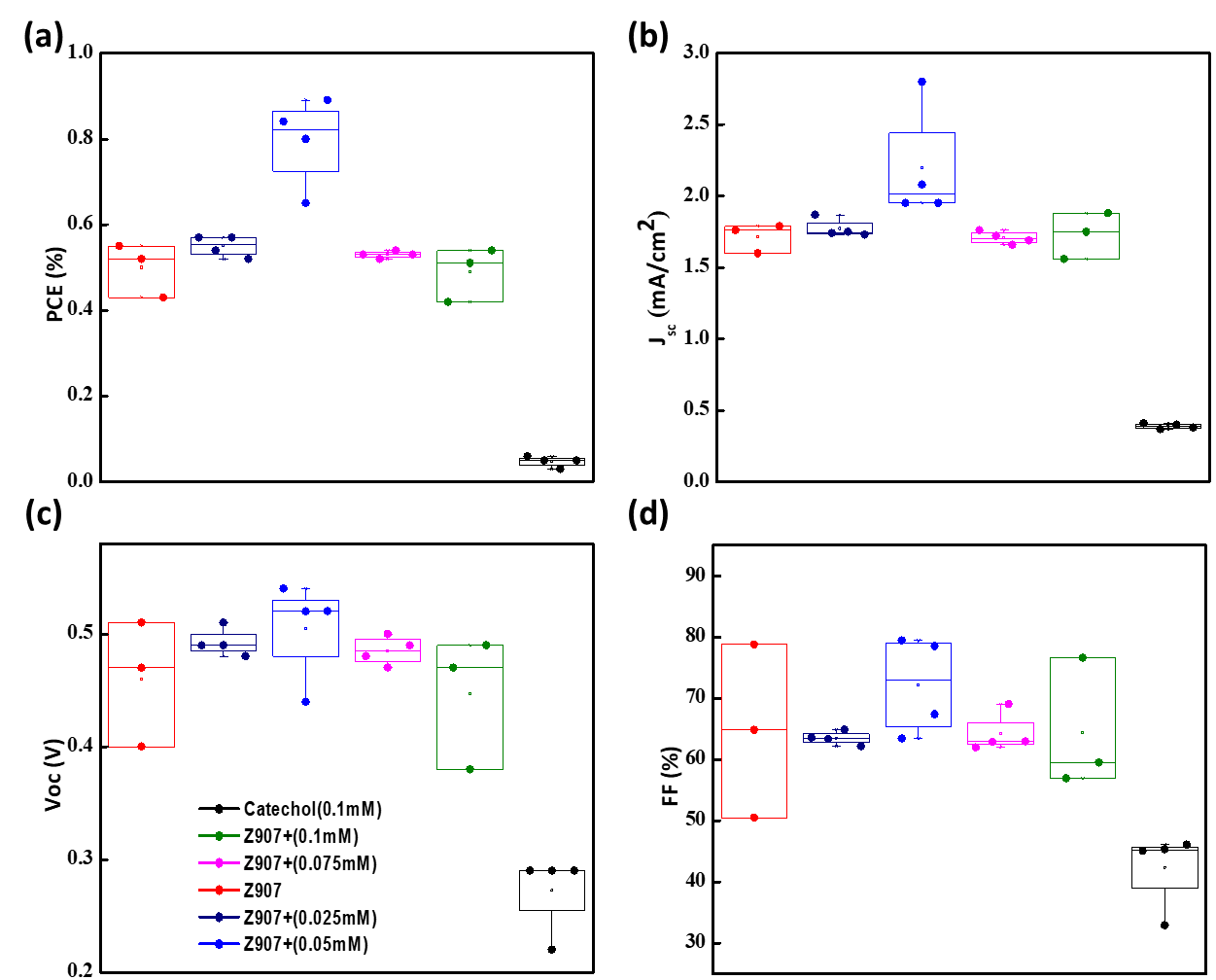


**Figure S2.** Distribution of photovoltaic data, for different optimization conditions of the catechol sensitizer (0-0.1mM) with Z907 (0.3mM) employing aqueous electrolyte.

**Table S2.** Summary of photovoltaic, desorption and optimization parameters of the devices with aqueous electrolyte.

| Sensitizer | | Cell Area (cm^2^) | Adsorbed molecules(x10^17^) | | | ^a^ (mg/cm^2^) | J_sc_ (mA/cm^-2^) | V_oc_ (V) | FF (%) | $\boldsymbol{\eta(\%)}$ |
| --- | --- | --- | --- | --- | --- | --- | --- | --- | --- | --- |
| Z907(mM) | **Catechol(mM)** |  | **Catechol** | **Z907** | **Total** |  |  |  |  |  |
| 0.3 | 0 | 0.30±0.02 | 0 | 1.01 | 1.01 |  | 1.72±0.10 | 0.46±0.06 | 64.70±14.06 | 0.50±0.07 |
|  | 0.025 | 0.29±0.02 | 3.68 | 0.98 | 4.66 | 1.42 | 1.77±0.07 | 0.49±0.01 | 63.50±1.11 | 0.55±0.03 |
|  | 0.050 | 0.30±0.02 | 6.39 | 0.98 | 7.37 | 1.50 | 2.20±0.41 | 0.51±0.04 | 72.20±8.01 | 0.80±0.15 |
|  | 0.075 | 0.29±0.01 | 9.18 | 0.81 | 9.99 | 1.66 | 1.71±0.04 | 0.49±0.01 | 64.23±3.25 | 0.53±0.01 |
|  | 0.1 | 0.28±0.03 | 12.22 | 0.66 | 12.87 | 1.90 | 1.73±0.16 | 0.45±0.06 | 64.38±10.68 | 0.49±0.07 |
| 0 | 0.1 | 0.30±0.01 | 19.81 | 0 | 19.81 |  | 0.39±0.02 | 0.27±0.04 | 42.38±6.32 | 0.05±0.02 |

**^a^** desorbed amount of Z907 from TiO_2_ film surface after co-sensitization with catechol


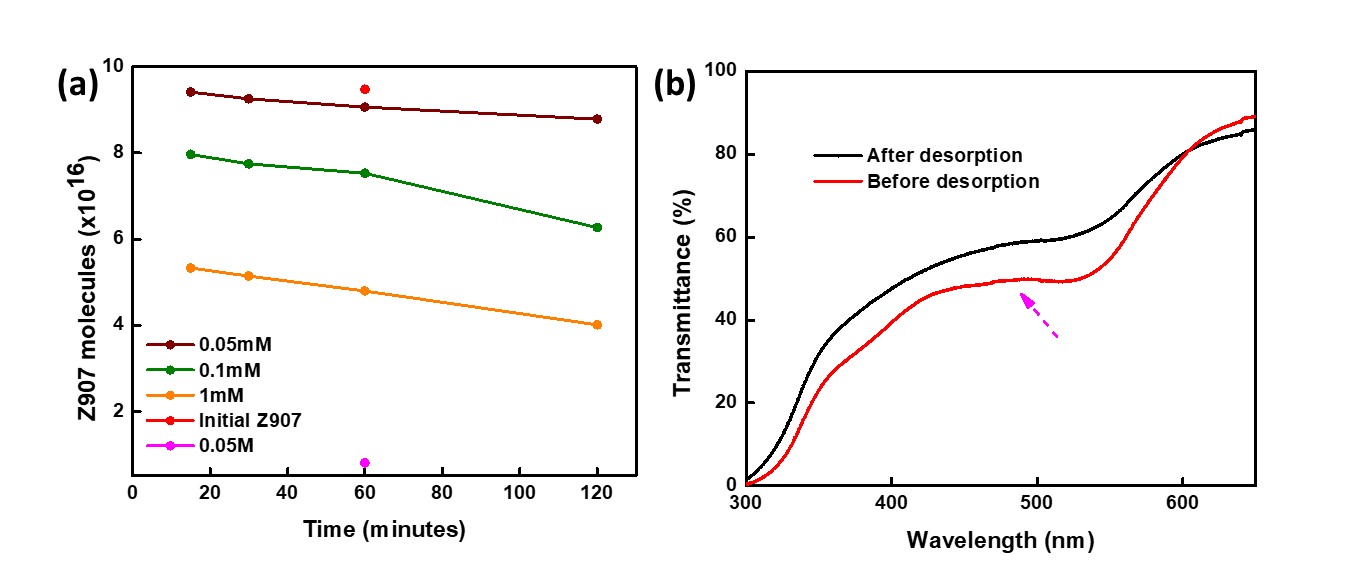


**Figure S3.** (a) Kinetic data for desorption studies of Z907 molecules and (b) Transmittance spectra, after immersing films in catechol solutions.


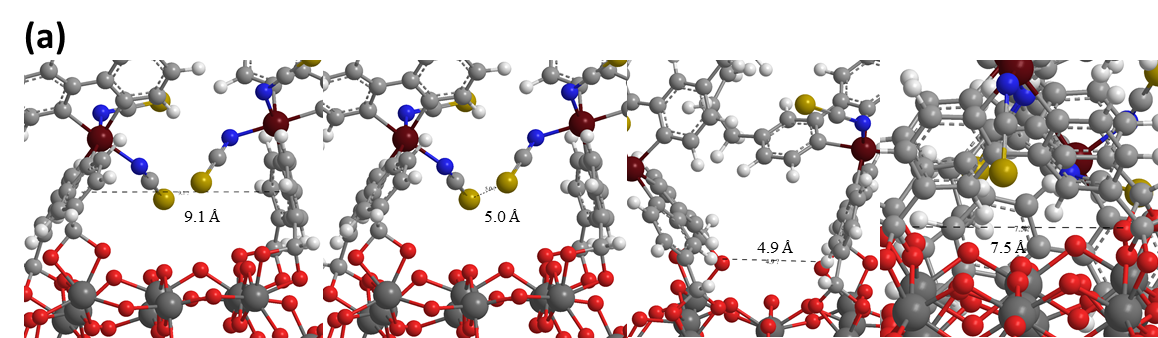


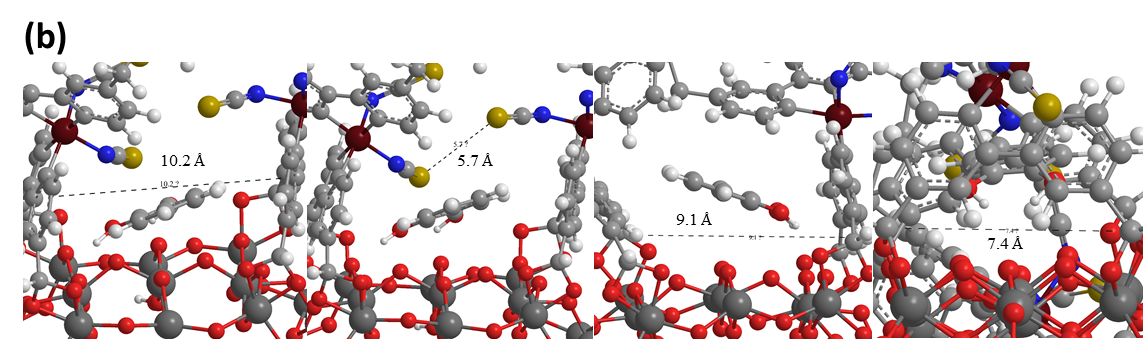


**Figure S4.** Molecular mechanics (MM2) force field visualizations of the (a) vacancy creation by three Z907 molecules and (b) filling by catechol, on (TiO_2_)_24_ surface with two different alignment phases, first two and last two.

**Table S3.** Summary of the atom distances related to the vacancy creation and filling sites, on (TiO_2_)_24_ and the total energy of the frame computed with MM2 force field at RMS Gradient of 0.0001 and RMS Move of 0.0001.

| Atoms | Distance with Z907 only (Å) | Distance with Z907+Catechol (Å) | Change in Distance (Å) |
| --- | --- | --- | --- |
| C(103) – C(105) | 7.4888 | 7.4442 | -0.0446 |
| Ti(48) – Ti(54) | 6.3905 | 6.3472 | -0.0433 |
| S(240) – S(347) | 4.9958 | 5.7417 | 0.7459 |
| C(197) – C(304) | 9.1098 | 10.2453 | 1.1355 |
| H(144) – H(247) | 4.8586 | 9.0903 | 4.2317 |
| Energy of frame (kcal/mol) | **661.441** | **346.256** | **-315.185^a^** |

**^a^** is the stabilization induced energy change when two catechol molecules migrate to the surface of (TiO_2_)_24_


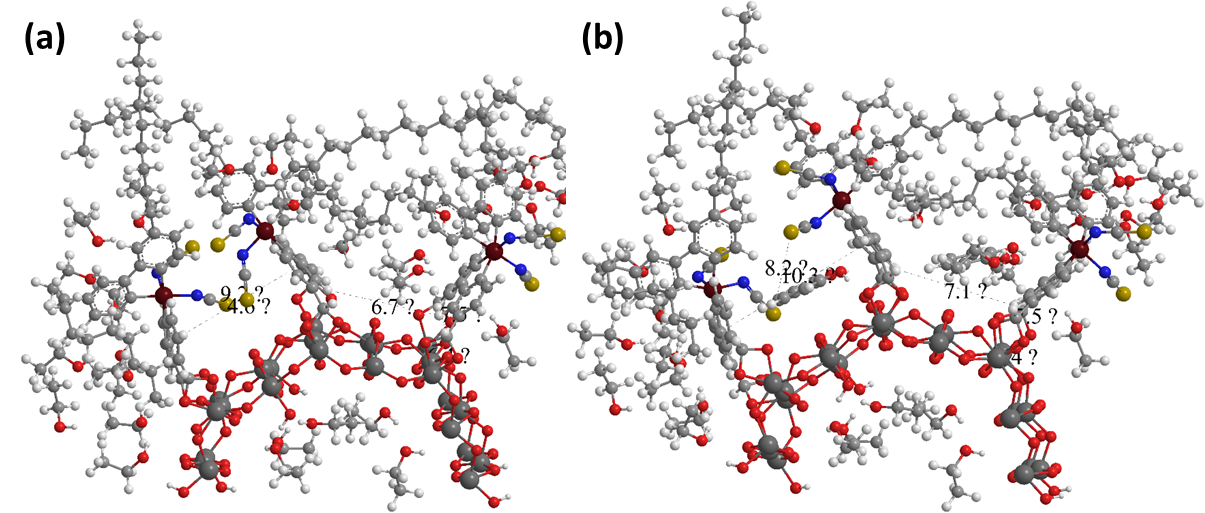


**Figure S5.** MM2 force field prediction of the (a) sterically induced vacancy as a result of bulky side chains on Z907 when adsorbed on the TiO_2_ sites and (b) the filling of those sites by two small catechol sensitizers in the presence of thirty discrete molecules of ethanol solvent.

**Table S4.** Summary of the atom distances related to the vacancy creation and filling sites, on (TiO_2_)_24_ and the total energy of the frame computed with MM2 force field at RMS Gradient of 0.0001 and RMS Move of 0.0001 in discrete solvent environment for comparison.

| Atoms | Distance with Z907 only (Å) | Distance with Z907+Catechol (Å) | Change in Distance (Å) |
| --- | --- | --- | --- |
| C(103) – C(105) | 7.4605 | 7.4551 | -0.0054 |
| Ti(48) – Ti(54) | 6.3784 | 6.3811 | 0.0027 |
| S(240) – S(347) | 4.5933 | 8.1967 | 3.6034 |
| C(197) – C(304) | 9.1618 | 10.2730 | 1.1112 |
| H(144) – H(247) | 6.6706 | 7.0527 | 0.3821 |
| Energy of frame (kcal/mol) | **347.181** | **280.865** | **-66.316^a^** |

**^a^** is the stabilization induced energy change when two catechol molecules migrate to the surface of (TiO_2_)_24_

**Table S5.** Summary of some co-sensitization based research works and their respective mechanisms for PCE (%) enhancement.

| Dye Structure | Code | J_SC_  (mA/cm^2^) | V_oc_  (V) | FF | PCE  (%) | ^a^ PCE  (%) | | Ref. | |
| --- | --- | --- | --- | --- | --- | --- | --- | --- | --- |
| 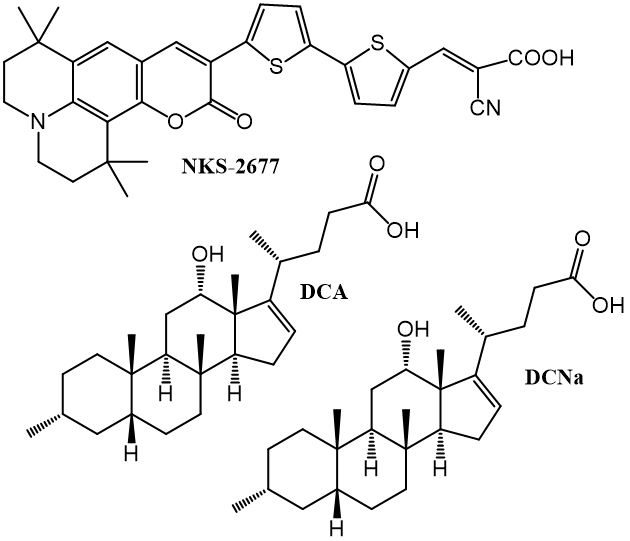 | Deoxycholic acid (DCA), DCNa, and NKX-2677 | 13.98 | 0.64 | 0.76 | 6.70 | 4.10 | (Ren et al., 2010) | |  |
| 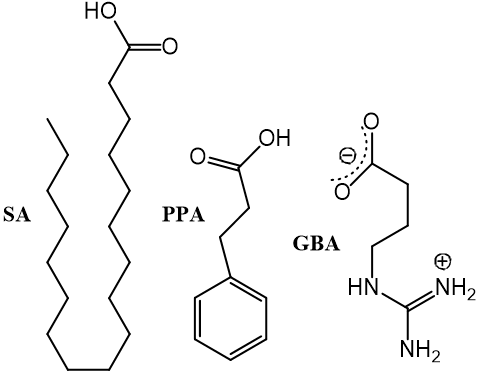 | N719, Stearic acid, GBA, PPA | 14.20 | 0.78 | 0.66 | 7.40 | 5.90 | (Lim et al., 2011) | |  |
| 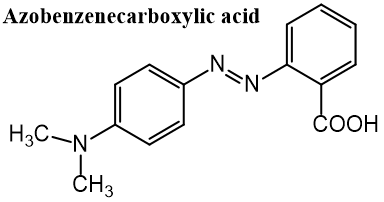 | Azobenzenecarboxylic acid, N719 | 14.87 | 0.77 |  | 5.20 | 4.30 | (Mazloum-Ardakani and Arazi, 2019) | |  |
| 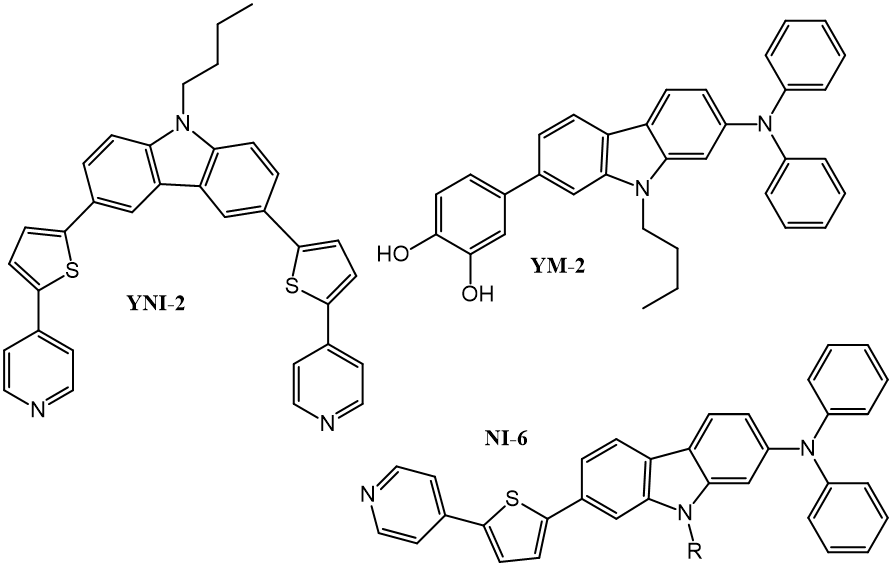 | YM-2, NI-6, YNI-2 | 1.61 | 0.49 | 0.55 | 0.43 | 2.02 | (Ooyama et al., 2015) | |  |
| 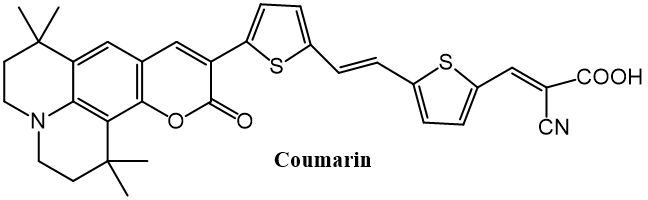 | N719, Coumarin, QD(CdSe) | 21.00 | 0.71 | 0.67 | 9.90 | 8.20 | (Elangovan and Venkatachalam, 2015) | |  |
| 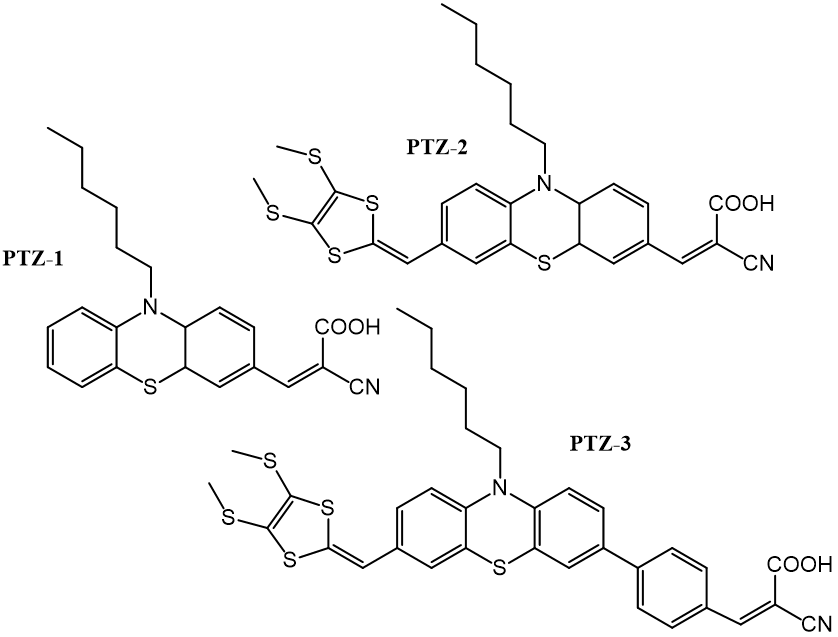 | PTZ-1, PTZ-2, PTZ-3, N719 | 16.37 | 0.67 | 0.74 | 8.12 | 6.97 | (Luo et al., 2016) | |  |
| 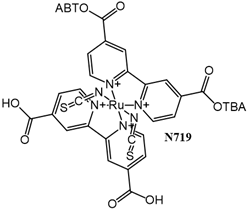 | Bi_2_S_3_ QD, N719 | 16.77 | 0.70 | 0.64 | 7.50 | 6.64 | (Sun et al., 2017) | |  |
| 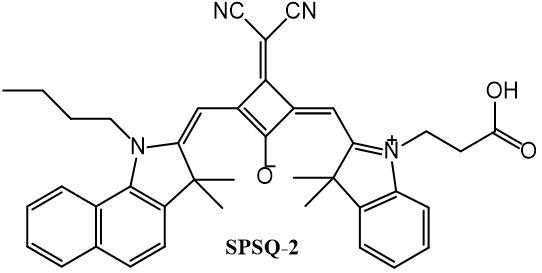 | SPSQ-2, N719 with upconverter | 22.16 | 0.66 | 0.72 | 10.53 | 5.03 | (Ambapuram et al., 2020) | |  |
| 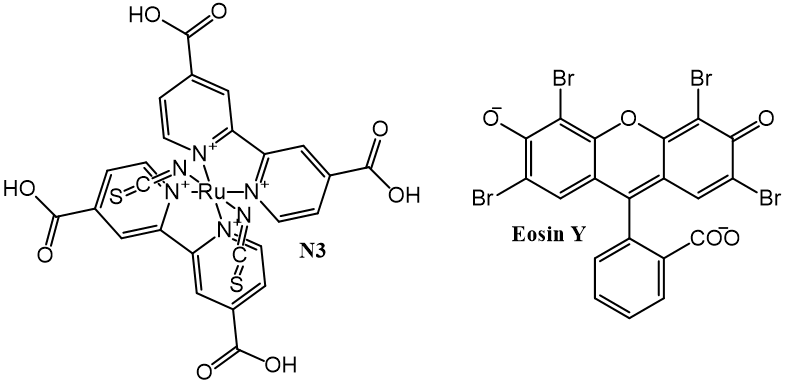 | Eosin-Y, N3, N719 | 16.27 | 0.59 | 0.72 | 6.90 | 5.69 | (Kumar et al., 2019) | |  |
| 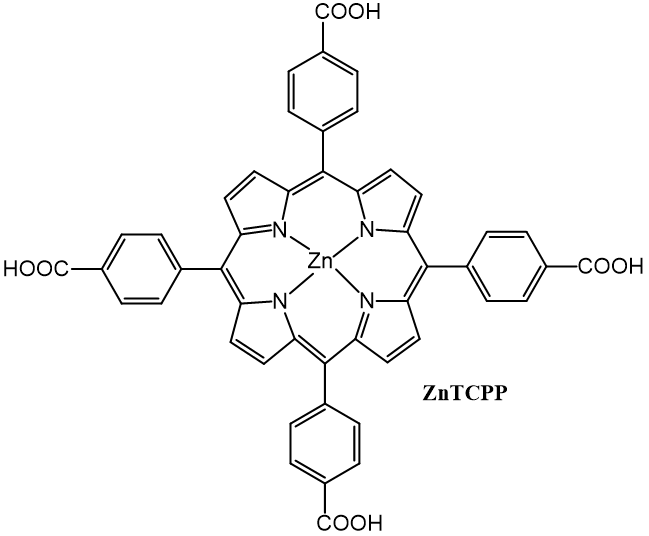 | ZnTCPP, N719 | 18.90 | 0.61 | 0.55 | 6.35 | 4.74 | (Dehghani, 2013) | |  |
| 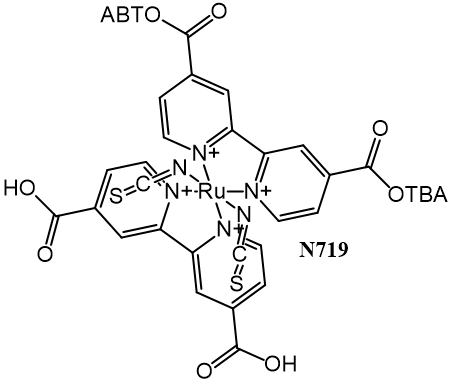 | CdS QD, N719 | 11.73 | 0.74 | 0.64 | 5.57 | 5.26 | (Li et al., 2013) | |  |
| 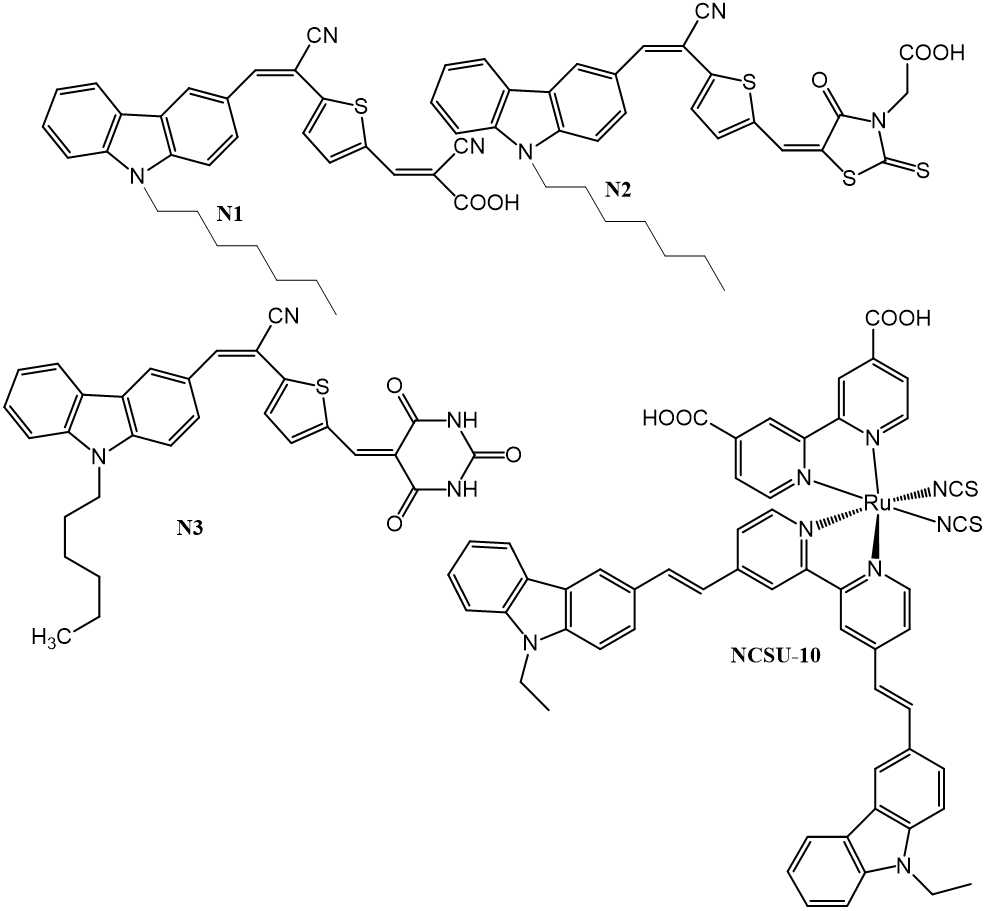 | NCSU-10, N1, N2, N3 | 19.87 | 0.66 | 0.67 | 8.73 | 8.25 | (Naik et al., 2017) | |  |
| 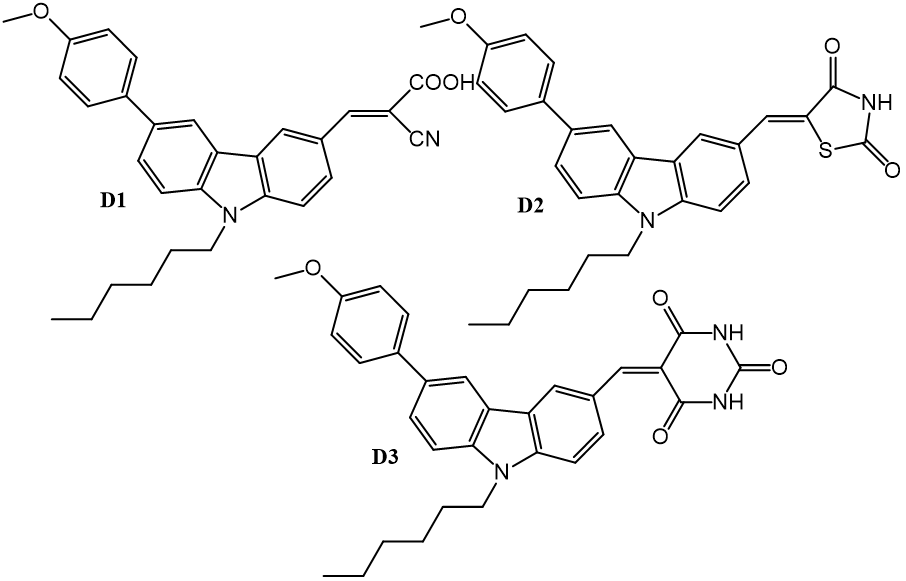 | NCSU-10, D1,D2,D3 | 19.25 | 0.68 | 0.64 | 8.32 | 8.25 | (Naik et al., 2018) | |  |
| 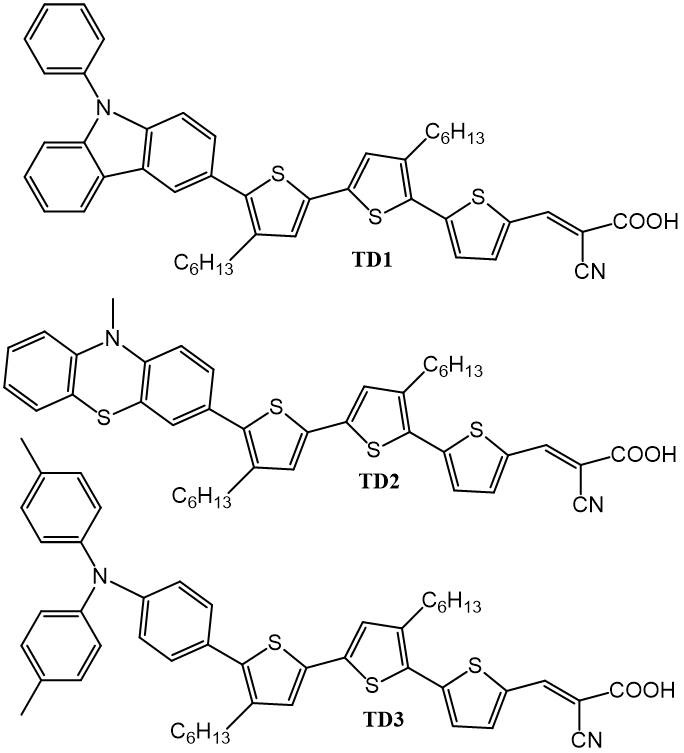 | TD1, TD2, TD3, N719 | 16.90 | 0.71 | 0.68 | 8.02 | 7.29 | (Wu et al., 2020) | |  |
| 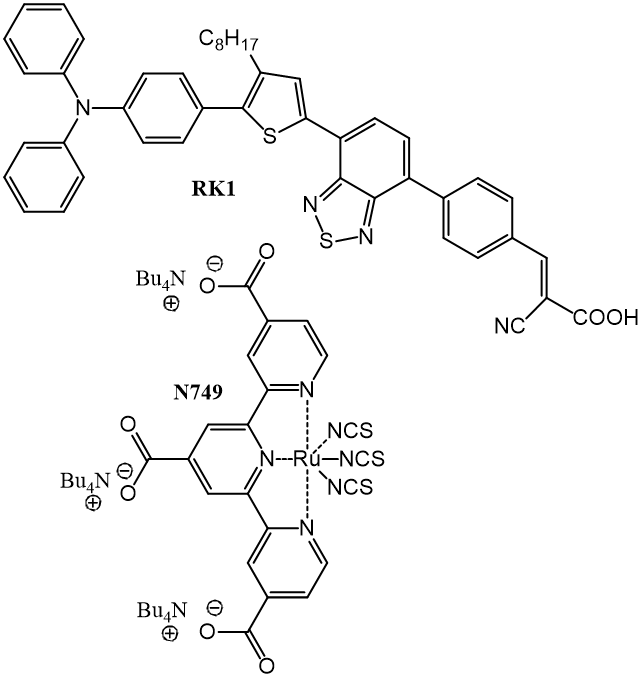 | N749, RK1 | 19.45 | 0.69 | 0.61 | 8.15 | 5.76 | (Younas and Harrabi, 2020) | |  |
| 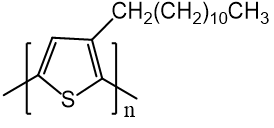 | P3DDT, N719 | 7.51 | 0.74 | 0.56 | 3.25 | 2.33 | (Zhang et al., 2011) | |  |
| 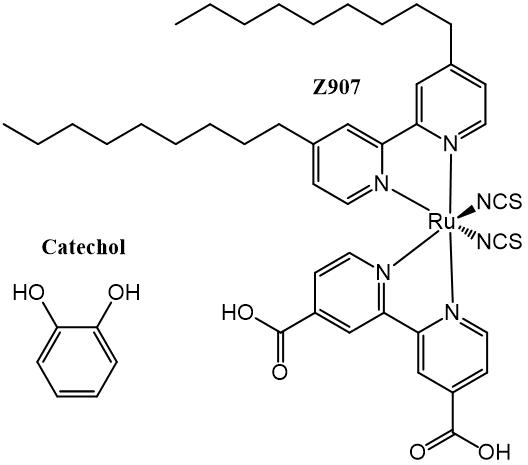 | **Catechol, Z907** | **12.09** | **0.67** | **0.68** | **5.44** | **5.08** | **This work** | |  |

**^a^** The PCE(%) of the control device

**Table S6**. Cartesian coordinates of the two different MM2 simulation grids.

| **(Z907)_3_-(TiO_2_)_24_** | | | | | | | | **[(Z907)_3_+(Catechol)_2_]-(TiO_2_)_24_** | | | | | | | |
| --- | --- | --- | --- | --- | --- | --- | --- | --- | --- | --- | --- | --- | --- | --- | --- |
| Ti(1) | 10.875 | 0.8 | 6.92 | C(220) | -3.497 | 10.411 | -4.848 | Ti(1) | 10.256 | 0.319 | 6.417 | C(220) | -4.188 | 9.467 | -5.342 |
| Ti(2) | 12.444 | -0.621 | 0.983 | C(221) | -4.695 | 10.284 | -3.894 | Ti(2) | 11.387 | -1.666 | 0.673 | C(221) | -5.273 | 8.603 | -4.689 |
| Ti(3) | 11.808 | 0.357 | 3.929 | C(222) | -5.955 | 10.93 | -4.49 | Ti(3) | 11.125 | -0.119 | 3.41 | C(222) | -6.612 | 8.676 | -5.437 |
| Ti(4) | 8.474 | 2.942 | 5.633 | C(223) | -7.173 | 10.825 | -3.558 | Ti(4) | 8.171 | 2.809 | 5.21 | C(223) | -7.784 | 8.089 | -4.631 |
| Ti(5) | 9.33 | 2.291 | 2.637 | C(224) | -8.34 | 11.68 | -4.077 | Ti(5) | 8.87 | 2.117 | 2.157 | C(224) | -8.17 | 8.947 | -3.413 |
| Ti(6) | 10.514 | 1.944 | -0.35 | C(225) | -9.582 | 11.594 | -3.176 | Ti(6) | 9.99 | 1.246 | -0.703 | C(225) | -9.546 | 8.573 | -2.84 |
| Ti(7) | 5.193 | 2.869 | 4.395 | C(226) | -10.71 | 12.505 | -3.685 | Ti(7) | 4.82 | 2.9 | 4.148 | C(226) | -9.979 | 9.53 | -1.718 |
| Ti(8) | 6.26 | 2.64 | 1.374 | C(227) | -12.006 | 12.331 | -2.879 | Ti(8) | 5.771 | 2.555 | 1.102 | C(227) | -11.43 | 9.287 | -1.275 |
| Ti(9) | 7.209 | 1.238 | -1.352 | C(228) | -13.095 | 13.314 | -3.327 | Ti(9) | 6.609 | 1.252 | -1.71 | C(228) | -11.904 | 10.35 | -0.275 |
| Ti(10) | 1.901 | 3.174 | 3.222 | C(229) | 0.365 | 14.492 | -0.235 | Ti(10) | 1.523 | 3.481 | 3.065 | C(229) | -2.39 | 14.442 | -0.287 |
| Ti(11) | 3.1 | 2.299 | 0.378 | C(230) | -0.848 | 14.408 | 0.707 | Ti(11) | 2.597 | 2.495 | 0.212 | C(230) | -3.236 | 13.978 | 0.909 |
| Ti(12) | 3.917 | 1.27 | -2.536 | C(231) | -1.668 | 15.708 | 0.72 | Ti(12) | 3.358 | 1.882 | -2.868 | C(231) | -4.321 | 15.002 | 1.274 |
| Ti(13) | -0.12 | 0.359 | 3.252 | C(232) | -2.944 | 15.579 | 1.57 | Ti(13) | -1.199 | 1.37 | 2.837 | C(232) | -5.194 | 14.53 | 2.448 |
| Ti(14) | 0.404 | 0.146 | 0.167 | C(233) | -3.929 | 16.727 | 1.293 | Ti(14) | -0.39 | 0.926 | -0.218 | C(233) | -6.306 | 15.541 | 2.77 |
| Ti(15) | 1.683 | -1.381 | -2.276 | C(234) | -5.266 | 16.535 | 2.026 | Ti(15) | 0.355 | 0.228 | -3.238 | C(234) | -7.225 | 15.053 | 3.902 |
| O(16) | 6.525 | 2.721 | 5.841 | C(235) | -6.285 | 17.62 | 1.638 | O(16) | 6.227 | 2.905 | 5.53 | C(235) | -8.364 | 16.05 | 4.172 |
| O(17) | 3.364 | 3.599 | 4.475 | C(236) | -7.646 | 17.401 | 2.315 | O(17) | 2.987 | 3.586 | 4.385 | C(236) | -9.337 | 15.538 | 5.245 |
| O(18) | 3.244 | -0.509 | -3.053 | C(237) | -8.668 | 18.467 | 1.898 | O(18) | 2.306 | 0.278 | -3.329 | C(237) | -10.457 | 16.548 | 5.53 |
| O(19) | 12.241 | 1.144 | 0.164 | N(238) | 3.562 | 8.492 | -1.24 | O(19) | 11.12 | -0.366 | -0.762 | N(238) | 2.855 | 10.114 | -1.269 |
| O(20) | 9.169 | 1.028 | -1.469 | C(239) | 4.338 | 7.543 | -1.484 | O(20) | 8.55 | 1.305 | -2.052 | C(239) | 3.975 | 9.976 | -1.803 |
| O(21) | 5.87 | 1.182 | -2.796 | S(240) | 5.312 | 6.377 | -1.746 | O(21) | 5.159 | 1.096 | -3.037 | S(240) | 5.359 | 9.816 | -2.46 |
| O(22) | 11.133 | 2.129 | 3.409 | N(241) | 2.962 | 9.655 | -3.597 | O(22) | 10.682 | 1.701 | 2.803 | N(241) | 1.942 | 10.845 | -3.68 |
| O(23) | 8.046 | 3.408 | 1.644 | C(242) | 2.455 | 9.978 | -4.693 | O(23) | 7.501 | 3.382 | 1.521 | C(242) | 1.223 | 11.346 | -4.57 |
| O(24) | 4.654 | 3.483 | 0.623 | S(243) | 1.851 | 10.372 | -6.054 | O(24) | 4.299 | 3.483 | 0.196 | S(243) | 0.367 | 11.984 | -5.681 |
| O(25) | 1.232 | 1.915 | -0.112 | H(244) | 3.276 | 10.947 | -0.327 | O(25) | 0.675 | 2.579 | -0.196 | H(244) | 1.603 | 12.203 | -0.266 |
| O(26) | 13.123 | 0.493 | 2.473 | H(245) | 2.623 | 13.125 | 0.399 | O(26) | 12.224 | -0.264 | 1.785 | H(245) | 0.152 | 13.957 | 0.459 |
| O(27) | 11.342 | -0.973 | 2.559 | H(246) | -0.909 | 12.895 | -1.853 | O(27) | 10.316 | -1.524 | 2.299 | H(246) | -2.936 | 12.535 | -1.999 |
| O(28) | 12.221 | 1.31 | 5.598 | H(247) | 1.138 | 3.864 | -2.002 | O(28) | 11.637 | 0.742 | 5.101 | H(247) | 1.37 | 4.858 | -2.118 |
| O(29) | 10.578 | -0.312 | 5.311 | H(248) | 3.345 | 5.086 | -5.375 | O(29) | 9.814 | -0.675 | 4.767 | H(248) | 3.523 | 6.433 | -5.372 |
| O(30) | 8.802 | 3.73 | 3.865 | H(249) | 3.006 | 7.364 | -4.805 | O(30) | 8.807 | 3.545 | 3.502 | H(249) | 2.912 | 8.628 | -4.698 |
| O(31) | 9.538 | 1.066 | 1.118 | H(250) | -2.253 | 8.026 | -5.08 | O(31) | 8.885 | 0.677 | 0.821 | H(250) | -2.18 | 7.701 | -5.468 |
| O(32) | 6.607 | 0.916 | 0.493 | H(251) | -0.219 | 7.322 | -4.089 | O(32) | 5.96 | 0.868 | 0.107 | H(251) | -0.134 | 7.694 | -4.293 |
| O(33) | 5.819 | 3.888 | 2.836 | H(252) | -1.976 | 11.763 | -3.208 | O(33) | 5.297 | 3.861 | 2.502 | H(252) | -3.415 | 11.18 | -3.485 |
| O(34) | 2.446 | 3.815 | 1.444 | H(253) | 1.018 | 9.258 | 0.752 | O(34) | 2.293 | 4.066 | 1.354 | H(253) | 0.31 | 9.979 | 0.791 |
| O(35) | 3.815 | 0.775 | -0.635 | H(254) | 0.588 | 7.77 | 2.559 | O(35) | 3.174 | 1.116 | -1.063 | H(254) | 0.098 | 8.391 | 2.539 |
| O(36) | 1.463 | -1.44 | -0.338 | H(255) | 0.467 | 4.508 | -0.097 | O(36) | 0.557 | -0.368 | -1.367 | H(255) | 0.829 | 5.271 | -0.17 |
| O(37) | -0.532 | 1.214 | 1.537 | H(256) | 2.331 | 2.712 | -5.18 | O(37) | -1.495 | 1.999 | 1.013 | H(256) | 3.209 | 4.11 | -5.357 |
| O(38) | 0.828 | -0.685 | 1.905 | H(257) | -0.544 | 5.546 | 3.2 | O(38) | -0.111 | 0.262 | 1.618 | H(257) | -0.446 | 6.169 | 3.219 |
| O(39) | 3.551 | 2.867 | -1.449 | H(258) | -3.377 | 11.472 | -5.172 | O(39) | 2.672 | 3.221 | -1.607 | H(258) | -4.551 | 10.515 | -5.452 |
| O(40) | 2.807 | 1.734 | 2.235 | H(259) | -3.694 | 9.832 | -5.779 | O(40) | 2.249 | 1.948 | 2.065 | H(259) | -3.99 | 9.103 | -6.377 |
| O(41) | 5.701 | 1.621 | 2.961 | H(260) | -4.882 | 9.212 | -3.652 | O(41) | 5.192 | 1.598 | 2.72 | H(260) | -4.937 | 7.541 | -4.639 |
| O(42) | 6.818 | 2.96 | -0.491 | H(261) | -4.457 | 10.803 | -2.938 | O(42) | 6.501 | 2.927 | -0.693 | H(261) | -5.415 | 8.958 | -3.646 |
| O(43) | 10.435 | 3.103 | 1.237 | H(262) | -5.74 | 12.007 | -4.689 | O(43) | 9.533 | 2.784 | 0.432 | H(262) | -6.849 | 9.735 | -5.694 |
| O(44) | 8.459 | 1.555 | 4.235 | H(263) | -6.196 | 10.451 | -5.469 | O(44) | 7.978 | 1.47 | 3.782 | H(263) | -6.508 | 8.129 | -6.404 |
| O(45) | 9.018 | 1.354 | 6.663 | H(264) | -7.484 | 9.758 | -3.463 | O(45) | 8.493 | 1.138 | 6.203 | H(264) | -8.665 | 8.012 | -5.312 |
| O(46) | 0.775 | 1.844 | 4.142 | H(265) | -6.895 | 11.179 | -2.539 | O(46) | 0.407 | 2.006 | 3.751 | H(265) | -7.533 | 7.054 | -4.301 |
| O(47) | 0.628 | 0.2 | -1.793 | H(266) | -8.014 | 12.746 | -4.14 | O(47) | -0.554 | 1.521 | -2.093 | H(266) | -7.405 | 8.845 | -2.609 |
| Ti(48) | -1.581 | -2.16 | -3.275 | H(267) | -8.607 | 11.349 | -5.108 | Ti(48) | -2.426 | -1.876 | -3.485 | H(267) | -8.192 | 10.02 | -3.718 |
| Ti(49) | -2.478 | -5.455 | -2.576 | H(268) | -9.936 | 10.536 | -3.137 | Ti(49) | -2.49 | -5.344 | -2.885 | H(268) | -10.302 | 8.615 | -3.66 |
| Ti(50) | -3.393 | -8.551 | -2.033 | H(269) | -9.309 | 11.898 | -2.139 | Ti(50) | -3.118 | -8.521 | -2.588 | H(269) | -9.523 | 7.528 | -2.453 |
| Ti(51) | -2.612 | -1.505 | -0.317 | H(270) | -10.375 | 13.568 | -3.625 | Ti(51) | -2.825 | -1.419 | -0.339 | H(270) | -9.297 | 9.401 | -0.846 |
| Ti(52) | -4.04 | -4.534 | 0.046 | H(271) | -10.919 | 12.28 | -4.758 | Ti(52) | -3.846 | -4.662 | -0.091 | H(271) | -9.882 | 10.584 | -2.071 |
| Ti(53) | -4.919 | -7.706 | 0.615 | H(272) | -12.379 | 11.284 | -2.988 | Ti(53) | -4.381 | -7.977 | 0.255 | H(272) | -12.106 | 9.308 | -2.163 |
| Ti(54) | -3.425 | -0.575 | 2.635 | H(273) | -11.797 | 12.493 | -1.798 | Ti(54) | -3.674 | -1.049 | 2.683 | H(273) | -11.514 | 8.271 | -0.824 |
| Ti(55) | -4.217 | -3.931 | 3.172 | H(274) | -14.025 | 13.179 | -2.729 | Ti(55) | -3.619 | -4.535 | 3.08 | H(274) | -12.932 | 10.126 | 0.092 |
| Ti(56) | -5.108 | -7.038 | 3.709 | H(275) | -12.761 | 14.369 | -3.199 | Ti(56) | -4.237 | -7.717 | 3.4 | H(275) | -11.233 | 10.403 | 0.61 |
| O(57) | 0.382 | -2.266 | -3.431 | H(276) | -13.355 | 13.163 | -4.4 | O(57) | -0.511 | -1.458 | -3.713 | H(276) | -11.923 | 11.361 | -0.741 |
| O(58) | -2.58 | -3.821 | -3.651 | H(277) | 1.192 | 15.049 | 0.261 | O(58) | -2.571 | -3.765 | -4.033 | H(277) | -1.801 | 15.347 | -0.012 |
| O(59) | -3.736 | -6.89 | -3.004 | H(278) | 0.099 | 15.084 | -1.139 | O(59) | -3.703 | -6.81 | -3.328 | H(278) | -3.052 | 14.751 | -1.13 |
| O(60) | -3.043 | -1.361 | -2.229 | H(279) | -1.524 | 13.594 | 0.361 | O(60) | -3.503 | -0.958 | -2.12 | H(279) | -3.722 | 13.005 | 0.662 |
| O(61) | -1.313 | -2.535 | -1.364 | H(280) | -0.518 | 14.135 | 1.737 | O(61) | -1.691 | -2.259 | -1.7 | H(280) | -2.572 | 13.797 | 1.788 |
| O(62) | -3.965 | -0.553 | 0.743 | H(281) | -1.044 | 16.554 | 1.09 | O(62) | -3.865 | -0.285 | 0.882 | H(281) | -3.841 | 15.975 | 1.533 |
| O(63) | -2.21 | -1.716 | 1.592 | H(282) | -1.962 | 15.944 | -0.331 | O(63) | -2.293 | -1.81 | 1.508 | H(282) | -4.973 | 15.176 | 0.385 |
| O(64) | -5.212 | -3.826 | 1.464 | H(283) | -3.45 | 14.613 | 1.335 | O(64) | -4.844 | -4.245 | 1.558 | H(283) | -5.652 | 13.546 | 2.194 |
| O(65) | -3.036 | -4.549 | 1.739 | H(284) | -2.677 | 15.551 | 2.652 | O(65) | -2.601 | -4.769 | 1.429 | H(284) | -4.556 | 14.377 | 3.351 |
| O(66) | -6.185 | -7.33 | 2.073 | H(285) | -3.467 | 17.697 | 1.591 | O(66) | -5.489 | -7.897 | 1.877 | H(285) | -5.85 | 16.517 | 3.056 |
| O(67) | -3.929 | -7.731 | 2.313 | H(286) | -4.13 | 16.778 | 0.196 | O(67) | -3.19 | -8.151 | 1.808 | H(286) | -6.919 | 15.718 | 1.855 |
| O(68) | -5.186 | -8.206 | -1.269 | H(287) | -5.686 | 15.533 | 1.772 | O(68) | -4.837 | -8.381 | -1.615 | H(287) | -7.654 | 14.061 | 3.63 |
| O(69) | -3.212 | -8.374 | -0.095 | H(288) | -5.094 | 16.553 | 3.128 | O(69) | -2.728 | -8.497 | -0.673 | H(288) | -6.626 | 14.909 | 4.832 |
| O(70) | -2.32 | -5.195 | -0.643 | H(289) | -5.885 | 18.623 | 1.919 | O(70) | -2.144 | -5.097 | -0.979 | H(289) | -7.93 | 17.025 | 4.497 |
| O(71) | -4.188 | -4.712 | -1.911 | H(290) | -6.426 | 17.615 | 0.531 | O(71) | -4.152 | -4.69 | -2.04 | H(290) | -8.928 | 16.236 | 3.228 |
| O(72) | -5.438 | -5.917 | -0.013 | H(291) | -8.035 | 16.392 | 2.041 | O(72) | -5.093 | -6.185 | -0.134 | H(291) | -9.787 | 14.576 | 4.904 |
| O(73) | -4.202 | -2.652 | -0.497 | H(292) | -7.522 | 17.419 | 3.424 | O(73) | -4.263 | -2.762 | -0.404 | H(292) | -8.779 | 15.331 | 6.189 |
| O(74) | -1.472 | 0.096 | -0.441 | H(293) | -9.655 | 18.288 | 2.383 | O(74) | -2.226 | 0.42 | -0.703 | H(293) | -11.137 | 16.177 | 6.33 |
| O(75) | -1.894 | -0.217 | 3.825 | H(294) | -8.326 | 19.486 | 2.191 | O(75) | -2.053 | -0.262 | 3.486 | H(294) | -10.04 | 17.523 | 5.871 |
| O(76) | -4.662 | -2.024 | 3.151 | H(295) | -8.826 | 18.464 | 0.795 | O(76) | -4.03 | -2.718 | 3.671 | H(295) | -11.073 | 16.737 | 4.622 |
| O(77) | -5.686 | -5.184 | 3.482 | Ru(296) | 8.041 | 9.226 | 1.441 | O(77) | -4.965 | -5.904 | 3.441 | Ru(296) | 10.044 | 8.651 | 0.841 |
| Ru(78) | -6.61 | 4.261 | -3.112 | C(297) | 7.568 | 10.309 | -0.206 | Ru(78) | -8.086 | 3.661 | -2.047 | C(297) | 10.004 | 9.469 | -1.013 |
| C(79) | -5.273 | 5.595 | -2.367 | C(298) | 6.336 | 10.355 | -0.735 | C(79) | -6.809 | 5.197 | -1.697 | C(298) | 9.023 | 9.252 | -1.906 |
| C(80) | -3.94 | 5.551 | -2.546 | C(299) | 6.125 | 11.009 | -1.885 | C(80) | -5.666 | 5.429 | -2.365 | C(299) | 9.057 | 9.866 | -3.096 |
| C(81) | -3.132 | 6.324 | -1.801 | C(300) | 7.129 | 11.625 | -2.529 | C(81) | -4.879 | 6.459 | -2.01 | C(300) | 10.041 | 10.73 | -3.394 |
| C(82) | -3.633 | 7.115 | -0.836 | C(301) | 8.357 | 11.572 | -1.975 | C(82) | -5.165 | 7.225 | -0.944 | C(301) | 11.039 | 10.897 | -2.507 |
| C(83) | -4.968 | 7.167 | -0.697 | C(302) | 8.592 | 10.935 | -0.811 | C(83) | -6.301 | 6.955 | -0.279 | C(302) | 11.051 | 10.258 | -1.321 |
| C(84) | -5.798 | 6.465 | -1.487 | C(303) | 9.127 | 7.749 | 0.571 | C(84) | -7.167 | 6.014 | -0.692 | C(303) | 11.023 | 6.974 | 0.259 |
| C(85) | -6.085 | 3.37 | -1.367 | C(304) | 9.789 | 6.914 | 1.397 | C(85) | -7.5 | 2.45 | -0.528 | C(304) | 11.154 | 6.025 | 1.207 |
| C(86) | -4.954 | 2.644 | -1.431 | C(305) | 10.512 | 5.921 | 0.83 | C(86) | -6.919 | 1.291 | -0.896 | C(305) | 11.555 | 4.801 | 0.801 |
| C(87) | -4.446 | 2.206 | -0.263 | C(306) | 10.443 | 5.627 | -0.489 | C(87) | -6.583 | 0.413 | 0.074 | C(306) | 11.702 | 4.478 | -0.502 |
| C(88) | -5.074 | 2.327 | 0.927 | C(307) | 9.741 | 6.471 | -1.265 | C(88) | -6.659 | 0.711 | 1.389 | C(307) | 11.636 | 5.47 | -1.402 |
| C(89) | -6.237 | 3.001 | 0.927 | C(308) | 9.124 | 7.544 | -0.757 | C(89) | -7.213 | 1.888 | 1.717 | C(308) | 11.33 | 6.716 | -1.024 |
| C(90) | -6.73 | 3.538 | -0.199 | C(309) | 12.077 | 11.184 | 0.115 | C(90) | -7.66 | 2.732 | 0.777 | C(309) | 14.163 | 10.883 | 0.416 |
| C(91) | -9.185 | 7.605 | -0.961 | C(310) | 12 | 10.659 | 1.349 | C(91) | -10.291 | 6.466 | 1.072 | C(310) | 13.89 | 10.25 | 1.569 |
| C(92) | -9.81 | 6.708 | -1.744 | C(311) | 10.831 | 10.212 | 1.827 | C(92) | -10.98 | 5.467 | 0.494 | C(311) | 12.713 | 9.633 | 1.744 |
| C(93) | -9.129 | 5.752 | -2.397 | C(312) | 9.734 | 10.273 | 1.054 | C(93) | -10.415 | 4.669 | -0.425 | C(312) | 11.805 | 9.649 | 0.754 |
| C(94) | -7.791 | 5.684 | -2.281 | C(313) | 9.766 | 10.879 | -0.149 | C(94) | -9.134 | 4.857 | -0.787 | C(313) | 12.031 | 10.313 | -0.395 |
| C(95) | -7.141 | 6.569 | -1.506 | C(314) | 10.948 | 11.338 | -0.599 | C(95) | -8.423 | 5.854 | -0.229 | C(314) | 13.219 | 10.929 | -0.541 |
| C(96) | -7.849 | 7.504 | -0.849 | C(315) | 8.707 | 8.127 | 3.008 | C(96) | -9.014 | 6.639 | 0.69 | C(315) | 10.255 | 7.624 | 2.575 |
| C(97) | -5.221 | 2.904 | -3.691 | C(316) | 8.24 | 8.315 | 4.253 | C(97) | -6.935 | 2.264 | -2.96 | C(316) | 9.706 | 8.044 | 3.728 |
| C(98) | -4.996 | 2.526 | -4.962 | C(317) | 8.518 | 7.415 | 5.203 | C(98) | -6.499 | 2.349 | -4.229 | C(317) | 9.517 | 7.17 | 4.725 |
| C(99) | -4.078 | 1.584 | -5.233 | C(318) | 9.334 | 6.376 | 4.961 | C(99) | -5.716 | 1.385 | -4.738 | C(318) | 9.978 | 5.913 | 4.637 |
| C(100) | -3.311 | 1.049 | -4.266 | C(319) | 9.912 | 6.3 | 3.74 | C(100) | -5.416 | 0.274 | -4.041 | C(319) | 10.692 | 5.577 | 3.54 |
| C(101) | -3.517 | 1.518 | -3.017 | C(320) | 9.538 | 7.104 | 2.718 | C(101) | -5.956 | 0.178 | -2.807 | C(320) | 10.749 | 6.377 | 2.452 |
| C(102) | -4.513 | 2.362 | -2.682 | C(321) | 10.98 | 4.378 | -1.218 | C(102) | -6.62 | 1.189 | -2.212 | C(321) | 11.846 | 3.06 | -1.069 |
| C(103) | -4.555 | 1.761 | 2.259 | O(322) | 11.81 | 3.43 | -0.55 | C(103) | -6.12 | -0.15 | 2.541 | O(322) | 11.744 | 1.975 | -0.15 |
| O(104) | -5.091 | 0.49 | 2.656 | C(323) | 9.415 | 5.334 | 6.093 | O(104) | -5.557 | -1.411 | 2.196 | C(323) | 9.562 | 4.956 | 5.765 |
| C(105) | -2.276 | -0.029 | -4.646 | O(324) | 10.071 | 4.089 | 5.844 | C(105) | -4.457 | -0.744 | -4.691 | O(324) | 9.937 | 3.586 | 5.641 |
| O(106) | -1.25 | -0.258 | -3.693 | O(325) | 8.116 | 4.758 | 6.313 | O(106) | -3.077 | -0.365 | -4.573 | O(325) | 8.144 | 4.712 | 5.734 |
| O(107) | -2.749 | -1.386 | -4.669 | O(326) | 9.906 | 3.464 | -1.452 | O(107) | -4.312 | -2.022 | -4.07 | O(326) | 10.683 | 2.705 | -1.837 |
| O(108) | -3.197 | 1.35 | 2.254 | C(327) | 13.425 | 11.52 | -0.488 | O(108) | -4.934 | 0.399 | 3.138 | C(327) | 15.522 | 11.503 | 0.182 |
| C(109) | -9.978 | 8.702 | -0.27 | C(328) | 13.911 | 10.319 | -1.329 | C(109) | -10.978 | 7.361 | 2.089 | C(328) | 16.436 | 10.584 | -0.651 |
| C(110) | -9.144 | 9.872 | 0.282 | C(329) | 13.983 | 9.016 | -0.513 | C(110) | -10.15 | 8.53 | 2.651 | C(329) | 16.811 | 9.252 | 0.029 |
| C(111) | -9.988 | 11.061 | 0.766 | C(330) | 14.419 | 7.783 | -1.319 | C(111) | -10.973 | 9.445 | 3.573 | C(330) | 15.842 | 8.084 | -0.238 |
| C(112) | -9.077 | 12.217 | 1.214 | C(331) | 14.514 | 6.554 | -0.398 | C(112) | -10.13 | 10.62 | 4.097 | C(331) | 16.267 | 6.816 | 0.524 |
| C(113) | -9.816 | 13.517 | 1.571 | C(332) | 14.884 | 5.265 | -1.145 | C(113) | -10.895 | 11.533 | 5.07 | C(332) | 15.308 | 5.632 | 0.312 |
| C(114) | -10.588 | 14.116 | 0.384 | C(333) | 15.193 | 4.127 | -0.16 | C(114) | -11.947 | 12.417 | 4.381 | C(333) | 15.796 | 4.371 | 1.048 |
| C(115) | -11.023 | 15.567 | 0.632 | C(334) | 15.504 | 2.802 | -0.872 | C(115) | -12.699 | 13.298 | 5.393 | C(334) | 14.874 | 3.163 | 0.823 |
| C(116) | -11.814 | 16.135 | -0.557 | C(335) | 16.026 | 1.751 | 0.116 | C(116) | -13.681 | 14.26 | 4.707 | C(335) | 15.368 | 1.9 | 1.54 |
| C(117) | -12.18 | 17.609 | -0.342 | C(336) | 6.874 | 12.365 | -3.831 | C(117) | -14.434 | 15.127 | 5.724 | C(336) | 10.006 | 11.484 | -4.707 |
| C(118) | -2.739 | 7.91 | 0.094 | C(337) | 5.833 | 13.498 | -3.722 | C(118) | -4.253 | 8.353 | -0.491 | C(337) | 9.174 | 12.778 | -4.601 |
| C(119) | -2.664 | 9.413 | -0.219 | C(338) | 4.427 | 13.105 | -4.21 | C(119) | -4.779 | 9.779 | -0.759 | C(338) | 7.725 | 12.538 | -4.144 |
| C(120) | -3.984 | 10.177 | -0.026 | C(339) | 3.397 | 14.233 | -4.031 | C(120) | -6.062 | 10.162 | -0.006 | C(339) | 6.841 | 13.793 | -4.217 |
| C(121) | -3.849 | 11.688 | -0.27 | C(340) | 2.004 | 13.804 | -4.522 | C(121) | -6.56 | 11.583 | -0.31 | C(340) | 5.429 | 13.513 | -3.671 |
| C(122) | -5.214 | 12.399 | -0.235 | C(341) | 0.929 | 14.869 | -4.26 | C(122) | -7.785 | 11.932 | 0.553 | C(341) | 4.485 | 14.713 | -3.842 |
| C(123) | -5.25 | 13.653 | -1.124 | C(342) | -0.475 | 14.377 | -4.651 | C(123) | -8.417 | 13.273 | 0.152 | C(342) | 3.096 | 14.437 | -3.244 |
| C(124) | -6.637 | 14.315 | -1.12 | C(343) | -1.59 | 15.287 | -4.111 | C(124) | -9.64 | 13.607 | 1.021 | C(343) | 2.101 | 15.568 | -3.548 |
| C(125) | -6.681 | 15.572 | -2.001 | C(344) | -2.988 | 14.727 | -4.405 | C(125) | -10.275 | 14.948 | 0.627 | C(344) | 0.729 | 15.308 | -2.914 |
| C(126) | -8.073 | 16.217 | -1.988 | N(345) | 6.569 | 8.006 | 1.162 | C(126) | -11.488 | 15.28 | 1.506 | N(345) | 8.372 | 7.715 | 0.596 |
| N(127) | -8.022 | 2.977 | -3.411 | C(346) | 5.749 | 7.712 | 2.056 | N(127) | -9.55 | 2.489 | -2.517 | C(346) | 7.531 | 7.554 | 1.505 |
| C(128) | -7.893 | 1.775 | -3.093 | S(347) | 4.708 | 7.32 | 3.122 | C(128) | -9.948 | 1.57 | -1.769 | S(347) | 6.463 | 7.318 | 2.591 |
| S(129) | -7.74 | 0.292 | -2.705 | N(348) | 7.354 | 10.688 | 2.497 | S(129) | -10.459 | 0.432 | -0.865 | N(348) | 9.268 | 10.253 | 1.59 |
| N(130) | -6.915 | 5.075 | -4.836 | C(349) | 6.132 | 10.951 | 2.532 | N(130) | -8.557 | 4.675 | -3.623 | C(349) | 8.296 | 10.826 | 1.054 |
| C(131) | -6.117 | 5.913 | -5.307 | S(350) | 4.632 | 11.297 | 2.593 | C(131) | -9.738 | 5.003 | -3.867 | S(350) | 7.101 | 11.557 | 0.412 |
| S(132) | -5.148 | 6.944 | -5.914 | H(351) | 5.474 | 9.862 | -0.26 | S(132) | -11.188 | 5.414 | -4.187 | H(351) | 8.163 | 8.589 | -1.718 |
| H(133) | -3.457 | 4.862 | -3.26 | H(352) | 5.108 | 11.02 | -2.3 | H(133) | -5.347 | 4.807 | -3.218 | H(352) | 8.244 | 9.681 | -3.817 |
| H(134) | -2.04 | 6.255 | -1.942 | H(353) | 9.167 | 12.101 | -2.499 | H(134) | -3.962 | 6.656 | -2.588 | H(353) | 11.846 | 11.588 | -2.792 |
| H(135) | -5.358 | 7.812 | 0.102 | H(354) | 11.108 | 5.269 | 1.479 | H(135) | -6.514 | 7.577 | 0.599 | H(354) | 11.65 | 3.994 | 1.538 |
| H(136) | -3.476 | 1.706 | -0.288 | H(355) | 9.648 | 6.273 | -2.346 | H(136) | -6.156 | -0.557 | -0.204 | H(355) | 11.795 | 5.251 | -2.47 |
| H(137) | -6.792 | 3.127 | 1.872 | H(356) | 8.569 | 8.181 | -1.467 | H(137) | -7.297 | 2.171 | 2.78 | H(356) | 11.291 | 7.472 | -1.826 |
| H(138) | -7.688 | 4.08 | -0.122 | H(357) | 12.91 | 10.543 | 1.961 | H(138) | -8.102 | 3.668 | 1.152 | H(357) | 14.648 | 10.203 | 2.368 |
| H(139) | -10.905 | 6.764 | -1.864 | H(358) | 10.829 | 9.772 | 2.839 | H(139) | -12.035 | 5.305 | 0.773 | H(358) | 12.549 | 9.109 | 2.702 |
| H(140) | -9.704 | 5.057 | -3.032 | H(359) | 11.047 | 11.777 | -1.602 | H(140) | -11.05 | 3.882 | -0.865 | H(359) | 13.482 | 11.449 | -1.474 |
| H(141) | -7.327 | 8.242 | -0.23 | H(360) | 7.558 | 9.138 | 4.53 | H(141) | -8.458 | 7.468 | 1.142 | H(360) | 9.314 | 9.065 | 3.875 |
| H(142) | -5.578 | 2.93 | -5.808 | H(361) | 8.04 | 7.52 | 6.192 | H(142) | -6.705 | 3.224 | -4.869 | H(361) | 8.956 | 7.488 | 5.62 |
| H(143) | -3.945 | 1.251 | -6.276 | H(362) | 10.6 | 5.466 | 3.549 | H(143) | -5.305 | 1.513 | -5.754 | H(362) | 11.118 | 4.567 | 3.491 |
| H(144) | -2.881 | 1.141 | -2.216 | H(363) | 11.463 | 4.663 | -2.184 | H(144) | -5.793 | -0.739 | -2.234 | H(363) | 12.769 | 2.959 | -1.687 |
| H(145) | -4.708 | 2.51 | 3.076 | H(364) | 9.808 | 5.789 | 7.033 | H(145) | -6.911 | -0.295 | 3.317 | H(364) | 9.871 | 5.347 | 6.763 |
| H(146) | -1.809 | 0.226 | -5.63 | H(365) | 13.351 | 12.434 | -1.123 | H(146) | -4.722 | -0.889 | -5.767 | H(365) | 15.392 | 12.48 | -0.34 |
| H(147) | -10.706 | 9.116 | -1.009 | H(366) | 14.168 | 11.75 | 0.309 | H(147) | -11.894 | 7.778 | 1.608 | H(366) | 16.015 | 11.746 | 1.152 |
| H(148) | -10.567 | 8.245 | 0.56 | H(367) | 13.22 | 10.168 | -2.193 | H(148) | -11.313 | 6.722 | 2.94 | H(367) | 15.986 | 10.386 | -1.651 |
| H(149) | -8.515 | 9.524 | 1.136 | H(368) | 14.918 | 10.548 | -1.753 | H(149) | -9.279 | 8.131 | 3.224 | H(368) | 17.379 | 11.154 | -0.842 |
| H(150) | -8.467 | 10.242 | -0.526 | H(369) | 14.683 | 9.17 | 0.343 | H(150) | -9.757 | 9.156 | 1.815 | H(369) | 17.815 | 8.935 | -0.346 |
| H(151) | -10.644 | 11.391 | -0.071 | H(370) | 12.979 | 8.775 | -0.09 | H(151) | -11.854 | 9.833 | 3.011 | H(370) | 16.925 | 9.417 | 1.125 |
| H(152) | -10.652 | 10.75 | 1.607 | H(371) | 13.681 | 7.586 | -2.133 | H(152) | -11.362 | 8.85 | 4.433 | H(371) | 14.806 | 8.351 | 0.063 |
| H(153) | -8.478 | 11.884 | 2.096 | H(372) | 15.408 | 7.976 | -1.796 | H(153) | -9.239 | 10.203 | 4.627 | H(372) | 15.812 | 7.868 | -1.333 |
| H(154) | -8.346 | 12.442 | 0.403 | H(373) | 15.283 | 6.758 | 0.386 | H(154) | -9.742 | 11.226 | 3.244 | H(373) | 17.292 | 6.522 | 0.196 |
| H(155) | -10.513 | 13.338 | 2.423 | H(374) | 13.541 | 6.397 | 0.127 | H(155) | -11.378 | 10.912 | 5.86 | H(374) | 16.32 | 7.045 | 1.616 |
| H(156) | -9.057 | 14.257 | 1.919 | H(375) | 14.047 | 4.97 | -1.82 | H(156) | -10.16 | 12.198 | 5.586 | H(375) | 14.291 | 5.909 | 0.678 |
| H(157) | -9.95 | 14.08 | -0.531 | H(376) | 15.781 | 5.449 | -1.784 | H(157) | -11.442 | 13.063 | 3.625 | H(376) | 15.221 | 5.416 | -0.779 |
| H(158) | -11.499 | 13.503 | 0.192 | H(377) | 16.071 | 4.42 | 0.465 | H(158) | -12.686 | 11.787 | 3.835 | H(377) | 16.823 | 4.113 | 0.695 |
| H(159) | -11.645 | 15.62 | 1.557 | H(378) | 14.331 | 3.979 | 0.533 | H(159) | -13.256 | 12.645 | 6.106 | H(378) | 15.862 | 4.583 | 2.142 |
| H(160) | -10.119 | 16.196 | 0.801 | H(379) | 14.581 | 2.425 | -1.374 | H(160) | -11.966 | 13.887 | 5.992 | H(379) | 13.854 | 3.404 | 1.198 |
| H(161) | -11.21 | 16.042 | -1.491 | H(380) | 16.272 | 2.966 | -1.665 | H(161) | -13.124 | 14.926 | 4.006 | H(380) | 14.794 | 2.954 | -0.27 |
| H(162) | -12.746 | 15.539 | -0.706 | H(381) | 16.161 | 0.763 | -0.382 | H(162) | -14.415 | 13.677 | 4.101 | H(381) | 14.686 | 1.041 | 1.345 |
| H(163) | -12.762 | 18.006 | -1.206 | H(382) | 17.011 | 2.052 | 0.541 | H(163) | -15.14 | 15.821 | 5.212 | H(382) | 16.385 | 1.611 | 1.192 |
| H(164) | -12.799 | 17.74 | 0.574 | H(383) | 15.322 | 1.619 | 0.967 | H(164) | -15.027 | 14.499 | 6.428 | H(383) | 15.409 | 2.055 | 2.643 |
| H(165) | -11.266 | 18.238 | -0.233 | H(384) | 7.832 | 12.802 | -4.2 | H(165) | -13.73 | 15.746 | 6.326 | H(384) | 11.042 | 11.729 | -5.039 |
| H(166) | -3.078 | 7.764 | 1.147 | H(385) | 6.568 | 11.626 | -4.607 | H(166) | -4.052 | 8.233 | 0.599 | H(385) | 9.581 | 10.831 | -5.505 |
| H(167) | -1.712 | 7.482 | 0.054 | H(386) | 5.796 | 13.879 | -2.675 | H(167) | -3.259 | 8.25 | -0.991 | H(386) | 9.67 | 13.479 | -3.888 |
| H(168) | -1.883 | 9.88 | 0.428 | H(387) | 6.165 | 14.357 | -4.355 | H(168) | -3.981 | 10.506 | -0.472 | H(387) | 9.164 | 13.278 | -5.599 |
| H(169) | -2.328 | 9.526 | -1.274 | H(388) | 4.476 | 12.816 | -5.287 | H(169) | -4.948 | 9.914 | -1.85 | H(388) | 7.266 | 11.744 | -4.781 |
| H(170) | -4.73 | 9.787 | -0.756 | H(389) | 4.063 | 12.215 | -3.652 | H(170) | -6.892 | 9.481 | -0.302 | H(389) | 7.724 | 12.176 | -3.088 |
| H(171) | -4.379 | 10.004 | 1.002 | H(390) | 3.34 | 14.504 | -2.949 | H(171) | -5.89 | 10.05 | 1.091 | H(390) | 7.31 | 14.617 | -3.629 |
| H(172) | -3.171 | 12.134 | 0.493 | H(391) | 3.726 | 15.141 | -4.59 | H(172) | -5.75 | 12.324 | -0.124 | H(391) | 6.772 | 14.133 | -5.277 |
| H(173) | -3.364 | 11.849 | -1.259 | H(392) | 2.042 | 13.578 | -5.614 | H(173) | -6.828 | 11.65 | -1.392 | H(392) | 4.994 | 12.633 | -4.203 |
| H(174) | -6.01 | 11.705 | -0.589 | H(393) | 1.716 | 12.864 | -3.993 | H(174) | -8.556 | 11.133 | 0.457 | H(393) | 5.497 | 13.245 | -2.589 |
| H(175) | -5.475 | 12.66 | 0.818 | H(394) | 0.944 | 15.121 | -3.175 | H(175) | -7.487 | 11.964 | 1.627 | H(394) | 4.928 | 15.612 | -3.351 |
| H(176) | -4.478 | 14.38 | -0.78 | H(395) | 1.169 | 15.806 | -4.816 | H(176) | -7.655 | 14.082 | 0.245 | H(395) | 4.383 | 14.941 | -4.929 |
| H(177) | -4.994 | 13.37 | -2.172 | H(396) | -0.549 | 14.297 | -5.761 | H(177) | -8.728 | 13.229 | -0.918 | H(396) | 2.697 | 13.485 | -3.664 |
| H(178) | -7.395 | 13.587 | -1.495 | H(397) | -0.627 | 13.35 | -4.248 | H(178) | -10.399 | 12.796 | 0.924 | H(397) | 3.185 | 14.298 | -2.14 |
| H(179) | -6.92 | 14.586 | -0.076 | H(398) | -1.482 | 15.401 | -3.006 | H(179) | -9.337 | 13.644 | 2.094 | H(398) | 2.505 | 16.536 | -3.168 |
| H(180) | -5.927 | 16.311 | -1.638 | H(399) | -1.489 | 16.303 | -4.563 | H(180) | -9.518 | 15.763 | 0.72 | H(399) | 1.979 | 15.667 | -4.654 |
| H(181) | -6.408 | 15.304 | -3.049 | H(400) | -3.782 | 15.409 | -4.021 | H(181) | -10.595 | 14.91 | -0.442 | H(400) | 0.002 | 16.108 | -3.182 |
| H(182) | -8.096 | 17.136 | -2.618 | H(401) | -3.149 | 14.596 | -5.499 | H(182) | -11.943 | 16.253 | 1.212 | H(401) | 0.303 | 14.336 | -3.252 |
| H(183) | -8.842 | 15.514 | -2.386 | H(402) | -3.137 | 13.737 | -3.916 | H(183) | -12.274 | 14.495 | 1.421 | H(402) | 0.807 | 15.286 | -1.803 |
| H(184) | -8.372 | 16.507 | -0.956 | O(403) | 14.323 | -1.1 | 1.197 | H(184) | -11.192 | 15.356 | 2.576 | C(403) | -3.196 | 4.413 | 0.148 |
| O(185) | -6.58 | -8.214 | 4.22 | H(404) | 14.781 | -0.453 | 1.705 | O(185) | -5.555 | -9.058 | 3.921 | C(404) | -3.628 | 3.337 | -0.532 |
| H(186) | -7.264 | -8.199 | 3.574 | H(405) | 8.153 | -0.974 | -1.848 | H(186) | -6.309 | -9.03 | 3.357 | C(405) | -3.235 | 3.162 | -1.807 |
| O(187) | -4.539 | -10.011 | -2.637 | H(406) | 5.006 | 1.151 | 6.141 | O(187) | -4.226 | -10.009 | -3.194 | C(406) | -2.407 | 4.061 | -2.367 |
| H(188) | -5.449 | -9.794 | -2.526 | O(407) | 11.005 | -1.042 | 7.552 | H(188) | -5.128 | -9.878 | -2.957 | C(407) | -1.975 | 5.13 | -1.684 |
| Ru(189) | 1.923 | 8.787 | -2.219 | H(408) | 10.644 | -1.641 | 6.92 | Ru(189) | 1.227 | 9.762 | -2.249 | C(408) | -2.378 | 5.31 | -0.418 |
| C(190) | 1.518 | 10.65 | -1.529 | O(409) | 7.328 | -0.717 | -1.476 | C(190) | 0.129 | 11.323 | -1.567 | O(409) | -3.64 | 2.064 | -2.495 |
| C(191) | 2.319 | 11.344 | -0.702 | O(410) | 4.543 | 1.27 | 5.33 | C(191) | 0.583 | 12.233 | -0.687 | O(410) | -4.416 | 2.408 | 0.066 |
| C(192) | 1.962 | 12.57 | -0.288 |  |  |  |  | C(192) | -0.221 | 13.223 | -0.275 | H(411) | -3.512 | 4.552 | 1.196 |
| C(193) | 0.806 | 13.117 | -0.696 |  |  |  |  | C(193) | -1.473 | 13.329 | -0.749 | H(412) | -2.07 | 3.911 | -3.406 |
| C(194) | 0.029 | 12.414 | -1.538 |  |  |  |  | C(194) | -1.903 | 12.42 | -1.641 | H(413) | -1.3 | 5.858 | -2.162 |
| C(195) | 0.362 | 11.182 | -1.966 |  |  |  |  | C(195) | -1.121 | 11.407 | -2.059 | H(414) | -2.039 | 6.186 | 0.157 |
| C(196) | 1.968 | 6.905 | -2.968 |  |  |  |  | C(196) | 1.887 | 7.97 | -2.927 | H(415) | -3.966 | 1.365 | -1.898 |
| C(197) | 1.461 | 5.946 | -2.168 |  |  |  |  | C(197) | 1.523 | 6.919 | -2.172 | H(416) | -4.386 | 1.561 | -0.418 |
| C(198) | 1.561 | 4.673 | -2.608 |  |  |  |  | C(198) | 1.792 | 5.696 | -2.667 | C(417) | 6.053 | 6.349 | -2.893 |
| C(199) | 2.277 | 4.337 | -3.705 |  |  |  |  | C(199) | 2.545 | 5.46 | -3.763 | C(418) | 6.733 | 5.893 | -1.829 |
| C(200) | 2.767 | 5.327 | -4.467 |  |  |  |  | C(200) | 2.919 | 6.549 | -4.458 | C(419) | 8.006 | 5.485 | -1.984 |
| C(201) | 2.58 | 6.607 | -4.127 |  |  |  |  | C(201) | 2.573 | 7.787 | -4.069 | C(420) | 8.564 | 5.522 | -3.204 |
| C(202) | -2.209 | 9.953 | -4.198 |  |  |  |  | C(202) | -2.905 | 9.459 | -4.536 | C(421) | 7.886 | 5.995 | -4.261 |
| C(203) | -1.713 | 8.727 | -4.423 |  |  |  |  | C(203) | -1.986 | 8.502 | -4.735 | C(422) | 6.624 | 6.417 | -4.104 |
| C(204) | -0.559 | 8.344 | -3.856 |  |  |  |  | C(204) | -0.827 | 8.517 | -4.062 | O(423) | 8.679 | 5.007 | -0.908 |
| C(205) | 0.119 | 9.187 | -3.056 |  |  |  |  | C(205) | -0.556 | 9.499 | -3.181 | O(424) | 6.146 | 5.789 | -0.608 |
| C(206) | -0.369 | 10.415 | -2.799 |  |  |  |  | C(206) | -1.477 | 10.454 | -2.946 | H(425) | 5.008 | 6.666 | -2.755 |
| C(207) | -1.53 | 10.774 | -3.379 |  |  |  |  | C(207) | -2.642 | 10.41 | -3.622 | H(426) | 9.595 | 5.162 | -3.342 |
| C(208) | 1.139 | 7.682 | -0.711 |  |  |  |  | C(208) | 0.811 | 8.516 | -0.703 | H(427) | 8.362 | 6.028 | -5.255 |
| C(209) | 0.932 | 8.184 | 0.518 |  |  |  |  | C(209) | 0.464 | 8.918 | 0.532 | H(428) | 6.054 | 6.799 | -4.966 |
| C(210) | 0.675 | 7.356 | 1.54 |  |  |  |  | C(210) | 0.337 | 8.025 | 1.526 | H(429) | 9.463 | 4.482 | -1.142 |
| C(211) | 0.524 | 6.035 | 1.345 |  |  |  |  | C(211) | 0.482 | 6.705 | 1.316 | H(430) | 6.674 | 5.163 | -0.077 |
| C(212) | 0.618 | 5.58 | 0.074 |  |  |  |  | C(212) | 0.748 | 6.338 | 0.044 | O(431) | 13.241 | -2.234 | 0.878 |
| C(213) | 1.025 | 6.361 | -0.951 |  |  |  |  | C(213) | 0.995 | 7.209 | -0.958 | H(432) | 13.79 | -1.506 | 1.116 |
| C(214) | 2.647 | 2.912 | -4.127 |  |  |  |  | C(214) | 3.007 | 4.073 | -4.258 | H(433) | 6.08 | -0.942 | -2.681 |
| O(215) | 2.188 | 1.871 | -3.28 |  |  |  |  | O(215) | 2.15 | 2.967 | -3.994 | H(434) | 4.611 | 1.367 | 6.056 |
| C(216) | 0.321 | 5.17 | 2.601 |  |  |  |  | C(216) | 0.322 | 5.752 | 2.52 | O(435) | 10.153 | -1.553 | 6.957 |
| O(217) | 1.502 | 5.098 | 3.418 |  |  |  |  | O(217) | 1.521 | 5.449 | 3.25 | H(436) | 9.708 | -2.068 | 6.306 |
| O(218) | 0.196 | 3.765 | 2.413 |  |  |  |  | O(218) | -0.016 | 4.402 | 2.234 | O(437) | 6.644 | -0.693 | -1.969 |
| O(219) | 4.046 | 2.633 | -3.957 |  |  |  |  | O(219) | 4.157 | 3.541 | -3.583 | O(438) | 4.268 | 1.325 | 5.18 |

**Table S7**. Cartesian coordinates of the two different MM2 simulation grids under solvation.

| **(Z907)_3_-(TiO_2_)_24_** | | | | | | | | **[(Z907)_3_+(Catechol)_2_]-(TiO_2_)_24_** | | | | | | | |
| --- | --- | --- | --- | --- | --- | --- | --- | --- | --- | --- | --- | --- | --- | --- | --- |
| Ti(1) | 8.1912 | -5.7877 | 7.2421 | H(356) | -10.3438 | -2.2421 | -1.0972 | Ti(1) | 7.6859 | -6.7652 | 7.4375 | H(356) | -10.8748 | -3.5046 | -0.9547 |
| Ti(2) | 10.1749 | -7.1596 | 1.3954 | H(357) | -13.8425 | -0.5515 | -2.6085 | Ti(2) | 9.7998 | -8.0402 | 1.6184 | H(357) | -14.3473 | -1.7619 | -2.5864 |
| Ti(3) | 9.2804 | -6.2786 | 4.3128 | H(358) | -12.5591 | -2.5539 | -2.9667 | Ti(3) | 8.8572 | -7.2137 | 4.5327 | H(358) | -12.9888 | -3.7267 | -2.8376 |
| Ti(4) | 6.3094 | -3.2188 | 5.8683 | H(359) | -10.5853 | 1.826 | -3.7306 | Ti(4) | 6.1842 | -3.9076 | 6.1285 | H(359) | -11.2117 | 0.6683 | -3.901 |
| Ti(5) | 7.2858 | -3.9217 | 2.9186 | H(360) | -8.5307 | -4.9587 | -6.3761 | Ti(5) | 7.1379 | -4.6049 | 3.1882 | H(360) | -8.8455 | -6.1171 | -6.1711 |
| Ti(6) | 8.5962 | -4.3766 | 0.0064 | H(361) | -6.5234 | -6.2335 | -6.6085 | Ti(6) | 8.5104 | -5.0696 | 0.3077 | H(361) | -6.7556 | -7.2592 | -6.3745 |
| Ti(7) | 3.1362 | -2.8981 | 4.4406 | H(362) | -5.2915 | -5.0086 | -2.7863 | Ti(7) | 3.0386 | -3.3572 | 4.6225 | H(362) | -5.5842 | -5.8156 | -2.6104 |
| Ti(8) | 4.3877 | -3.1259 | 1.4921 | H(363) | -1.8197 | 7.4701 | -2.2919 | Ti(8) | 4.2821 | -3.7518 | 1.6999 | H(363) | -2.1931 | 6.9423 | -2.2651 |
| Ti(9) | 5.3124 | -4.5273 | -1.2484 | H(364) | -0.1414 | 2.8563 | 2.6836 | Ti(9) | 5.2537 | -5.0893 | -1.0593 | H(364) | 0.1272 | 2.5713 | 2.5321 |
| Ti(10) | 0.0658 | -1.9228 | 3.0576 | H(365) | -0.8579 | -0.1278 | -0.2005 | Ti(10) | 0.0183 | -2.381 | 3.1119 | H(365) | -0.7058 | -0.5761 | -0.1147 |
| Ti(11) | 1.2702 | -2.9545 | 0.2746 | H(366) | -7.2951 | -3.0123 | 2.2552 | Ti(11) | 1.1744 | -3.6347 | 0.4034 | H(366) | -7.7262 | -3.8201 | 2.3826 |
| Ti(12) | 2.1571 | -3.905 | -2.6323 | H(367) | -4.1421 | -6.4306 | -5.9736 | Ti(12) | 2.0865 | -4.4938 | -2.5099 | H(367) | -4.3569 | -7.2493 | -5.7591 |
| Ti(13) | -2.4826 | -4.1262 | 2.7069 | H(368) | 0.9679 | -2.0607 | -5.2336 | Ti(13) | -2.8316 | -4.2443 | 2.7506 | H(368) | 0.9825 | -2.746 | -5.1538 |
| Ti(14) | -1.7134 | -4.7103 | -0.21 | H(369) | -12.956 | 2.5581 | -4.0023 | Ti(14) | -1.8672 | -5.2557 | -0.0977 | H(369) | -13.7316 | 1.2048 | -4.2139 |
| Ti(15) | -0.4188 | -6.1255 | -2.6192 | H(370) | -14.1711 | 1.7554 | -3.0008 | Ti(15) | -0.5774 | -6.6591 | -2.5189 | H(370) | -14.7532 | 0.5331 | -2.9297 |
| O(16) | 4.3415 | -3.1491 | 5.9798 | H(371) | -12.9651 | 2.4123 | -0.9104 | O(16) | 4.3098 | -3.2883 | 6.1287 | H(371) | -13.2942 | 1.5172 | -1.1632 |
| O(17) | 1.4248 | -1.9213 | 4.4878 | H(372) | -11.6194 | 3.1039 | -1.8722 | O(17) | 1.2018 | -2.6413 | 4.6691 | H(372) | -12.1172 | 2.0774 | -2.3848 |
| O(18) | 1.3891 | -5.6421 | -3.153 | H(373) | -13.0865 | 4.7809 | -2.9168 | O(18) | 1.2518 | -6.207 | -3.0114 | H(373) | -13.7914 | 3.5442 | -3.4653 |
| O(19) | 10.1869 | -5.362 | 0.6251 | H(374) | -14.5333 | 4.1156 | -2.1106 | O(19) | 9.9831 | -6.2089 | 0.9568 | H(374) | -15.052 | 3.0398 | -2.3041 |
| O(20) | 7.2107 | -5.0679 | -1.219 | H(375) | -12.1053 | 5.0727 | -0.4679 | O(20) | 7.1544 | -5.6213 | -1.0176 | H(375) | -12.3682 | 4.263 | -1.3753 |
| O(21) | 4.0753 | -4.3419 | -2.7713 | H(376) | -13.2706 | 6.2679 | -1.0964 | O(21) | 4.0382 | -4.7815 | -2.5816 | H(376) | -13.7065 | 5.3172 | -1.9 |
| O(22) | 8.9538 | -4.3956 | 3.8484 | H(377) | -14.2293 | 3.8388 | 0.5248 | O(22) | 8.7307 | -5.2958 | 4.1151 | H(377) | -14.1446 | 3.168 | 0.2578 |
| O(23) | 6.243 | -2.6138 | 1.8798 | H(378) | -13.6419 | 5.3256 | 1.3325 | O(23) | 6.1365 | -3.2751 | 2.1385 | H(378) | -13.4777 | 4.7769 | 0.6644 |
| O(24) | 2.9467 | -2.0293 | 0.7355 | H(379) | -15.3366 | 6.7041 | 0.1712 | O(24) | 2.831 | -2.6966 | 0.9035 | H(379) | -15.4581 | 5.9174 | -0.2715 |
| O(25) | -0.5789 | -3.1046 | -0.3904 | H(380) | -15.9663 | 5.2894 | -0.7273 | O(25) | -0.6631 | -3.7071 | -0.3015 | H(380) | -16.1925 | 4.3692 | -0.7886 |
| O(26) | 10.7365 | -6.1406 | 2.9995 | H(381) | -15.9096 | 5.4222 | 2.364 | O(26) | 10.3857 | -7.1662 | 3.2973 | H(381) | -15.4632 | 4.9596 | 2.1584 |
| O(27) | 8.8616 | -7.4732 | 2.8103 | H(382) | -17.2747 | 6.0492 | 1.3875 | O(27) | 8.407 | -8.309 | 2.965 | H(382) | -17.0342 | 5.4352 | 1.4402 |
| O(28) | 9.711 | -5.4765 | 6.0549 | H(383) | -17.6932 | 3.818 | 0.3885 | O(28) | 9.2838 | -6.5515 | 6.3336 | H(383) | -17.5972 | 3.0913 | 0.8887 |
| O(29) | 7.8464 | -6.7758 | 5.5633 | H(384) | -16.2911 | 3.1153 | 1.2569 | O(29) | 7.3156 | -7.6088 | 5.6877 | H(384) | -16.004 | 2.5154 | 1.4711 |
| O(30) | 6.8933 | -2.4499 | 4.1583 | H(385) | -5.4651 | 1.7638 | -6.194 | O(30) | 6.8313 | -3.1252 | 4.4463 | H(385) | -6.2207 | 0.7466 | -6.4669 |
| O(31) | 7.4188 | -5.1427 | 1.3863 | H(386) | -6.8758 | 2.7755 | -5.8038 | O(31) | 7.2458 | -5.795 | 1.6299 | H(386) | -7.6714 | 1.6852 | -6.0565 |
| O(32) | 4.5661 | -4.8401 | 0.5452 | H(387) | -4.8249 | 3.2876 | -4.4104 | O(32) | 4.4669 | -5.4414 | 0.7101 | H(387) | -5.5915 | 2.4736 | -4.8591 |
| O(33) | 4.0332 | -1.9071 | 3.0005 | H(388) | -6.2644 | 2.8615 | -3.4274 | O(33) | 3.954 | -2.4474 | 3.1415 | H(388) | -6.9006 | 2.0058 | -3.7255 |
| O(34) | 0.7325 | -1.3651 | 1.2942 | H(389) | -5.401 | 0.636 | -2.9181 | O(34) | 0.5595 | -1.9831 | 1.2636 | H(389) | -5.8356 | -0.1131 | -3.1868 |
| O(35) | 1.9027 | -4.4894 | -0.7715 | H(390) | -4.153 | 0.7485 | -4.1986 | O(35) | 1.8391 | -5.1375 | -0.6681 | H(390) | -4.69 | 0.0287 | -4.56 |
| O(36) | -0.5231 | -6.2153 | -0.6683 | H(391) | -3.0636 | 1.0177 | -2.0108 | O(36) | -0.6559 | -6.7397 | -0.5643 | H(391) | -3.4467 | 0.4902 | -2.5397 |
| O(37) | -2.7336 | -3.4342 | 0.8998 | H(392) | -2.9639 | 2.515 | -2.9625 | O(37) | -2.9211 | -3.8755 | 0.8366 | H(392) | -3.5982 | 1.9902 | -3.4748 |
| O(38) | -1.4519 | -5.3879 | 1.6242 | H(393) | -5.0289 | 3.3781 | -1.6185 | O(38) | -1.7586 | -5.6232 | 1.8367 | H(393) | -5.5002 | 2.7245 | -1.9229 |
| O(39) | 1.9709 | -2.3409 | -1.4561 | H(394) | -4.849 | 1.9538 | -0.5708 | O(39) | 1.8594 | -2.9557 | -1.3083 | H(394) | -5.3086 | 1.2305 | -1.0016 |
| O(40) | 0.7889 | -3.4828 | 2.1021 | H(395) | -2.5842 | 2.6579 | 0.1077 | O(40) | 0.6697 | -4.0291 | 2.2572 | H(395) | -2.9295 | 1.8265 | -0.4385 |
| O(41) | 3.5692 | -4.133 | 2.9709 | H(396) | -2.5437 | 3.944 | -1.1459 | O(41) | 3.4125 | -4.671 | 3.2057 | H(396) | -3.0463 | 3.3005 | -1.4538 |
| O(42) | 5.0768 | -2.8075 | -0.3283 | H(397) | -4.4638 | 5.123 | -0.013 | O(42) | 5.013 | -3.3949 | -0.0962 | H(397) | -4.8769 | 4.1976 | 0.0176 |
| O(43) | 8.6206 | -3.2785 | 1.6358 | H(398) | -4.4443 | 3.8804 | 1.2887 | O(43) | 8.541 | -4.005 | 1.9595 | H(398) | -4.7647 | 2.7305 | 1.0552 |
| O(44) | 6.1919 | -4.5495 | 4.4225 | H(399) | -2.0832 | 4.512 | 1.8709 | O(44) | 5.9673 | -5.1875 | 4.6506 | H(399) | -2.3834 | 3.2742 | 1.6066 |
| O(45) | 6.4866 | -4.9056 | 6.8703 | H(400) | -2.0358 | 5.6901 | 0.5124 | O(45) | 6.131 | -5.6349 | 7.0767 | H(400) | -2.4238 | 4.7027 | 0.5123 |
| O(46) | -1.2468 | -3.1549 | 3.8534 | H(401) | 13.6351 | -0.2481 | -2.1734 | O(46) | -1.2839 | -3.6893 | 3.8001 | H(401) | 14.1238 | -0.4251 | -1.2512 |
| O(47) | -1.5949 | -4.6319 | -2.18 | H(402) | 11.0535 | -0.9721 | -3.7151 | O(47) | -1.775 | -5.1856 | -2.0693 | H(402) | 11.4766 | -1.1237 | -2.6874 |
| Ti(48) | -3.216 | -8.029 | -3.1663 | H(403) | 12.6667 | -0.7229 | -4.4655 | Ti(48) | -3.3009 | -8.71 | -2.9249 | H(403) | 13.0737 | -0.9426 | -3.4883 |
| Ti(49) | -3.1894 | -11.2195 | -1.7452 | H(404) | -18.458 | 2.6168 | 2.4489 | Ti(49) | -3.1327 | -11.8429 | -1.3766 | H(404) | -17.7942 | 2.1735 | 3.2141 |
| Ti(50) | -3.2094 | -14.2324 | -0.5047 | H(405) | -17.3725 | 3.6159 | 3.4765 | Ti(50) | -3.0315 | -14.8035 | -0.0195 | H(405) | -16.5337 | 3.3098 | 3.8051 |
| Ti(51) | -4.3287 | -7.047 | -0.3397 | H(406) | -18.7753 | 4.3748 | 2.6337 | Ti(51) | -4.3861 | -7.6716 | -0.1095 | H(406) | -18.1267 | 3.9385 | 3.2385 |
| Ti(52) | -5.002 | -10.219 | 0.6758 | H(407) | -2.4805 | 6.8533 | 2.6978 | Ti(52) | -4.9487 | -10.8181 | 1.0299 | H(407) | -2.743 | 5.391 | 2.9137 |
| Ti(53) | -5.0148 | -13.318 | 1.9329 | H(408) | -3.893 | 7.0266 | 1.5967 | Ti(53) | -4.8502 | -13.8655 | 2.3996 | H(408) | -4.223 | 5.7559 | 1.9567 |
| Ti(54) | -5.4576 | -5.7984 | 2.373 | H(409) | 8.1433 | 12.1644 | 2.0544 | Ti(54) | -5.5618 | -6.3453 | 2.5537 | H(409) | 7.4664 | 10.4014 | 3.1383 |
| Ti(55) | -5.4453 | -9.0561 | 3.605 | H(410) | 12.1954 | -3.1788 | -4.0625 | Ti(55) | -5.3967 | -9.5572 | 3.9176 | H(410) | 12.5342 | -3.3771 | -2.9875 |
| Ti(56) | -5.4761 | -12.0775 | 4.8126 | C(411) | -8.3847 | -7.685 | -2.296 | Ti(56) | -5.3175 | -12.5322 | 5.2375 | C(411) | -8.5245 | -8.3142 | -1.7825 |
| O(57) | -1.2821 | -7.7038 | -3.3656 | C(412) | -8.3835 | -9.1779 | -2.0038 | O(57) | -1.386 | -8.3096 | -3.1647 | C(412) | -8.7012 | -9.7049 | -1.1925 |
| O(58) | -3.716 | -9.9386 | -3.1309 | O(413) | -8.0324 | -7.4742 | -3.6466 | O(58) | -3.7173 | -10.6365 | -2.8056 | O(413) | -8.307 | -8.4128 | -3.1747 |
| O(59) | -3.9841 | -13.0051 | -1.8144 | H(414) | -9.3882 | -7.2373 | -2.1325 | O(59) | -3.8649 | -13.6563 | -1.3656 | H(414) | -9.4211 | -7.679 | -1.6184 |
| O(60) | -4.8053 | -7.3632 | -2.2181 | H(415) | -7.6476 | -7.1388 | -1.6694 | O(60) | -4.896 | -8.0834 | -1.9607 | H(415) | -7.646 | -7.7965 | -1.3451 |
| O(61) | -2.8402 | -7.9506 | -1.2384 | H(416) | -8.5881 | -9.3717 | -0.9257 | O(61) | -2.8861 | -8.5543 | -1.0099 | H(416) | -8.8055 | -9.6541 | -0.0841 |
| O(62) | -5.9183 | -6.2796 | 0.5224 | H(417) | -7.3992 | -9.6326 | -2.2574 | O(62) | -5.9951 | -6.9133 | 0.7216 | H(417) | -7.8251 | -10.3519 | -1.4262 |
| O(63) | -3.9966 | -6.8479 | 1.5806 | H(418) | -9.1625 | -9.7029 | -2.6022 | O(63) | -4.0524 | -7.3503 | 1.7941 | H(418) | -9.6106 | -10.2 | -1.603 |
| O(64) | -6.3717 | -9.5599 | 1.93 | H(419) | -7.8502 | -6.5393 | -3.7854 | O(64) | -6.3255 | -10.1593 | 2.2768 | H(419) | -8.0292 | -7.5562 | -3.5157 |
| O(65) | -4.0904 | -9.6509 | 2.3244 | C(420) | 12.2595 | 0.4553 | 2.0966 | O(65) | -4.0378 | -10.153 | 2.642 | C(420) | 3.8667 | 0.0933 | 0.2467 |
| O(66) | -6.38 | -13.0038 | 3.3144 | C(421) | 12.9152 | -0.7375 | 1.4156 | O(66) | -6.2089 | -13.5395 | 3.7848 | O(421) | 2.6454 | 0.4341 | 0.738 |
| O(67) | -4.1068 | -12.6891 | 3.5589 | O(422) | 11.4665 | 0.0114 | 3.1769 | O(67) | -3.9445 | -13.145 | 3.9885 | C(422) | 3.9809 | -0.3907 | -1.0011 |
| O(68) | -5.0557 | -14.2645 | 0.2086 | H(423) | 13.0165 | 1.1608 | 2.4965 | O(68) | -4.8694 | -14.8775 | 0.7125 | O(423) | 2.868 | -0.5232 | -1.7475 |
| O(69) | -3.1593 | -13.565 | 1.3319 | H(424) | 11.5951 | 1.0049 | 1.3967 | O(69) | -2.9917 | -14.0685 | 1.7914 | C(424) | 5.1721 | -0.7766 | -1.4803 |
| O(70) | -3.1514 | -10.538 | 0.0885 | H(425) | 13.5474 | -0.4073 | 0.5607 | O(70) | -3.0949 | -11.0918 | 0.4297 | C(425) | 6.2722 | -0.6643 | -0.726 |
| O(71) | -5.0526 | -10.8432 | -1.1928 | H(426) | 12.1541 | -1.4504 | 1.0246 | O(71) | -5.0011 | -11.5063 | -0.816 | C(426) | 6.1651 | -0.1999 | 0.526 |
| O(72) | -5.9737 | -11.9071 | 0.9549 | H(427) | 13.5709 | -1.2923 | 2.123 | O(72) | -5.8634 | -12.525 | 1.3786 | C(427) | 4.971 | 0.1768 | 1.0001 |
| O(73) | -5.5679 | -8.5748 | -0.2396 | H(428) | 10.875 | 0.7053 | 3.4905 | O(73) | -5.5837 | -9.2248 | 0.072 | H(428) | 1.9475 | 0.1542 | 0.1185 |
| O(74) | -3.5415 | -5.2759 | -0.6986 | C(429) | 11.65 | -2.6471 | 5.1773 | O(74) | -3.6838 | -5.8839 | -0.5483 | H(429) | 2.8992 | -1.2844 | -2.3499 |
| O(75) | -4.0141 | -5.045 | 3.484 | C(430) | 12.7681 | -1.81 | 5.7818 | O(75) | -4.0333 | -5.5711 | 3.53 | H(430) | 5.2414 | -1.1915 | -2.4994 |
| O(76) | -6.3428 | -7.3676 | 3.1797 | O(431) | 11.7882 | -2.7132 | 3.7721 | O(76) | -6.3574 | -7.9172 | 3.4452 | H(431) | 7.2415 | -0.986 | -1.1324 |
| O(77) | -6.5451 | -10.5565 | 4.2074 | H(432) | 11.6902 | -3.6858 | 5.5724 | O(77) | -6.4398 | -11.0669 | 4.593 | H(432) | 7.0444 | -0.1472 | 1.1801 |
| Ru(78) | -9.6519 | -3.0771 | -4.0794 | H(433) | 10.6513 | -2.2095 | 5.3934 | Ru(78) | -10.0573 | -4.1964 | -3.9451 | H(433) | 4.8714 | 0.5399 | 2.0306 |
| C(79) | -8.4912 | -1.4939 | -4.5932 | H(434) | 12.6441 | -1.7289 | 6.886 | C(79) | -8.9674 | -2.5854 | -4.5292 | C(434) | -6.1013 | -1.6787 | -0.8675 |
| C(80) | -7.3137 | -1.5814 | -5.2372 | H(435) | 12.7769 | -0.7779 | 5.368 | C(80) | -7.7978 | -2.6402 | -5.1923 | O(435) | -7.457 | -1.7666 | -0.867 |
| C(81) | -6.6004 | -0.4795 | -5.5016 | H(436) | 13.7658 | -2.2637 | 5.5883 | C(81) | -7.1472 | -1.5196 | -5.5357 | C(436) | -5.4165 | -2.282 | -1.854 |
| C(82) | -7.0476 | 0.7288 | -5.1298 | H(437) | 11.5233 | -1.8732 | 3.3923 | C(82) | -7.6527 | -0.3137 | -5.2341 | O(437) | -6.0912 | -2.9279 | -2.8304 |
| C(83) | -8.2473 | 0.8068 | -4.5302 | C(438) | 11.5502 | 7.2226 | 4.1751 | C(83) | -8.8401 | -0.2685 | -4.6088 | C(438) | -4.0771 | -2.2451 | -1.8637 |
| C(84) | -8.9909 | -0.2859 | -4.2759 | C(439) | 12.5306 | 6.7327 | 5.2309 | C(84) | -9.5165 | -1.384 | -4.2755 | C(439) | -3.4092 | -1.5843 | -0.9115 |
| C(85) | -8.7905 | -3.168 | -2.2468 | O(440) | 10.6801 | 6.1727 | 3.8022 | C(85) | -9.2289 | -4.266 | -2.0966 | C(440) | -4.0787 | -0.9798 | 0.0782 |
| C(86) | -7.6062 | -3.7998 | -2.2056 | H(441) | 10.9268 | 8.0642 | 4.5514 | C(86) | -7.989 | -4.7801 | -2.0469 | C(441) | -5.4179 | -1.038 | 0.0954 |
| C(87) | -7.0036 | -3.911 | -1.0058 | H(442) | 12.0999 | 7.5451 | 3.2627 | C(87) | -7.3597 | -4.7819 | -0.8556 | H(442) | -7.7409 | -2.4989 | -1.4436 |
| C(88) | -7.5917 | -3.5848 | 0.1646 | H(443) | 12.0049 | 6.3239 | 6.1229 | C(88) | -7.9672 | -4.482 | 0.3109 | H(443) | -5.6889 | -3.7973 | -2.9787 |
| C(89) | -8.7867 | -2.9783 | 0.076 | H(444) | 13.1926 | 7.5608 | 5.5731 | C(89) | -9.2301 | -4.0351 | 0.2201 | H(444) | -3.518 | -2.7439 | -2.6695 |
| C(90) | -9.363 | -2.7378 | -1.1105 | H(445) | 13.1821 | 5.9317 | 4.8154 | C(90) | -9.8416 | -3.8842 | -0.9643 | H(445) | -2.3134 | -1.5488 | -0.955 |
| C(91) | -12.2578 | 0.744 | -3.1387 | H(446) | 10.4765 | 6.2149 | 2.8586 | C(91) | -12.8289 | -0.4368 | -3.2155 | H(446) | -3.5333 | -0.4336 | 0.865 |
| C(92) | -12.791 | -0.4686 | -2.9237 | C(447) | 6.0674 | 8.3784 | 1.501 | C(92) | -13.3044 | -1.6563 | -2.9218 | H(447) | -5.9525 | -0.5253 | 0.9067 |
| C(93) | -12.0695 | -1.5804 | -3.1317 | C(448) | 6.3162 | 9.8417 | 1.8299 | C(93) | -12.5369 | -2.7479 | -3.0669 | C(448) | 12.8896 | -0.4057 | 2.7226 |
| C(94) | -10.8049 | -1.4878 | -3.5751 | O(449) | 7.2887 | 7.7289 | 1.2223 | C(94) | -11.2712 | -2.6327 | -3.5038 | C(449) | 13.6535 | -1.5018 | 1.9956 |
| C(95) | -10.2413 | -0.2828 | -3.7722 | H(450) | 5.5894 | 7.8498 | 2.3534 | C(95) | -10.7648 | -1.4159 | -3.7717 | O(450) | 12.019 | -1.0019 | 3.6596 |
| C(96) | -10.9759 | 0.8183 | -3.5324 | H(451) | 5.4275 | 8.2736 | 0.5996 | C(96) | -11.5536 | -0.3374 | -3.6215 | H(451) | 13.5647 | 0.2836 | 3.2728 |
| C(97) | -8.0629 | -4.298 | -4.387 | H(452) | 5.3635 | 10.3578 | 2.085 | C(97) | -8.4135 | -5.3543 | -4.2123 | H(452) | 12.2706 | 0.1885 | 2.016 |
| C(98) | -7.8205 | -4.9614 | -5.5315 | H(453) | 6.7684 | 10.3749 | 0.9627 | C(98) | -8.1317 | -6.0424 | -5.3328 | H(453) | 14.3822 | -1.0707 | 1.2724 |
| C(99) | -6.6951 | -5.6805 | -5.67 | H(454) | 6.9971 | 9.9425 | 2.7055 | C(99) | -6.96 | -6.6879 | -5.4537 | H(454) | 12.9521 | -2.1553 | 1.4294 |
| C(100) | -5.7712 | -5.7323 | -4.6951 | H(455) | 7.1192 | 6.8709 | 0.8236 | C(100) | -6.0258 | -6.6389 | -4.4882 | H(455) | 14.2167 | -2.143 | 2.7108 |
| C(101) | -6.0439 | -5.0306 | -3.5758 | C(456) | 10.1399 | 9.1553 | 1.4676 | C(101) | -6.3401 | -5.9212 | -3.3912 | H(456) | 11.497 | -0.3336 | 4.1142 |
| C(102) | -7.1924 | -4.3606 | -3.3647 | C(457) | 10.7819 | 9.3177 | 0.0976 | C(102) | -7.5339 | -5.3297 | -3.1959 | C(457) | 11.8223 | -4.0439 | 5.0777 |
| C(103) | -6.9945 | -3.8359 | 1.561 | O(458) | 9.9107 | 7.7851 | 1.7287 | C(103) | -7.3305 | -4.6132 | 1.7049 | C(458) | 13.1668 | -3.588 | 5.6252 |
| O(104) | -7.2958 | -5.1012 | 2.1693 | H(459) | 10.8079 | 9.5468 | 2.2673 | O(104) | -7.4733 | -5.8829 | 2.3546 | O(459) | 11.7628 | -3.807 | 3.6853 |
| C(105) | -4.4916 | -6.557 | -4.9189 | H(460) | 9.1652 | 9.685 | 1.5229 | C(105) | -4.6886 | -7.3703 | -4.6981 | H(460) | 11.6936 | -5.1363 | 5.2432 |
| O(106) | -3.4161 | -6.2666 | -4.0365 | H(461) | 11.7332 | 8.7417 | 0.0286 | O(106) | -3.6249 | -6.9862 | -3.8347 | H(461) | 10.9745 | -3.5013 | 5.5494 |
| O(107) | -4.5768 | -7.9529 | -4.5971 | H(462) | 11.0129 | 10.3883 | -0.1063 | O(107) | -4.6807 | -8.7589 | -4.3381 | H(462) | 13.2254 | -3.7575 | 6.7247 |
| O(108) | -5.5844 | -3.985 | 1.6002 | H(463) | 10.1026 | 8.9635 | -0.7096 | O(108) | -5.9124 | -4.5828 | 1.7329 | H(463) | 13.3303 | -2.5038 | 5.4446 |
| C(109) | -13.0883 | 2.0039 | -3.0437 | H(464) | 9.1874 | 7.4405 | 1.1935 | C(109) | -13.7017 | 0.7976 | -3.1758 | H(464) | 14.006 | -4.1428 | 5.1473 |
| C(110) | -12.7179 | 2.9192 | -1.8709 | C(465) | -0.8326 | -8.4358 | 4.3407 | C(110) | -13.1995 | 1.8814 | -2.2111 | H(465) | 11.5604 | -2.882 | 3.5233 |
| C(111) | -13.438 | 4.2749 | -1.9855 | C(466) | -0.4167 | -7.1132 | 4.969 | C(111) | -13.9564 | 3.2054 | -2.4139 | C(466) | 12.1961 | 6.6702 | 4.7771 |
| C(112) | -13.1671 | 5.2006 | -0.7876 | O(467) | -1.5566 | -8.1884 | 3.1525 | C(112) | -13.48 | 4.3161 | -1.462 | C(467) | 13.5078 | 6.5696 | 5.5423 |
| C(113) | -14.1112 | 4.936 | 0.397 | H(468) | 0.0556 | -9.0497 | 4.0755 | C(113) | -14.1209 | 4.2301 | -0.0669 | O(468) | 11.5924 | 5.3972 | 4.6581 |
| C(114) | -15.4894 | 5.599 | 0.231 | H(469) | -1.4882 | -9.0261 | 5.0187 | C(114) | -15.5356 | 4.8323 | -0.0171 | H(469) | 11.478 | 7.3363 | 5.3053 |
| C(115) | -16.4484 | 5.2973 | 1.3956 | H(470) | 0.2153 | -7.2808 | 5.8708 | C(115) | -16.1999 | 4.696 | 1.3637 | H(470) | 12.3761 | 7.0532 | 3.7478 |
| C(116) | -17.0805 | 3.8984 | 1.3178 | H(471) | -1.3077 | -6.5254 | 5.286 | C(116) | -16.7821 | 3.2973 | 1.6226 | H(471) | 13.3461 | 6.173 | 6.5702 |
| C(117) | -17.9688 | 3.6129 | 2.5354 | H(472) | 0.1703 | -6.495 | 4.2519 | C(117) | -17.3387 | 3.1751 | 3.0468 | H(472) | 13.9853 | 7.5715 | 5.6393 |
| C(118) | -6.2219 | 1.9665 | -5.4003 | H(473) | -1.7753 | -9.0211 | 2.7267 | C(118) | -6.9355 | 0.9586 | -5.6373 | H(473) | 14.2296 | 5.9053 | 5.0147 |
| C(119) | -5.5041 | 2.4485 | -4.13 | C(474) | -0.296 | -11.7279 | 2.4773 | C(119) | -6.1737 | 1.605 | -4.4688 | H(474) | 11.1633 | 5.3077 | 3.7946 |
| C(120) | -4.7023 | 1.3383 | -3.4272 | C(475) | -0.4607 | -12.6239 | 3.6963 | C(120) | -5.2303 | 0.6107 | -3.7769 | C(475) | 5.7948 | 6.6529 | 1.9535 |
| C(121) | -3.6748 | 1.8612 | -2.4115 | O(476) | -1.4035 | -10.8575 | 2.3881 | C(121) | -4.1797 | 1.2624 | -2.8694 | C(476) | 5.508 | 8.1048 | 2.3085 |
| C(122) | -4.287 | 2.6429 | -1.2401 | H(477) | 0.622 | -11.1046 | 2.56 | C(122) | -4.7515 | 1.9591 | -1.6284 | O(477) | 7.1898 | 6.4285 | 1.9683 |
| C(123) | -3.212 | 3.3952 | -0.442 | H(478) | -0.2565 | -12.3173 | 1.5346 | C(123) | -3.6328 | 2.6121 | -0.8022 | H(478) | 5.3255 | 5.9471 | 2.6728 |
| C(124) | -3.8074 | 4.4119 | 0.5428 | H(479) | -0.5486 | -12.0193 | 4.6282 | C(124) | -4.1802 | 3.4094 | 0.3898 | H(479) | 5.4266 | 6.4052 | 0.935 |
| C(125) | -2.7076 | 5.2083 | 1.2631 | H(480) | 0.4137 | -13.3049 | 3.8087 | C(125) | -3.0457 | 4.0686 | 1.1892 | H(480) | 4.4133 | 8.3102 | 2.3005 |
| C(126) | -3.291 | 6.2916 | 2.1785 | H(481) | -1.3731 | -13.2559 | 3.6097 | C(126) | -3.5798 | 4.9307 | 2.3393 | H(481) | 5.9878 | 8.7947 | 1.5775 |
| N(127) | -10.6953 | -4.6756 | -3.7862 | H(482) | -2.172 | -11.3475 | 2.0836 | N(127) | -11.0075 | -5.8532 | -3.6574 | H(482) | 5.8933 | 8.3516 | 3.3239 |
| C(128) | -11.1349 | -4.997 | -2.662 | C(483) | 13.8741 | 3.1684 | 5.4911 | C(128) | -11.5878 | -6.1273 | -2.5858 | H(483) | 7.3855 | 5.6223 | 1.4895 |
| S(129) | -11.684 | -5.437 | -1.2912 | C(484) | 15.0226 | 2.8893 | 6.4505 | S(129) | -12.3085 | -6.5144 | -1.2799 | C(484) | 9.5623 | 8.108 | 2.9421 |
| N(130) | -10.2887 | -2.9554 | -5.8993 | O(485) | 13.3933 | 1.9554 | 4.9474 | N(130) | -10.7055 | -4.1605 | -5.7649 | C(485) | 9.8313 | 8.6861 | 1.5605 |
| C(131) | -11.386 | -3.4378 | -6.2512 | H(486) | 13.022 | 3.6669 | 6.0029 | C(131) | -11.8007 | -4.6673 | -6.088 | O(486) | 9.7817 | 6.7116 | 2.9159 |
| S(132) | -12.7322 | -4.0266 | -6.7142 | H(487) | 14.2215 | 3.8088 | 4.6502 | S(132) | -13.1455 | -5.2867 | -6.5135 | H(487) | 10.2453 | 8.5564 | 3.696 |
| H(133) | -3.95 | 5.8456 | 2.958 | H(488) | 15.8697 | 2.3917 | 5.9261 | H(133) | -4.1852 | 4.3219 | 3.0491 | H(488) | 8.5145 | 8.2794 | 3.2694 |
| O(134) | -6.5685 | -13.4843 | 5.6099 | H(489) | 14.7021 | 2.2299 | 7.2885 | O(134) | -6.3562 | -13.9422 | 6.0983 | H(489) | 10.8767 | 8.4772 | 1.2365 |
| H(135) | -7.2319 | -13.7784 | 5.0094 | H(490) | 15.406 | 3.8375 | 6.8926 | H(135) | -7.0161 | -14.2788 | 5.5166 | H(490) | 9.6827 | 9.7899 | 1.554 |
| O(136) | -3.8536 | -16.0619 | -0.7184 | H(491) | 12.4368 | 1.9843 | 4.8547 | O(136) | -3.6091 | -16.6622 | -0.1593 | H(491) | 9.1452 | 8.2492 | 0.8018 |
| H(137) | -4.7938 | -16.0971 | -0.674 | C(492) | 9.0153 | 5.0685 | 6.2643 | H(137) | -4.5473 | -16.7292 | -0.11 | H(492) | 9.1249 | 6.2873 | 2.3513 |
| Ru(138) | 1.6343 | 3.8346 | -1.9569 | C(493) | 8.6913 | 4.4021 | 7.5937 | Ru(138) | 1.3507 | 3.3874 | -2.329 | C(493) | -0.8913 | -8.6109 | 4.4177 |
| C(139) | 1.5902 | 5.6699 | -1.0933 | O(494) | 9.6946 | 4.1538 | 5.4287 | C(139) | 1.367 | 5.2227 | -1.4677 | C(494) | -0.5035 | -7.202 | 4.8435 |
| C(140) | 2.5641 | 6.1504 | -0.3019 | H(495) | 8.0949 | 5.3949 | 5.7316 | C(140) | 2.4315 | 5.7278 | -0.822 | O(495) | -1.6349 | -8.5572 | 3.2181 |
| C(141) | 2.4721 | 7.3888 | 0.1995 | H(496) | 9.6767 | 5.9498 | 6.4126 | C(141) | 2.3365 | 6.9221 | -0.2239 | H(496) | 0.004 | -9.24 | 4.2265 |
| C(142) | 1.4147 | 8.1675 | -0.0733 | H(497) | 8.0262 | 3.5209 | 7.4522 | C(142) | 1.1831 | 7.6089 | -0.2343 | H(497) | -1.522 | -9.1129 | 5.1845 |
| C(143) | 0.4338 | 7.6673 | -0.8438 | H(498) | 8.1707 | 5.1123 | 8.2761 | C(143) | 0.133 | 7.0997 | -0.9022 | H(498) | 0.1506 | -7.2222 | 5.7448 |
| C(144) | 0.5127 | 6.4292 | -1.3683 | H(499) | 9.6193 | 4.0554 | 8.1036 | C(144) | 0.2171 | 5.9192 | -1.5454 | H(499) | -1.4066 | -6.6039 | 5.0998 |
| C(145) | 1.2945 | 2.0363 | -2.8291 | H(500) | 9.9575 | 4.5919 | 4.6135 | C(145) | 1.0145 | 1.5255 | -3.0584 | H(500) | 0.0478 | -6.6709 | 4.0338 |
| C(146) | 0.5783 | 1.1526 | -2.1063 | C(501) | 10.4704 | 1.0449 | 6.8095 | C(146) | 0.4179 | 0.6516 | -2.2226 | H(501) | -1.8223 | -9.451 | 2.9169 |
| C(147) | 0.4582 | -0.1015 | -2.5948 | C(502) | 11.6775 | 1.4069 | 7.6602 | C(147) | 0.3315 | -0.6417 | -2.6125 | C(502) | -0.2134 | -12.1324 | 3.0163 |
| C(148) | 1.1483 | -0.5265 | -3.6764 | O(503) | 10.5935 | 1.5573 | 5.4954 | C(148) | 0.9775 | -1.1052 | -3.7056 | C(503) | -0.4145 | -13.0016 | 4.2489 |
| C(149) | 1.839 | 0.3898 | -4.3721 | H(504) | 10.356 | -0.0596 | 6.7381 | C(149) | 1.5636 | -0.2016 | -4.5076 | O(504) | -1.3495 | -11.3152 | 2.8283 |
| C(150) | 1.8761 | 1.6696 | -3.983 | H(505) | 9.5496 | 1.4834 | 7.2526 | C(150) | 1.5395 | 1.1065 | -4.222 | H(505) | 0.6695 | -11.4673 | 3.1427 |
| C(151) | -2.3027 | 5.9002 | -3.5657 | H(506) | 11.8179 | 2.5103 | 7.7104 | C(151) | -2.7356 | 5.4049 | -3.5635 | H(506) | -0.0852 | -12.7451 | 2.0965 |
| C(152) | -1.9561 | 4.6954 | -4.0434 | H(507) | 12.6065 | 0.9447 | 7.26 | C(152) | -2.3958 | 4.226 | -4.1091 | H(507) | -0.6131 | -12.378 | 5.1507 |
| C(153) | -0.8584 | 4.0729 | -3.5945 | H(508) | 11.5485 | 1.0369 | 8.7032 | C(153) | -1.2667 | 3.6019 | -3.7481 | H(508) | 0.4904 | -13.6195 | 4.4499 |
| C(154) | -0.0649 | 4.6652 | -2.6883 | H(509) | 9.759 | 1.9717 | 5.2312 | C(154) | -0.4303 | 4.1862 | -2.8764 | H(509) | -1.2729 | -13.6972 | 4.1147 |
| C(155) | -0.3758 | 5.8889 | -2.2231 | C(510) | -5.1968 | -1.1247 | -0.3461 | C(155) | -0.728 | 5.3857 | -2.3439 | H(510) | -2.0935 | -11.8602 | 2.5582 |
| C(156) | -1.5034 | 6.4823 | -2.6564 | C(511) | -5.2939 | -0.5471 | 1.0579 | C(156) | -1.8955 | 5.9674 | -2.6771 | C(511) | 14.2968 | 2.97 | 6.3793 |
| C(157) | 0.5687 | 2.8323 | -0.5543 | O(512) | -6.4553 | -1.0877 | -0.9925 | C(157) | 0.5091 | 2.4109 | -0.7636 | C(512) | 15.761 | 3.218 | 6.048 |
| C(158) | 0.4088 | 3.2972 | 0.6961 | H(513) | -4.4745 | -0.5491 | -0.9616 | C(158) | 0.4657 | 2.9179 | 0.4809 | O(513) | 13.8357 | 1.8294 | 5.6864 |
| C(159) | -0.0933 | 2.4942 | 1.6428 | H(514) | -4.8647 | -2.1842 | -0.3205 | C(159) | 0.0911 | 2.1496 | 1.5135 | H(514) | 14.1421 | 2.7962 | 7.4669 |
| C(160) | -0.5349 | 1.2608 | 1.3474 | H(515) | -5.6067 | 0.5201 | 1.0415 | C(160) | -0.3306 | 0.8868 | 1.3343 | H(515) | 13.6664 | 3.8252 | 6.0584 |
| C(161) | -0.4674 | 0.8647 | 0.0567 | H(516) | -4.3077 | -0.6022 | 1.5741 | C(161) | -0.3544 | 0.4405 | 0.0598 | H(516) | 15.8999 | 3.4005 | 4.9577 |
| C(162) | 0.1722 | 1.5882 | -0.8878 | H(517) | -6.0336 | -1.1033 | 1.6747 | C(162) | 0.1315 | 1.1379 | -0.9895 | H(517) | 16.3905 | 2.3461 | 6.3382 |
| C(163) | 1.2642 | -1.9786 | -4.16 | H(518) | -6.472 | -1.7232 | -1.7168 | C(163) | 1.1979 | -2.5797 | -4.0697 | H(518) | 16.1421 | 4.1129 | 6.5913 |
| O(164) | 0.591 | -2.9751 | -3.3995 | C(519) | -8.0624 | 1.4423 | -0.8712 | O(164) | 0.5278 | -3.5681 | -3.2987 | H(519) | 12.8784 | 1.7689 | 5.7654 |
| C(165) | -1.0033 | 0.4037 | 2.5332 | C(520) | -8.9092 | 2.705 | -0.9173 | C(165) | -0.6909 | 0.081 | 2.5986 | C(520) | 10.7692 | 3.8775 | 7.279 |
| O(166) | 0.0981 | 0.0247 | 3.3745 | O(521) | -8.8042 | 0.3395 | -1.3513 | O(166) | 0.4555 | -0.4627 | 3.2714 | C(521) | 10.5753 | 2.9983 | 8.5055 |
| O(167) | -1.4426 | -0.9238 | 2.2603 | H(522) | -7.1658 | 1.5507 | -1.5192 | O(167) | -1.3802 | -1.1572 | 2.4393 | O(522) | 11.124 | 3.0745 | 6.1726 |
| O(168) | 2.5851 | -2.5084 | -3.9591 | H(523) | -7.7449 | 1.2076 | 0.1685 | O(168) | 2.5272 | -3.0202 | -3.7483 | H(523) | 9.8343 | 4.4199 | 7.0148 |
| C(169) | -3.5864 | 6.5592 | -4.0252 | H(524) | -9.83 | 2.5947 | -0.3016 | C(169) | -4.0458 | 6.0609 | -3.9548 | H(524) | 11.5865 | 4.6153 | 7.4369 |
| C(170) | -4.7057 | 6.1974 | -3.0367 | H(525) | -9.2118 | 2.944 | -1.9614 | C(170) | -5.1872 | 5.4257 | -3.1489 | H(525) | 9.7656 | 2.2517 | 8.3415 |
| C(171) | -6.1156 | 6.4173 | -3.6032 | H(526) | -8.34 | 3.578 | -0.5237 | C(171) | -6.5903 | 5.7666 | -3.6714 | H(526) | 10.2968 | 3.6119 | 9.3927 |
| C(172) | -7.1974 | 5.9219 | -2.6279 | H(527) | -8.2424 | -0.4371 | -1.4526 | C(172) | -7.6803 | 5.1466 | -2.7786 | H(527) | 11.5107 | 2.4468 | 8.7525 |
| C(173) | -8.6286 | 6.0059 | -3.1863 | C(528) | -3.849 | -2.356 | -3.3321 | C(173) | -9.0908 | 5.2032 | -3.3899 | H(528) | 11.2204 | 3.632 | 5.3927 |
| C(174) | -8.8713 | 5.0703 | -4.3822 | C(529) | -3.4204 | -2.4825 | -4.7849 | C(174) | -9.3035 | 4.1642 | -4.5049 | C(529) | 11.4683 | -0.445 | 7.1975 |
| C(175) | -10.3406 | 5.065 | -4.8309 | O(530) | -5.2402 | -2.1288 | -3.2418 | C(175) | -10.7555 | 4.1441 | -5.0077 | C(530) | 12.8523 | -0.359 | 7.8228 |
| C(176) | -10.5628 | 4.1869 | -6.0728 | H(531) | -3.6479 | -3.2991 | -2.7848 | C(176) | -10.9557 | 3.1446 | -6.1582 | O(531) | 11.4076 | 0.3163 | 6.0065 |
| C(177) | -11.9981 | 4.3059 | -6.6014 | H(532) | -3.3188 | -1.521 | -2.8231 | C(177) | -12.3984 | 3.1607 | -6.6796 | H(532) | 11.2195 | -1.4991 | 6.9409 |
| C(178) | 1.3719 | 9.5931 | 0.4353 | H(533) | -2.334 | -2.723 | -4.8531 | C(178) | 1.1115 | 8.9482 | 0.4692 | H(533) | 10.693 | -0.0518 | 7.8907 |
| C(179) | 1.9325 | 10.5324 | -0.6473 | H(534) | -3.5899 | -1.5321 | -5.3378 | C(179) | 1.5803 | 10.0585 | -0.4809 | H(534) | 13.0991 | 0.6869 | 8.1113 |
| C(180) | 2.1155 | 11.984 | -0.1729 | H(535) | -3.9845 | -3.2931 | -5.3005 | C(180) | 1.9235 | 11.3836 | 0.2188 | H(535) | 13.6374 | -0.7208 | 7.1224 |
| C(181) | 3.3682 | 12.1696 | 0.7 | H(536) | -5.7214 | -2.6822 | -3.8652 | C(181) | 3.2534 | 11.3146 | 0.9904 | H(536) | 12.9087 | -0.9839 | 8.7434 |
| C(182) | 3.5555 | 13.6266 | 1.1521 | C(537) | -16.7221 | 0.9503 | -1.0497 | C(182) | 3.7243 | 12.6992 | 1.4624 | H(537) | 10.824 | 1.0742 | 6.1106 |
| C(183) | 4.8562 | 13.8501 | 1.946 | C(538) | -17.065 | 1.8983 | -2.1898 | C(183) | 5.0415 | 12.6559 | 2.2579 | C(538) | -7.4395 | -1.0623 | 3.0758 |
| C(184) | 6.108 | 13.897 | 1.0536 | O(539) | -15.3893 | 1.1625 | -0.6309 | C(184) | 6.2524 | 12.2194 | 1.4172 | C(539) | -8.3246 | -1.2349 | 4.3025 |
| C(185) | 7.4032 | 14.2162 | 1.8199 | H(540) | -17.3838 | 1.127 | -0.1732 | C(185) | 7.5973 | 12.3551 | 2.1511 | O(540) | -8.189 | -1.3721 | 1.9191 |
| C(186) | 7.9057 | 13.052 | 2.6831 | H(541) | -16.8088 | -0.114 | -1.361 | C(186) | 7.7044 | 11.4585 | 3.391 | H(541) | -7.0815 | -0.0129 | 2.9796 |
| N(187) | 3.2057 | 3.2178 | -1.0168 | H(542) | -18.1355 | 1.7946 | -2.4805 | N(187) | 3.0595 | 2.814 | -1.6343 | H(542) | -6.5638 | -1.7486 | 3.1067 |
| C(188) | 3.6812 | 2.0709 | -1.1593 | H(543) | -16.4525 | 1.683 | -3.0937 | C(188) | 4.001 | 2.4739 | -2.3825 | H(543) | -9.2058 | -0.5543 | 4.2577 |
| S(189) | 4.3016 | 0.6684 | -1.3104 | H(544) | -16.8892 | 2.9588 | -1.8976 | S(189) | 5.1833 | 2.0613 | -3.2811 | H(544) | -7.7597 | -1.0044 | 5.2347 |
| N(190) | 2.6267 | 4.7081 | -3.3645 | H(545) | -15.1382 | 0.4642 | -0.0183 | N(190) | 2.0145 | 4.1966 | -3.9523 | H(545) | -8.7008 | -2.2809 | 4.3801 |
| C(191) | 3.8022 | 4.3919 | -3.6456 | C(546) | -13.8706 | 2.2468 | 2.9854 | C(191) | 3.0842 | 4.8409 | -3.9947 | H(546) | -7.624 | -1.4619 | 1.1449 |
| S(192) | 5.2455 | 4.0142 | -4.0308 | C(547) | -12.4604 | 2.6904 | 3.3484 | S(192) | 4.4045 | 5.6314 | -4.0775 | C(547) | -8.7044 | 1.0577 | -1.0116 |
| H(193) | 3.4695 | 5.5807 | -0.0422 | O(548) | -13.8642 | 1.6456 | 1.7057 | H(193) | 3.4001 | 5.2047 | -0.747 | C(548) | -9.552 | 2.3131 | -0.8674 |
| H(194) | 3.2857 | 7.7776 | 0.8301 | H(549) | -14.2652 | 1.5031 | 3.7128 | H(194) | 3.2157 | 7.3347 | 0.2969 | O(549) | -9.4536 | 0.0584 | -1.6719 |
| H(195) | -0.404 | 8.3431 | -1.0672 | H(550) | -14.5636 | 3.1157 | 2.9469 | H(195) | -0.7795 | 7.7124 | -0.9307 | H(550) | -7.7991 | 1.2514 | -1.6277 |
| H(196) | -0.134 | -0.8434 | -2.0458 | H(551) | -12.4525 | 3.222 | 4.3274 | H(196) | -0.1376 | -1.3829 | -1.9563 | H(551) | -8.3945 | 0.6631 | -0.0191 |
| H(197) | 2.3959 | 0.0856 | -5.2742 | H(552) | -12.0475 | 3.3833 | 2.5794 | H(197) | 2.0949 | -0.5392 | -5.4133 | H(552) | -10.4687 | 2.119 | -0.2659 |
| H(198) | 2.4518 | 2.3627 | -4.6164 | H(553) | -11.774 | 1.8178 | 3.4331 | H(198) | 2.0356 | 1.7848 | -4.9365 | H(553) | -9.874 | 2.6856 | -1.8652 |
| H(199) | -2.596 | 4.1946 | -4.7877 | H(554) | -14.7653 | 1.5797 | 1.3732 | H(199) | -3.066 | 3.7517 | -4.8449 | H(554) | -8.9783 | 3.1267 | -0.3674 |
| H(200) | -0.6561 | 3.0732 | -4.002 | C(555) | -15.6866 | -1.0745 | -4.9623 | H(200) | -1.0641 | 2.6187 | -4.198 | H(555) | -8.8817 | -0.6311 | -2.0285 |
| H(201) | 0.7296 | 4.304 | 1.0131 | C(556) | -17.0065 | -1.5233 | -4.3516 | H(201) | 0.774 | 3.9491 | 0.7214 | C(556) | -3.7508 | -3.0643 | -5.5398 |
| H(202) | -1.7822 | 0.9416 | 3.126 | O(557) | -14.6868 | -2.0311 | -4.6736 | H(202) | -1.2738 | 0.7266 | 3.3017 | C(557) | -2.3782 | -3.7002 | -5.3803 |
| H(203) | -3.4695 | 7.6647 | -4.0999 | H(558) | -15.7537 | -0.977 | -6.0687 | H(203) | -4.0194 | 7.1617 | -3.7858 | O(558) | -4.7191 | -3.8454 | -4.8713 |
| H(204) | -3.8438 | 6.2019 | -5.0492 | H(559) | -15.3575 | -0.1017 | -4.5342 | H(204) | -4.2215 | 5.9154 | -5.046 | H(559) | -3.7741 | -2.0488 | -5.0882 |
| H(205) | -4.6083 | 5.1159 | -2.7904 | H(560) | -17.8054 | -0.7681 | -4.5335 | H(205) | -5.0736 | 4.3202 | -3.2155 | H(560) | -4.0504 | -2.9966 | -6.6092 |
| H(206) | -4.5731 | 6.7616 | -2.0837 | H(561) | -16.9104 | -1.6601 | -3.2502 | H(206) | -5.0902 | 5.7118 | -2.075 | H(561) | -1.6045 | -3.127 | -5.9396 |
| H(207) | -6.2692 | 7.4982 | -3.8305 | H(562) | -17.3407 | -2.4916 | -4.7895 | H(207) | -6.7229 | 6.8732 | -3.7144 | H(562) | -2.3727 | -4.7487 | -5.7563 |
| H(208) | -6.1912 | 5.8558 | -4.563 | H(563) | -13.8212 | -1.6523 | -4.8601 | H(208) | -6.6813 | 5.3799 | -4.713 | H(563) | -2.0728 | -3.713 | -4.3108 |
| H(209) | -6.9899 | 4.865 | -2.3388 | C(564) | 0.0175 | 7.8524 | -5.4048 | H(209) | -7.4343 | 4.0842 | -2.5475 | H(564) | -5.5301 | -3.3346 | -4.7631 |
| H(210) | -7.1345 | 6.5257 | -1.6905 | C(565) | -0.839 | 7.6304 | -6.6435 | H(210) | -7.6791 | 5.6874 | -1.8012 | C(565) | -16.9859 | 0.1367 | -0.5119 |
| H(211) | -9.3436 | 5.7333 | -2.3721 | O(566) | 0.4995 | 6.6086 | -4.9405 | H(211) | -9.8382 | 5.0107 | -2.5827 | C(566) | -17.4992 | 0.9916 | -1.6616 |
| H(212) | -8.8523 | 7.0589 | -3.479 | H(567) | 0.893 | 8.5005 | -5.6256 | H(212) | -9.2923 | 6.23 | -3.7753 | O(567) | -15.5934 | 0.3238 | -0.3611 |
| H(213) | -8.2401 | 5.3856 | -5.2449 | H(568) | -0.5724 | 8.3134 | -4.5822 | H(213) | -8.6224 | 4.3805 | -5.3601 | H(568) | -17.4711 | 0.4209 | 0.4481 |
| H(214) | -8.5669 | 4.0346 | -4.108 | H(569) | -1.6871 | 6.9411 | -6.4276 | H(214) | -9.0433 | 3.1487 | -4.125 | H(569) | -17.1602 | -0.946 | -0.6991 |
| H(215) | -10.9827 | 4.695 | -3.9976 | H(570) | -0.2399 | 7.1865 | -7.4707 | H(215) | -11.4349 | 3.8767 | -4.1643 | H(570) | -18.6043 | 0.8937 | -1.7634 |
| H(216) | -10.6542 | 6.1115 | -5.0584 | H(571) | -1.2651 | 8.5928 | -7.0086 | H(216) | -11.038 | 5.1664 | -5.354 | H(571) | -17.0446 | 0.6822 | -2.6297 |
| H(217) | -9.8565 | 4.4926 | -6.8813 | H(572) | 0.8132 | 6.6871 | -4.0327 | H(217) | -10.2616 | 3.3947 | -6.9957 | H(572) | -17.2646 | 2.0679 | -1.4967 |
| H(218) | -10.3404 | 3.1216 | -5.8266 | C(573) | -1.6561 | 1.1026 | -5.9649 | H(218) | -10.7002 | 2.1144 | -5.8151 | H(573) | -15.2558 | -0.3311 | 0.2576 |
| H(219) | -12.7395 | 3.9935 | -5.832 | C(574) | -0.6027 | 0.5915 | -6.9362 | H(219) | -13.1175 | 2.8691 | -5.8811 | C(574) | -13.3752 | 1.7655 | 2.7959 |
| H(220) | -12.2332 | 5.3549 | -6.8929 | O(575) | -1.3163 | 0.7023 | -4.6532 | H(220) | -12.6832 | 4.1741 | -7.0435 | C(575) | -11.882 | 2.0126 | 2.9614 |
| H(221) | -12.1467 | 3.6673 | -7.5022 | H(576) | -1.7184 | 2.2122 | -5.9824 | H(221) | -12.5249 | 2.4509 | -7.5291 | O(576) | -13.6104 | 1.0614 | 1.5925 |
| H(222) | 0.3287 | 9.8831 | 0.6995 | H(577) | -2.6583 | 0.6793 | -6.2004 | H(222) | 0.0787 | 9.1587 | 0.8309 | H(577) | -13.7825 | 1.1578 | 3.634 |
| H(223) | 1.9654 | 9.6765 | 1.3737 | H(578) | -0.8578 | 0.8722 | -7.9836 | H(223) | 1.751 | 8.9102 | 1.3802 | H(578) | -13.9341 | 2.7256 | 2.7412 |
| H(224) | 2.9134 | 10.1479 | -1.0151 | H(579) | -0.5159 | -0.5182 | -6.8873 | H(224) | 2.4861 | 9.7112 | -1.0298 | H(579) | -11.68 | 2.6169 | 3.8754 |
| H(225) | 1.237 | 10.5161 | -1.5215 | H(580) | 0.3951 | 1.0266 | -6.7042 | H(225) | 0.7849 | 10.2296 | -1.2459 | H(580) | -11.4688 | 2.5668 | 2.0875 |
| H(226) | 2.2143 | 12.6437 | -1.0689 | H(581) | -0.4858 | 1.1123 | -4.3746 | H(226) | 2.0102 | 12.1782 | -0.5613 | H(581) | -11.3231 | 1.0547 | 3.0628 |
| H(227) | 1.2048 | 12.3173 | 0.3772 | C(582) | 3.6414 | 8.1206 | -3.4983 | H(227) | 1.0927 | 11.6845 | 0.8986 | H(582) | -14.5572 | 0.9659 | 1.4465 |
| H(228) | 3.2983 | 11.5199 | 1.6028 | C(583) | 3.8244 | 8.0119 | -5.0046 | H(228) | 3.1498 | 10.6424 | 1.8733 | C(583) | -16.2076 | -2.2228 | -4.776 |
| H(229) | 4.2622 | 11.8383 | 0.1235 | O(584) | 2.2981 | 7.859 | -3.1548 | H(229) | 4.0301 | 10.8681 | 0.3283 | C(584) | -17.2804 | -2.4817 | -3.7268 |
| H(230) | 3.5353 | 14.3095 | 0.2705 | H(585) | 4.2623 | 7.367 | -2.9685 | H(230) | 3.8471 | 13.3766 | 0.5841 | O(585) | -15.1791 | -3.1847 | -4.6436 |
| H(231) | 2.6844 | 13.9043 | 1.794 | H(586) | 3.8952 | 9.1349 | -3.1202 | H(231) | 2.9269 | 13.1438 | 2.1058 | H(586) | -16.6123 | -2.295 | -5.81 |
| H(232) | 4.7787 | 14.8246 | 2.4867 | H(587) | 3.482 | 7.0208 | -5.3806 | H(232) | 5.2429 | 13.6753 | 2.6672 | H(587) | -15.7528 | -1.2166 | -4.6384 |
| H(233) | 4.9563 | 13.0566 | 2.7218 | H(588) | 4.8968 | 8.1266 | -5.2832 | H(233) | 4.9135 | 11.9718 | 3.1291 | H(588) | -18.0953 | -1.725 | -3.796 |
| H(234) | 6.2358 | 12.9355 | 0.5056 | H(589) | 3.2501 | 8.8039 | -5.5361 | H(234) | 6.1326 | 11.159 | 1.0976 | H(589) | -16.8547 | -2.4293 | -2.6985 |
| H(235) | 5.9533 | 14.6947 | 0.2871 | H(590) | 2.2537 | 7.6679 | -2.2119 | H(235) | 6.29 | 12.8384 | 0.4885 | H(590) | -17.7343 | -3.4903 | -3.8594 |
| H(236) | 8.2049 | 14.4714 | 1.0839 | C(591) | 1.6996 | 4.4591 | -6.8896 | H(236) | 8.4183 | 12.0823 | 1.4435 | H(591) | -14.3398 | -2.7963 | -4.9101 |
| H(237) | 7.2511 | 15.1204 | 2.4555 | C(592) | 2.1484 | 3.292 | -7.7577 | H(237) | 7.7637 | 13.4189 | 2.4439 | C(592) | -0.8574 | 7.1477 | -5.7935 |
| H(238) | 8.838 | 13.3322 | 3.2253 | O(593) | 0.6423 | 4.0435 | -6.0501 | H(238) | 8.7349 | 11.4822 | 3.8144 | C(593) | -1.6891 | 6.6538 | -6.9692 |
| H(239) | 7.1558 | 12.7476 | 3.4467 | H(594) | 2.5269 | 4.818 | -6.2387 | H(239) | 7.0089 | 11.7835 | 4.1968 | O(594) | -0.1228 | 6.0734 | -5.2435 |
| Ru(240) | 7.694 | 3.2957 | 1.9761 | H(595) | 1.3283 | 5.3062 | -7.5073 | Ru(240) | 8.7329 | 2.4948 | 2.9549 | H(595) | -0.1371 | 7.9298 | -6.1132 |
| C(241) | 7.5841 | 4.6357 | 0.4587 | H(596) | 2.9776 | 3.599 | -8.4355 | C(241) | 8.435 | 3.6149 | 1.2909 | H(596) | -1.5008 | 7.5632 | -4.9875 |
| C(242) | 6.4494 | 4.9232 | -0.2003 | H(597) | 1.3103 | 2.9181 | -8.3894 | C(242) | 7.3142 | 3.5962 | 0.5456 | H(597) | -2.3396 | 5.799 | -6.6762 |
| C(243) | 6.4586 | 5.8447 | -1.1722 | H(598) | 2.5161 | 2.4449 | -7.1347 | C(243) | 7.1752 | 4.4585 | -0.472 | H(598) | -1.0393 | 6.3147 | -7.8069 |
| C(244) | 7.5919 | 6.481 | -1.5075 | H(599) | 0.4034 | 4.7514 | -5.4428 | C(244) | 8.1362 | 5.3563 | -0.7446 | H(599) | -2.3455 | 7.4656 | -7.3582 |
| C(245) | 8.7193 | 6.1729 | -0.8434 | C(600) | 14.0429 | -5.0234 | 3.3779 | C(245) | 9.2703 | 5.3246 | -0.0212 | H(600) | 0.1734 | 6.2865 | -4.3499 |
| C(246) | 8.7328 | 5.2636 | 0.1488 | C(601) | 15.1025 | -3.9319 | 3.3671 | C(246) | 9.4527 | 4.4359 | 0.9726 | C(601) | -1.9559 | 0.4427 | -5.9822 |
| C(247) | 8.5168 | 1.6677 | 1.0933 | O(602) | 13.1147 | -4.7872 | 2.3388 | C(247) | 9.3527 | 0.8715 | 1.9095 | C(602) | -0.8786 | 0.0484 | -6.9807 |
| C(248) | 8.7962 | 0.6351 | 1.9108 | H(603) | 14.499 | -6.0245 | 3.2061 | C(248) | 9.4355 | -0.2747 | 2.6097 | O(603) | -1.5715 | 0.0314 | -4.6857 |
| C(249) | 9.2382 | -0.4963 | 1.3213 | H(604) | 13.4772 | -5.0416 | 4.3356 | C(249) | 9.6717 | -1.4102 | 1.9173 | H(604) | -2.1131 | 1.5429 | -5.9652 |
| C(250) | 9.2372 | -0.6878 | -0.0167 | H(605) | 15.8322 | -4.0742 | 4.1967 | C(250) | 9.6537 | -1.4848 | 0.5682 | H(605) | -2.9215 | -0.0539 | -6.2253 |
| C(251) | 8.9396 | 0.376 | -0.7823 | H(606) | 14.6383 | -2.9287 | 3.4906 | C(251) | 9.6088 | -0.3092 | -0.0811 | H(606) | -1.1732 | 0.3326 | -8.0168 |
| C(252) | 8.6286 | 1.56 | -0.2408 | H(607) | 15.6681 | -3.9284 | 2.4073 | C(252) | 9.5081 | 0.8557 | 0.5738 | H(607) | -0.6932 | -1.0497 | -6.9636 |
| C(253) | 12.0911 | 4.929 | 1.4414 | H(608) | 12.4492 | -4.159 | 2.6347 | C(253) | 12.8941 | 4.5048 | 2.0908 | H(608) | 0.0808 | 0.5615 | -6.7503 |
| C(254) | 11.849 | 4.0119 | 2.3905 | C(609) | 15.9594 | 0.1145 | 3.6783 | C(254) | 12.8334 | 3.5 | 2.9792 | H(609) | -0.7609 | 0.4782 | -4.4076 |
| C(255) | 10.6107 | 3.544 | 2.594 | C(610) | 15.9755 | 1.2238 | 2.6356 | C(255) | 11.6812 | 2.8607 | 3.2182 | C(610) | 2.3739 | 8.6676 | -3.8154 |
| C(256) | 9.5913 | 3.9987 | 1.8471 | O(611) | 16.5891 | -1.0324 | 3.1442 | C(256) | 10.5756 | 3.2416 | 2.5607 | C(611) | 2.6027 | 8.8285 | -5.3111 |
| C(257) | 9.8082 | 4.9074 | 0.8787 | H(612) | 16.5161 | 0.4129 | 4.5944 | C(257) | 10.6054 | 4.2227 | 1.6411 | O(612) | 1.5024 | 7.5855 | -3.5575 |
| C(258) | 11.0609 | 5.3613 | 0.6951 | H(613) | 14.9218 | -0.1739 | 3.9571 | C(258) | 11.7758 | 4.851 | 1.4288 | H(613) | 3.3266 | 8.4463 | -3.2869 |
| C(259) | 7.9345 | 2.0058 | 3.5202 | H(614) | 15.4576 | 2.1362 | 3.009 | C(259) | 8.9359 | 1.0767 | 4.3885 | H(614) | 1.9196 | 9.5851 | -3.382 |
| C(260) | 7.4027 | 2.237 | 4.7308 | H(615) | 15.4658 | 0.8968 | 1.701 | C(260) | 8.5568 | 1.2844 | 5.6611 | H(615) | 2.9297 | 7.8722 | -5.7789 |
| C(261) | 7.3355 | 1.2453 | 5.6248 | H(616) | 17.0184 | 1.512 | 2.371 | C(261) | 8.2713 | 0.2398 | 6.4469 | H(616) | 3.3881 | 9.5932 | -5.51 |
| C(262) | 7.8755 | 0.0415 | 5.3768 | H(617) | 15.9948 | -1.4465 | 2.5131 | C(262) | 8.4559 | -1.019 | 6.0192 | H(617) | 1.6772 | 9.1691 | -5.8256 |
| C(263) | 8.5246 | -0.1187 | 4.2009 | C(618 | 2.3775 | -7.8687 | 0.4532 | C(263) | 9.0211 | -1.1908 | 4.8045 | H(618) | 1.5893 | 7.3366 | -2.6295 |
| C(264) | 8.4807 | 0.811 | 3.2197 | C(619) | 1.1313 | -8.1383 | 1.2807 | C(264) | 9.1769 | -0.1685 | 3.9358 | C(619) | 1.1122 | 3.9168 | -7.273 |
| C(265) | 9.4465 | -2.0173 | -0.7642 | O(620) | 3.3318 | -7.2003 | 1.2483 | C(265) | 9.6245 | -2.7676 | -0.2912 | C(620) | 1.5502 | 2.7456 | -8.141 |
| O(266) | 10.1119 | -3.108 | -0.1347 | H(621) | 2.8391 | -8.8119 | 0.0871 | O(266) | 10.1606 | -3.9767 | 0.2352 | O(621) | 0.1237 | 3.4882 | -6.3579 |
| C(267) | 7.6004 | -1.0285 | 6.4521 | H(622) | 2.163 | -7.2141 | -0.417 | C(267) | 7.9284 | -2.1293 | 6.9388 | H(622) | 1.9667 | 4.3232 | -6.6891 |
| O(268) | 8.0536 | -2.3665 | 6.2429 | H(623) | 0.7336 | -7.1934 | 1.7157 | O(268) | 8.076 | -3.4789 | 6.5125 | H(623) | 0.6758 | 4.7342 | -7.8873 |
| O(269) | 6.2006 | -1.3493 | 6.4854 | H(624) | 1.3634 | -8.8316 | 2.1187 | O(269) | 6.4916 | -2.1441 | 6.9577 | H(624) | 2.3157 | 3.0678 | -8.8836 |
| O(270) | 8.2032 | -2.6931 | -0.9457 | H(625) | 0.3277 | -8.6075 | 0.668 | O(270) | 8.298 | -3.2731 | -0.483 | H(625) | 0.6878 | 2.3192 | -8.7027 |
| C(271) | 13.4938 | 5.4377 | 1.1776 | H(626) | 4.0693 | -6.9338 | 0.6891 | C(271) | 14.2084 | 5.201 | 1.804 | H(626) | 1.9993 | 1.9344 | -7.5235 |
| C(272) | 14.0632 | 4.7998 | -0.1021 | C(627) | 4.5271 | -7.4406 | 4.6318 | C(272) | 14.8388 | 4.685 | 0.4989 | H(627) | -0.0253 | 4.1672 | -5.691 |
| C(273) | 14.3244 | 3.2914 | 0.0571 | C(628) | 3.157 | -8.0841 | 4.482 | C(273) | 15.2545 | 3.2019 | 0.5614 | C(628) | 14.0043 | -5.566 | 2.0859 |
| C(274) | 14.0484 | 2.4596 | -1.2064 | O(629) | 5.0436 | -7.1548 | 3.3484 | C(274) | 14.743 | 2.3474 | -0.6113 | C(629) | 13.4931 | -5.0851 | 0.736 |
| C(275) | 12.5459 | 2.3085 | -1.5099 | H(630) | 4.4504 | -6.4846 | 5.1935 | C(275) | 13.2155 | 2.1536 | -0.6152 | O(630) | 12.9441 | -6.1877 | 2.7818 |
| C(276) | 12.2413 | 1.3588 | -2.6818 | H(631) | 5.247 | -8.1136 | 5.1484 | C(276) | 12.7283 | 1.1943 | -1.7163 | H(631) | 14.8204 | -6.3135 | 1.9666 |
| C(277) | 12.5469 | -0.1163 | -2.3737 | H(632) | 2.7194 | -8.3303 | 5.4762 | C(277) | 13.0306 | -0.2809 | -1.4093 | H(632) | 14.3685 | -4.7195 | 2.709 |
| C(278) | 12.1514 | -1.048 | -3.5302 | H(633) | 3.2178 | -9.0249 | 3.8887 | C(278) | 12.5761 | -1.2271 | -2.5307 | H(633) | 14.2907 | -4.5473 | 0.1744 |
| C(279) | 12.5229 | -2.5081 | -3.2352 | H(634) | 2.4549 | -7.3917 | 3.9641 | C(279) | 12.9149 | -2.6872 | -2.1999 | H(634) | 12.6373 | -4.3873 | 0.8698 |
| C(280) | 7.5732 | 7.5459 | -2.5856 | H(635) | 4.3418 | -6.7885 | 2.8032 | C(280) | 7.9405 | 6.3592 | -1.864 | H(635) | 13.141 | -5.9356 | 0.1088 |
| C(281) | 7.0256 | 8.8529 | -1.995 | C(636) | 6.0619 | -9.1099 | 0.9571 | C(281) | 6.9406 | 7.4464 | -1.4416 | H(636) | 12.27 | -5.533 | 2.9892 |
| C(282) | 6.8911 | 9.9862 | -3.0211 | C(637) | 6.0134 | -9.6138 | -0.4782 | C(282) | 6.7835 | 8.5568 | -2.4902 | C(637) | 15.9199 | -0.691 | 5.0242 |
| C(283) | 6.2517 | 11.2217 | -2.3672 | O(638) | 6.0882 | -7.6984 | 0.9526 | C(283) | 5.8316 | 9.6548 | -1.9897 | C(638) | 16.5024 | 0.0869 | 3.8517 |
| C(284) | 5.9139 | 12.3239 | -3.3821 | H(639) | 5.167 | -9.4327 | 1.5342 | C(284) | 5.5952 | 10.7413 | -3.0501 | O(639) | 16.2762 | -2.0544 | 4.9169 |
| C(285) | 5.3029 | 13.5467 | -2.6803 | H(640) | 6.978 | -9.4592 | 1.4828 | C(285) | 4.5739 | 11.7837 | -2.5718 | H(640) | 16.306 | -0.3076 | 5.9952 |
| C(286) | 4.8616 | 14.639 | -3.6653 | H(641) | 6.0152 | -10.7274 | -0.508 | C(286) | 4.3045 | 12.8709 | -3.6222 | H(641) | 14.8099 | -0.6457 | 5.0307 |
| C(287) | 4.244 | 15.8405 | -2.9315 | H(642) | 6.8937 | -9.2519 | -1.0576 | C(287) | 3.2519 | 13.878 | -3.1319 | H(642) | 16.1593 | 1.1464 | 3.8647 |
| C(288) | 3.7997 | 16.9381 | -3.907 | H(643) | 5.0931 | -9.2603 | -0.9967 | C(288) | 2.9854 | 14.9755 | -4.1702 | H(643) | 16.1909 | -0.3536 | 2.8781 |
| N(289) | 6.0469 | 2.4421 | 1.4356 | H(644) | 6.228 | -7.3742 | 1.8459 | N(289) | 6.8109 | 2.4527 | 3.154 | H(644) | 17.6158 | 0.0865 | 3.8847 |
| C(290) | 5.2066 | 2.0019 | 2.2484 | C(645) | -13.8724 | -2.5897 | -0.3176 | C(290) | 6.1875 | 1.669 | 3.9021 | H(645) | 15.6774 | -2.4837 | 4.3003 |
| S(291) | 4.1379 | 1.4524 | 3.2126 | C(646) | -14.4629 | -3.4448 | 0.7939 | S(291) | 5.3531 | 0.7394 | 4.8066 | C(646) | 2.1926 | -8.537 | 0.4144 |
| N(292) | 7.0902 | 4.787 | 3.0438 | O(647) | -14.1219 | -1.2202 | -0.072 | N(292) | 8.84 | 3.9641 | 4.2067 | C(647) | 0.9212 | -8.7888 | 1.2088 |
| C(293) | 5.872 | 5.0273 | 3.1858 | H(648) | -12.7691 | -2.7187 | -0.3629 | C(293) | 7.8447 | 4.5473 | 4.6853 | O(648) | 3.1105 | -7.8251 | 1.2147 |
| S(294) | 4.3735 | 5.3366 | 3.3698 | H(649) | -14.3148 | -2.8481 | -1.3054 | S(294) | 6.6516 | 5.2807 | 5.3272 | H(649) | 2.674 | -9.4895 | 0.103 |
| H(295) | 5.4832 | 4.4494 | 0.0331 | H(650) | -14.1823 | -4.5147 | 0.6619 | H(295) | 6.469 | 2.9129 | 0.7336 | H(650) | 2.0046 | -7.9216 | -0.4897 |
| H(296) | 5.5163 | 6.0892 | -1.6853 | H(651) | -15.5742 | -3.3932 | 0.7931 | H(296) | 6.2455 | 4.4513 | -1.0654 | H(651) | 0.514 | -7.8347 | 1.6135 |
| H(297) | 9.6277 | 6.7157 | -1.1373 | H(652) | -14.0953 | -3.1172 | 1.7933 | H(297) | 10.0623 | 6.0333 | -0.3024 | H(652) | 1.1248 | -9.4625 | 2.0689 |
| H(298) | 9.5267 | -1.3247 | 1.9692 | H(653) | -13.457 | -0.6927 | -0.529 | H(298) | 9.809 | -2.3288 | 2.4871 | H(653) | 0.1353 | -9.2711 | 0.5836 |
| H(299) | 8.931 | 0.2811 | -1.8812 | C(654) | -16.7573 | -1.0399 | 2.0847 | H(299) | 9.6324 | -0.2903 | -1.1834 | H(654) | 3.8623 | -7.5618 | 0.6738 |
| H(300) | 8.4054 | 2.3906 | -0.9327 | C(655) | -18.0127 | -0.3894 | 2.6476 | H(300) | 9.4948 | 1.7651 | -0.0489 | C(655) | 4.1769 | -8.0176 | 4.6472 |
| H(301) | 12.6768 | 3.6256 | 3.0013 | O(656) | -15.7782 | -0.0527 | 1.8419 | H(301) | 13.747 | 3.1859 | 3.5047 | C(656) | 2.8043 | -8.6391 | 4.4533 |
| H(302) | 10.488 | 2.7811 | 3.375 | H(657) | -16.3213 | -1.7696 | 2.8029 | H(302) | 11.6878 | 2.0218 | 3.9317 | O(657) | 4.7375 | -7.7447 | 3.38 |
| H(303) | 11.3006 | 6.0898 | -0.0928 | H(658) | -16.9706 | -1.5469 | 1.1175 | H(303) | 11.87 | 5.6539 | 0.6833 | H(658) | 4.0986 | -7.0588 | 5.204 |
| H(304) | 6.9369 | 3.1935 | 5.0192 | H(659) | -18.7796 | -1.1583 | 2.8966 | H(304) | 8.3772 | 2.2853 | 6.0862 | H(659) | 4.8627 | -8.7072 | 5.187 |
| H(305) | 6.8079 | 1.422 | 6.5773 | H(660) | -18.4647 | 0.3095 | 1.908 | H(305) | 7.8469 | 0.4219 | 7.4485 | H(660) | 2.3244 | -8.8606 | 5.4338 |
| H(306) | 8.9899 | -1.0913 | 4.0029 | H(661) | -17.7871 | 0.1836 | 3.5761 | H(306) | 9.2187 | -2.2167 | 4.4769 | H(661) | 2.8712 | -9.5904 | 3.8776 |
| H(307) | 9.918 | -1.8349 | -1.7593 | H(662) | -14.9447 | -0.4653 | 1.6006 | H(307) | 10.0867 | -2.5746 | -1.2895 | H(662) | 2.1434 | -7.9384 | 3.8947 |
| H(308) | 7.938 | -0.6758 | 7.4566 | C(663) | -10.9969 | 0.0267 | 0.9126 | H(308) | 8.3339 | -2.0149 | 7.9731 | H(663) | 4.059 | -7.364 | 2.8147 |
| H(309) | 13.4592 | 6.5481 | 1.072 | C(664) | -11.6116 | -0.8864 | 1.9662 | H(309) | 14.0278 | 6.2999 | 1.7342 | C(664) | 5.7536 | -9.7686 | 1.0201 |
| H(310) | 14.1694 | 5.2288 | 2.0392 | O(665) | -11.5564 | -0.228 | -0.3618 | H(310) | 14.926 | 5.0638 | 2.6461 | C(665) | 5.7547 | -10.2562 | -0.4217 |
| H(311) | 13.365 | 4.9959 | -0.9466 | H(666) | -11.1566 | 1.1021 | 1.1434 | H(311) | 14.1344 | 4.8658 | -0.3444 | O(666) | 5.8531 | -8.3599 | 1.0325 |
| H(312) | 15.0173 | 5.307 | -0.3832 | H(667) | -9.905 | -0.1692 | 0.8582 | H(312) | 15.7371 | 5.3058 | 0.2636 | H(667) | 4.8161 | -10.0502 | 1.5486 |
| H(313) | 15.3854 | 3.1521 | 0.3763 | H(668) | -12.6972 | -0.6967 | 2.1057 | H(313) | 16.3699 | 3.1447 | 0.5908 | H(668) | 6.6199 | -10.1726 | 1.5884 |
| H(314) | 13.7097 | 2.8631 | 0.8793 | H(669) | -11.121 | -0.7324 | 2.9546 | H(314) | 14.9217 | 2.7243 | 1.5098 | H(669) | 5.6883 | -11.3673 | -0.4649 |
| H(315) | 14.5767 | 2.9088 | -2.0795 | H(670) | -11.4905 | -1.9591 | 1.6938 | H(315) | 15.0709 | 2.8032 | -1.5752 | H(670) | 6.6875 | -9.9457 | -0.9462 |
| H(316) | 14.4902 | 1.4486 | -1.0424 | H(671) | -10.9563 | 0.0119 | -1.0817 | H(316) | 15.237 | 1.3496 | -0.5416 | H(671) | 4.8906 | -9.84 | -0.9878 |
| H(317) | 12.0062 | 1.9453 | -0.6053 | C(672) | -12.4899 | 0.4737 | -6.9021 | H(317) | 12.8726 | 1.781 | 0.3777 | H(672) | 5.9649 | -8.0483 | 1.934 |
| H(318) | 12.1151 | 3.31 | -1.7468 | C(673) | -11.0703 | 0.3518 | -7.4367 | H(318) | 12.7215 | 3.1416 | -0.7704 | C(673) | -14.3167 | -3.5045 | -0.1442 |
| H(319) | 11.1568 | 1.446 | -2.9361 | O(674) | -12.6932 | -0.4843 | -5.8865 | H(319) | 11.6234 | 1.3085 | -1.8331 | C(674) | -14.8852 | -4.2977 | 1.0227 |
| H(320) | 12.8143 | 1.6855 | -3.5814 | H(675) | -12.6664 | 1.4734 | -6.4482 | H(320) | 13.1871 | 1.4849 | -2.6903 | O(675) | -14.4402 | -2.1172 | 0.0968 |
| H(321) | 11.9976 | -0.4215 | -1.4524 | H(676) | -13.2452 | 0.2898 | -7.6986 | H(321) | 12.5139 | -0.5657 | -0.4631 | H(676) | -13.2334 | -3.7196 | -0.2716 |
| H(322) | 13.6245 | -2.6258 | -3.1187 | H(677) | -10.8866 | 1.0953 | -8.2458 | H(322) | 14.0157 | -2.8367 | -2.1183 | H(677) | -14.8518 | -3.7382 | -1.0914 |
| H(323) | 12.0451 | -2.8704 | -2.2973 | H(678) | -10.8828 | -0.6645 | -7.8524 | H(323) | 12.4628 | -2.9963 | -1.2313 | H(678) | -14.7189 | -5.3896 | 0.8762 |
| H(324) | 8.5921 | 7.7154 | -3.0036 | H(679) | -10.3219 | 0.5376 | -6.6342 | H(324) | 8.9099 | 6.8325 | -2.1433 | H(679) | -15.9825 | -4.1383 | 1.1198 |
| H(325) | 6.934 | 7.207 | -3.4342 | H(680) | -12.0311 | -0.3624 | -5.2007 | H(325) | 7.5724 | 5.8333 | -2.7759 | H(680) | -14.3992 | -4.0053 | 1.9817 |
| H(326) | 6.0174 | 8.6541 | -1.5619 |  |  |  |  | H(326) | 5.9415 | 6.9862 | -1.255 | H(681) | -13.8341 | -1.6451 | -0.4851 |
| H(327) | 7.6867 | 9.1908 | -1.1624 |  |  |  |  | H(327) | 7.2831 | 7.9049 | -0.4848 | C(682) | -16.4022 | -1.5547 | 2.755 |
| H(328) | 7.8929 | 10.248 | -3.4354 |  |  |  |  | H(328) | 7.779 | 9.0069 | -2.7162 | C(683) | -17.4809 | -0.813 | 3.5308 |
| H(329) | 6.2582 | 9.6428 | -3.8719 |  |  |  |  | H(329) | 6.3901 | 8.1169 | -3.4368 | O(684) | -15.4749 | -0.6302 | 2.2283 |
| H(330) | 5.3106 | 10.9183 | -1.8499 |  |  |  |  | H(330) | 4.8583 | 9.1938 | -1.7006 | H(685) | -15.8373 | -2.2555 | 3.4093 |
| H(331) | 6.9421 | 11.6229 | -1.5878 |  |  |  |  | H(331) | 6.2558 | 10.1224 | -1.0701 | H(686) | -16.8416 | -2.1139 | 1.8998 |
| H(332) | 6.8368 | 12.627 | -3.9306 |  |  |  |  | H(332) | 6.5616 | 11.2448 | -3.2889 | H(687) | -18.1953 | -1.5286 | 3.9985 |
| H(333) | 5.1904 | 11.9244 | -4.132 |  |  |  |  | H(333) | 5.2247 | 10.2675 | -3.9893 | H(688) | -18.065 | -0.1449 | 2.8581 |
| H(334) | 4.4216 | 13.2244 | -2.0774 |  |  |  |  | H(334) | 3.6136 | 11.2691 | -2.338 | H(689) | -17.0373 | -0.1912 | 4.3419 |
| H(335) | 6.0552 | 13.972 | -1.9754 |  |  |  |  | H(335) | 4.9407 | 12.2586 | -1.6316 | H(690) | -14.7375 | -1.0995 | 1.83 |
| H(336) | 5.7411 | 14.9771 | -4.2628 |  |  |  |  | H(336) | 5.2556 | 13.4065 | -3.8533 | C(691) | -11.164 | -1.0013 | 0.6542 |
| H(337) | 4.1139 | 14.2162 | -4.3776 |  |  |  |  | H(337) | 3.9484 | 12.3943 | -4.5665 | C(692) | -11.7341 | -1.7685 | 1.8395 |
| H(338) | 3.3639 | 15.5018 | -2.3338 |  |  |  |  | H(338) | 2.2972 | 13.343 | -2.911 | O(693) | -11.9671 | -1.1678 | -0.4944 |
| H(339) | 4.9878 | 16.2639 | -2.2147 |  |  |  |  | H(339) | 3.5975 | 14.3472 | -2.1796 | H(694) | -11.0846 | 0.0871 | 0.8592 |
| H(340) | 3.3544 | 17.8016 | -3.361 |  |  |  |  | H(340) | 2.2198 | 15.6966 | -3.8018 | H(695) | -10.1581 | -1.3899 | 0.394 |
| H(341) | 4.6612 | 17.3203 | -4.5008 |  |  |  |  | H(341) | 3.9129 | 15.5506 | -4.3937 | H(696) | -12.7205 | -1.3711 | 2.1599 |
| H(342) | 3.0332 | 16.5559 | -4.6194 |  |  |  |  | H(342) | 2.6107 | 14.5418 | -5.1255 | H(697) | -11.0504 | -1.701 | 2.7163 |
| O(343) | 12.0106 | -7.6748 | 1.8181 |  |  |  |  | O(343) | 11.5617 | -8.7464 | 2.0786 | H(698) | -11.872 | -2.8463 | 1.6008 |
| H(344) | 12.4989 | -6.9244 | 2.1168 |  |  |  |  | H(344) | 12.0807 | -8.0918 | 2.5179 | H(699) | -11.4921 | -0.8799 | -1.2854 |
| H(345) | 5.734 | -6.9062 | -0.7849 |  |  |  |  | H(345) | 5.5974 | -7.4964 | -0.677 | C(700) | -12.8123 | -0.7572 | -6.9154 |
| H(346) | 1.953 | -5.0379 | 4.1883 |  |  |  |  | H(346) | 1.3045 | -4.6422 | 5.8033 | C(701) | -11.4984 | -1.1683 | -7.5638 |
| O(347) | 7.9417 | -7.6443 | 7.7882 |  |  |  |  | O(347) | 7.1822 | -8.6047 | 7.8485 | O(702) | -13.1662 | -1.6984 | -5.9246 |
| H(348) | 7.5539 | -8.1495 | 7.0941 |  |  |  |  | H(348) | 6.7836 | -9.0191 | 7.1024 | H(703) | -12.7202 | 0.2306 | -6.4126 |
| O(349) | 5.0415 | -6.4719 | -1.2544 |  |  |  |  | O(349) | 4.9429 | -7.0248 | -1.1638 | H(704) | -13.6397 | -0.7188 | -7.6587 |
| O(350) | 2.188 | -4.5389 | 4.9534 |  |  |  |  | O(350) | 2.1602 | -4.8815 | 5.4929 | H(705) | -11.1975 | -0.4353 | -8.347 |
| H(351) | -6.8688 | -2.527 | -5.5829 |  |  |  |  | H(351) | -7.3247 | -3.5822 | -5.5055 | H(706) | -11.5831 | -2.1701 | -8.0435 |
| H(352) | -5.6335 | -0.5729 | -6.0218 |  |  |  |  | H(352) | -6.194 | -1.5912 | -6.0837 | H(707) | -10.6771 | -1.2151 | -6.8141 |
| H(353) | -8.5955 | 1.8144 | -4.2684 |  |  |  |  | H(353) | -9.2393 | 0.7368 | -4.4164 | H(708) | -12.5129 | -1.6729 | -5.2196 |
| H(354) | -6.0072 | -4.3487 | -0.9742 |  |  |  |  | H(354) | -6.316 | -5.095 | -0.8289 |  |  |  |  |
| H(355) | -9.3059 | -2.6719 | 0.998 |  |  |  |  | H(355) | -9.773 | -3.78 | 1.1436 |  |  |  |  |

**References**

Ambapuram, M., Ramireddy, R., Maddala, G., Godugunuru, S., Yerva, P. V. S., and Mitty, R. (2020). Effective Upconverter and Light Scattering Dual Function LiYF 4 :Er 3+ /Yb 3+ Assisted Photoelectrode for High Performance Cosensitized Dye Sensitized Solar Cells. *ACS Appl. Electron. Mater.* 2, 962–970. doi:10.1021/acsaelm.0c00014.

Dehghani, H. (2013). Electrochimica Acta Enhancement of dye-sensitized solar cells performances by improving electron density in conduction band of nanostructure TiO 2 electrode with using a metalloporphyrin as additional dye. *Electrochim. Acta* 92, 315–322. doi:10.1016/j.electacta.2013.01.055.

Elangovan, R., and Venkatachalam, P. (2015). Co-sensitization Promoted Light Harvesting for Dye-Sensitized Solar Cells. *J. Inorg. Organomet. Polym. Mater.* 25, 823–831. doi:10.1007/s10904-015-0165-x.

Kumar, K. A., Subalakshmi, K., and Senthilselvan, J. (2019). Materials Science in Semiconductor Processing Effect of co-sensitization in solar exfoliated TiO 2 functionalized rGO photoanode for dye-sensitized solar cell applications. *Mater. Sci. Semicond. Process.* 96, 104–115. doi:10.1016/j.mssp.2019.02.027.

Li, J., Zhao, L., Wang, S., Hu, J., Dong, B., Lu, H., et al. (2013). Great improvement of photoelectric property from co-sensitization of TiO 2 electrodes with CdS quantum dots and dye N719 in dye-sensitized solar cells. *Mater. Res. Bull.* 48, 2566–2570. doi:10.1016/j.materresbull.2013.03.009.

Lim, J., Kwon, Y. S., and Park, T. (2011). Effect of coadsorbent properties on the photovoltaic performance of dye-sensitized solar cells. *Chem. Commun.* 47, 4147–4149. doi:10.1039/c0cc04999a.

Luo, J., Wan, Z., Jia, C., Wang, Y., and Wu, X. (2016). Electrochimica Acta Co-sensitization of Dithiafulvenyl-Phenothiazine Based Organic Dyes with N719 for Ef fi cient Dye-Sensitized Solar Cells. *Electrochim. Acta* 211, 364–374. doi:10.1016/j.electacta.2016.05.175.

Mazloum-Ardakani, M., and Arazi, R. (2019). Improving the effective photovoltaic performance in dye-sensitized solar cells using an azobenzenecarboxylic acid-based system. *Heliyon* 5, e01444. doi:10.1016/j.heliyon.2019.e01444.

Naik, P., Su, R., El-shafei, A., and Vasudeva, A. (2017). Improved photovoltaic performances of Ru ( II ) complex sensitized DSSCs by co-sensitization of carbazole based chromophores. *Inorg. Chem. Commun.* 86, 241–245. doi:10.1016/j.inoche.2017.10.030.

Naik, P., Su, R., Elmorsy, M. R., El-shafei, A., and Vasudeva, A. (2018). Dyes and Pigments Investigation of new carbazole based metal-free dyes as active photo- sensitizers / co-sensitizers for DSSCs. *Dye. Pigment.* 149, 177–187. doi:10.1016/j.dyepig.2017.09.068.

Ooyama, Y., Uenaka, K., Kanda, M., Yamada, T., Shibayama, N., and Ohshita, J. (2015). A new co-sensitization method employing D-π-A dye with pyridyl group and D-π-Cat dye with catechol unit for dye-sensitized solar cells. *Dye. Pigment.* 122, 40–45. doi:10.1016/j.dyepig.2015.06.009.

Ren, X., Feng, Q., Zhou, G., Huang, C. H., and Wang, Z. S. (2010). Effect of cations in coadsorbate on charge recombination and conduction band edge movement in dye-sensitized solar cells. *J. Phys. Chem. C* 114, 7190–7195. doi:10.1021/jp911630z.

Sun, J., Guo, H., Zhao, L., Wang, S., Hu, J., and Dong, B. (2017). Co-sensitized efficient dye-sensitized solar cells with TiO2 hollow sphere/nanoparticle double-layer film electrodes by Bi2S3 quantum dots and N719. *Int. J. Electrochem. Sci.* 12, 7941–7955. doi:10.20964/2017.09.01.

Wu, Z., Song, X., Liu, Y., Zhang, J., Wang, H., Chen, Z., et al. (2020). New organic dyes with varied arylamine donors as effective co-sensitizers for ruthenium complex N719 in dye sensitized solar cells. *J. Power Sources* 451, 227776. doi:10.1016/j.jpowsour.2020.227776.

Younas, M., and Harrabi, K. (2020). Performance enhancement of dye-sensitized solar cells via co-sensitization of ruthenium ( II ) based N749 dye and organic sensitizer RK1. *Sol. Energy* 203, 260–266. doi:10.1016/j.solener.2020.04.051.

Zhang, J., Fu, C., Yang, X., and Cao, W. (2011). Study on the Performance of Zn,N/TiO 2 Anode Film and Co-Sensitization in DSSC. *J. Inorg. Organomet. Polym. Mater.* 21, 43–49. doi:10.1007/s10904-010-9416-z.
